# Modulation of the transcription regulatory program in yeast cells committed to sporulation

**DOI:** 10.1186/gb-2006-7-3-r20

**Published:** 2006-03-08

**Authors:** Gilgi Friedlander, Daphna Joseph-Strauss, Miri Carmi, Drora Zenvirth, Giora Simchen, Naama Barkai

**Affiliations:** 1 Departments of Molecular Genetics and Physics of Complex System, Weizmann Institute of Science, Rehovot 76100, Israel; 2 Department of Genetics, The Hebrew University of Jerusalem, Jerusalem 91904, Israel

## Abstract

**Background:**

Meiosis in budding yeast is coupled to the process of sporulation, where the four haploid nuclei are packaged into a gamete. This differentiation process is characterized by a point of transition, termed commitment, when it becomes independent of the environment. Not much is known about the mechanisms underlying commitment, but it is often assumed that positive feedback loops stabilize the underlying gene-expression cascade.

**Results:**

We describe the gene-expression program of committed cells. Sporulating cells were transferred back to growth medium at different stages of the process, and their transcription response was characterized. Most sporulation-induced genes were immediately downregulated upon transfer, even in committed cells that continued to sporulate. Focusing on the metabolic-related transcription response, we observed that pre-committed cells, as well as mature spores, responded to the transfer to growth medium in essentially the same way that vegetative cells responded to glucose. In contrast, committed cells elicited a dramatically different response.

**Conclusion:**

Our results suggest that cells ensure commitment to sporulation not by stabilizing the process, but by modulating their gene-expression program in an active manner. This unique transcriptional program may optimize sporulation in an environment-specific manner.

## Abstract 2

Analysis of the gene expression program in yeast cells suggests that commitment to sporulation involves an active modulation of the gene expression program.

## Background

Meiosis is a specialized cell division by which haploid gametes are generated from diploid cells. The principal features of meiosis are common to all eukaryotic organisms and include a single round of DNA replication ('premeiotic' replication) followed by two consecutive nuclear divisions, meiosis I and meiosis II. In the first meiotic division homologous chromosomes segregate to opposite poles, whereas in the second division the two sister chromatids separate from each other. Meiosis is characterized by a high frequency of recombination events, occurring during a prolonged prophase that separates DNA replication from the first meiotic division. This genetic exchange between homologous chromosomes ensures that they segregate properly and that the offspring differ genetically from their parents and from each other.

**Figure 1:**
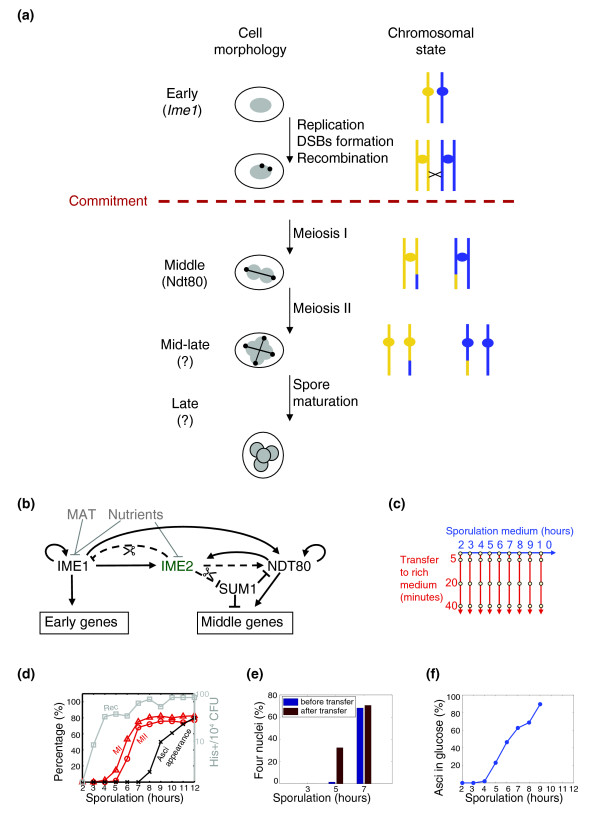
Experimental design. **(a) **Meiotic landmarks. The point of commitment is indicated. DSBs, double-strand breaks. **(b) **The regulatory network underlying the sporulation gene-expression program. Known interactions are shown. Arrows denote activation, and barred lines represent inhibition. Solid lines indicate regulation on the level of transcription while dashed lines indicate post-transcriptional regulation (for example, by protein phosphorylation). Transcription factors are shown in black and the kinase in green. The input of the cascade is shown in gray and scissors indicate degredation. IME2 activates middle gene expression, at least in part, by relieving Sum1-mediated repression of *NDT80 *[34]. **(c) **The experimental design. The sporulation process was initiated by transferring cells to sporulation medium. Cells were allowed to progress through the process for varying lengths of times, and were then transferred back to rich nutrient-containing medium. Each circle represents a time point at which genome-wide gene expression was monitored. **(d) **Temporal progression of sporulation. The percentages of cells that completed the first meiotic division (MI, triangles) or the second meiotic division (MII, circles) are shown in red, the percentage of asci in black and the recombination frequencies (Rec, determined by the frequency of His^+ ^cells) in gray. CFU, colony-forming units. **(e,f) **Commitment to sporulation. (e) Cells were transferred to YPD at different stages of sporulation and were followed in YPD until 8 hours after sporulation initiation. The percentage of cells with four nuclei (determined by DAPI staining) before and after the transfer is shown. The time of transfer from sporulation medium (SPM) is indicated. (f) Cells were transferred from SPM to glucose solution (4%) at various times, as indicated. For each glucose culture, we calculated the fraction of cells that became spores, 24 hours after the initiation of the sporulation process (normalized by the sporulation efficiency at that experiment, which was 80%). Cells that were transferred early (before 5 hours in SPM) arrested in the cell cycle, as glucose alone does not support growth. At later times, cells continued the sporulation process and generated spores. Commitment occurs at around 5-6 hours in SPM.

The meiotic process is coupled to a program of cellular differentiation, which ultimately packages the haploid nuclei into gametes. In the budding yeast *Saccharomyces cerevisiae*, meiosis is coupled to the process of sporulation, in which the four haploid nuclei are packaged into spores (Figure 1a). In this organism, diploid cells initiate meiosis when starved for glucose and nitrogen. Starvation signals as well as diploidy induce the transcription of *IME1*, which functions as a master regulator of the sporulation process [1-5]. By activating meiotic regulators, Ime1 initiates a transcription cascade (Figure 1b). In addition, Ime1 directly induces the first wave of meiotic gene induction, consisting of genes involved in early meiotic events, such as DNA replication, recombination, and synaptonemal complex formation. The second wave of gene induction is observed at mid-sporulation, at about the time when the cells initiate the first meiotic division, and includes genes involved in the two meiotic divisions and spore-wall formation. Notably, the principal regulator of mid-sporulation genes, Ndt80, autoactivates its own expression [6]. Genome-wide assessment of transcription during sporulation in yeast has revealed more than 1,000 genes whose mRNA levels were significantly induced or repressed during the process [7,8]. High-throughput loss-of-function studies have shown that some, but not all, of these induced genes are indeed essential for meiosis and spore formation [9-11].

Diploid yeast cells undergoing early stages of meiosis and sporulation may return to the mitotic cell division if provided with nutrients, especially glucose and a nitrogen source. These return-to-growth (RTG) cells complete some meiotic processes, such as high-frequency recombination [12,13], but switch to the mitotic mode of chromosome segregation and produce diploid cells, following a single division. It is not yet clear how RTG cells resolve the high frequency of double-strand breaks to ensure chromosomal fidelity, and how the cells reinitiate the mitotic cell-cycle program after DNA replication but before chromosomal separation.

RTG may occur only up to a certain stage in the sporulation process. Beyond this stage, cells will continue with sporulation events even if challenged with rich nutritional conditions. This stage of irreversibility was termed "commitment to meiosis" [14]; it occurs after premeiotic DNA replication and recombination have taken place, but before meiosis I. This commitment is probably essential for ensuring cell viability, as the sporulation process involves drastic changes in morphology, chromosomal state and cell-wall composition, which may be hazardous if abandoned before completion.

The molecular mechanism underlying commitment is not understood. It was proposed that commitment to the meiotic process involves the separation of spindle pole bodies (SPBs) [15]. Indeed, SPB separation was shown to correlate with commitment to mitosis [16]. Conditions that impair late sporulation events may also impact on commitment [17]. In particular, cells mutated in the *SPO14 *gene, which codes for phospholipase D and is required for late sporulation events, are able to return to growth even after completing meiosis I [17]. The role of phospholipase D-dependent signaling in the progression of the meiotic program is not yet clear.

To better understand the process of commitment to meiosis, we examined the genome-wide transcription response triggered by the transfer of sporulating cells to rich growth medium at different stages of the process. At all stages, the transfer initiated large-scale changes in the gene-expression pattern. The majority of genes that were induced during sporulation were immediately repressed upon the transfer to growth medium, even in committed cells that continued to sporulate. At the same time, committed cells displayed a unique response to nutrients, which was dramatically different from that observed in all other cell types examined (pre-committed cells, spores or vegetative cells exposed to glucose). This unique response consisted of metabolic-related genes, as well as genes involved in competing developmental processes such as pseudo-hyphal growth. Our findings suggest that commitment to meiosis is achieved not by stabilizing the transcription cascade, but rather by an active modulation of the gene-expression program, the detailed nature of which is only starting to be unraveled.

## Results

### Experimental design

To examine the transcription program associated with commitment to sporulation, we employed the RTG experimental paradigm [12,18]. The sporulation process was initiated by transferring cells to sporulation medium (SPM). Cells were allowed to progress through the process for varying lengths of times, and were then transferred back to nutrient-containing media (Figure 1c). Cells that were transferred early, before the initiation of the first meiotic division, grew buds and resumed mitotic divisions. In contrast, most cells transferred at later stages continued the sporulation process to produce spores [12,14,18] (Figure 1d-e).

To define the time of commitment, we first followed meiotic landmarks events (Figure 1d). Previous studies associated commitment with a time before the completion of meiosis I, which in our conditions occurred at around 5-6 hours in SPM. Indeed, cells that were transferred to yeast extract/peptone/dextrose medium (YPD) after five hours in SPM continued through the second meiotic division also in YPD (Figure 1e). As a more direct assay of commitment, we transferred cells from SPM to a solution containing 4% glucose (see Materials and methods). Glucose is a potent inhibitor of sporulation initiation, and indeed, cells that were transferred early (less than five hours in SPM) abandoned the sporulation program and arrested the cell cycle. In contrast, most cells transferred at a later stage, after being more than five hours in SPM, continued the sporulation process and generated mature spores (Figure 1f). Taken together, we conclude that commitment occurs at around 5-6 hours in SPM. Significantly, mature spores began to appear only at about 8 hours in SPM, reaching the maximum percentage at about 12 hours in SPM (Figure 1d). Thus, the sporulation process continued in rich medium for a considerable period of time before cells became mature spores.

**Figure 2:**
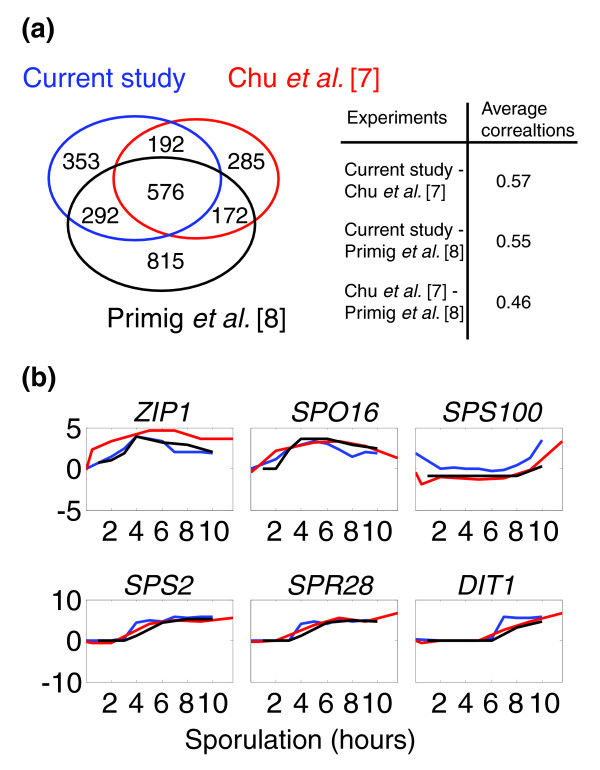
Comparison with previous studies. **(a) **Venn diagram comparing the genes induced during sporulation in our experiment and in two previous experiments [7,8]. A gene was defined as 'induced' at a particular time point, if its expression level at that time point was at least twofold higher than that of the pre-sporulation reference. All genes that were induced in at least one of the sporulation time points were considered (the area in the Venn diagram is proportional to the number of genes [63]). The average correlations between pairs of experiments are indicated (see Materials and methods for how correlations were calculated). **(b) **Examples of expression profiles for specific genes obtained in the current study (blue) and in the previous studies (red [7] and black [8]).

We used DNA microarrays representing the full yeast genome to characterize the genome-wide expression profile at subsequent time points following the transfer of sporulating cells to rich growth medium (YPD, Figure 1c). We examined also the gene-expression profile before the transfer, during the sporulation process itself, and compared it with the corresponding transcription program characterized in two previous reports (Figure 2); Of the approximately 1,400 genes that were induced more than twofold during sporulation in our experiments, 576 were induced in both previous studies, and 484 additional genes were induced in just one of these studies. Notably, the number of genes that were induced only in our experiment (about 350) is comparable to the number of genes identified uniquely by one of the studies (285) [7] and is significantly lower then the number of genes identified uniquely by the other (815) [8]. Moreover, the overall correlation between all three experimental time courses is highly significant, with the highest correlation found between our study and the study of Chu *et al*. [7] (Figure 2a,b, and see Materials and methods for calculation of these correlations).

The transfer of sporulating cells to YPD led to a large-scale change in gene expression. Following the transfer, about 1,000 genes were induced by at least twofold. The identity of the induced genes differed between early or late sporulating cells (see below). The number of repressed genes varied somewhat between early and late sporulating cells: around 1,200 genes were repressed over twofold in cells that were transferred early, whereas only around 480 genes were repressed when cells were transferred later in the sporulation process. Evidently, sporulating cells sense and respond to the growth medium at all stages of the sporulation process, even after they have become committed to its completion.

### The return to growth (RTG) transcription program

**Figure 3:**
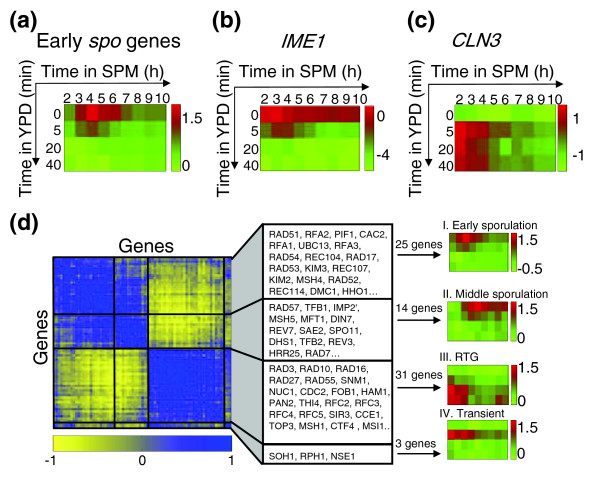
Transcriptional response of return to growth (RTG) cells. SPM, sporulation medium; YPD, growth medium. **(a) **The expression pattern of early sporulation (*spo*) genes (61 early I genes, as defined in [7]). Note the immediate repression of these genes upon transfer to growth medium (see Additional data file 3 for early genes defined according to the data presented in the current study; see Additional data file 5 for a matlab program that enables the reader to view the data in that format). **(b) **Expression pattern of *IME1*, the regulator of early-sporulation genes. **(c) **Expression pattern of the G1 cyclin gene, *CLN3*. In (a-c), expression patterns are shown as log_2 _ratios, and are color-coded for the log_2 _fold change according to the bar shown. **(d) **Polarized expression of genes associated with DNA repair and DNA recombination. The matrix of pairwise correlations between genes assigned to the GO groups 'DNA recombination' and 'DNA repair' is plotted. The genes were clustered according to similarity in their Pearson correlations, calculated on the basis of their expression patterns in our experiment (see Materials and methods). The average expression pattern of genes in each cluster is shown on the right. The first cluster include genes expressed during early sporulation, the second includes genes induced during middle sporulation, the third shows genes induced during RTG, and the fourth genes that are transiently induced on transfer to YPD and then repressed. The number of genes in each cluster is indicated above the arrow. Some of the genes in each cluster are indicated (for a full list of genes, and for a similar representation of additional gene classes, see Additional data files 22 and 33). Correlations are color-coded according to the bar shown.

Cells that were exposed to growth medium during the early stages of meiosis returned to mitotic growth. As expected, these cells responded to YPD by immediately downregulating virtually all genes that were induced as part of the early meiotic program (Figure 3a). Early meiotic genes are directly induced by the transcription factor Ime1 [3], and their downregulation is probably a direct consequence of the glucose-dependent repression of *IME1 *(Figure 3b) [1]. The reduction in *IME1 *expression during sporulation from a peak at three hours seems moderate compared with results from northern analyses [1,19], but is consistent with previous microarray experiments [7,8]. These differences may be due to the different sensitivity of microarray versus northern analysis.

To try and infer the stage through which the cells re-enter the mitotic cell cycle, we examined the response of genes known to be regulated during different phases of the mitotic cell cycle [20,21]. The G1 cyclin gene *CLN3 *was immediately induced on the addition of YPD (Figure 3c). Indeed, Cln3 promotes entry to the cell cycle [22] and serves as a negative regulator of meiotic initiation [23,24]. Its induction during the return to growth may thus assist in switching from meiosis to mitotic growth. None of the other cyclins or cyclin-dependent kinases was similarly induced. The overall pattern of gene induction, however, was hard to interpret as it did not resemble any of the mitotic cell-cycle phases. For example, although a significant portion of the genes that are upregulated during either G1 or the M phase of the mitotic cycle were induced also during RTG, others, with similar expression patterns during the mitotic cycle, were not (see Additional data file 3). A likely explanation is that individual cells induce different cell-cycle genes depending on which stage of meiosis they are coming from, and this mixture of cell-cycle genes reflects the incomplete synchronization of our culture. In support of that interpretation, genes that are induced during the S phase of the mitotic cell cycle were induced during RTG only when the transfer was done early enough (two hours in SPM) but not later (see Additional data file 3).

In addition to reprogramming their gene expression, RTG cells need to resolve meiosis-specific events and structures. For example, the meiosis-specific alignment of homologous chromosomes during the first meiotic division [25-27] does not exist during mitosis. Indeed, the synaptonemal complex (SC) structures, associated with meiotic pairing, disappear rapidly upon RTG [13]. Moreover, meiosis is characterized by a high level of post-replication double-strand breaks (DSBs), which are required for initiating recombination events. Single-gene studies indicated that in addition to the meiotic repair pathways, additional mitotic DNA repair pathways are used to resolve those breaks during RTG [13]. Not much is known about the processes that are involved in the RTG program, however.

To characterize RTG pathways we analyzed the expression pattern of genes associated with DNA-related processes [28]. Interestingly, most such genes were induced either during sporulation or during the return to the mitotic cell cycle, with only a few induced during both processes (see, for example, Figure 3d). It is likely that genes that are induced during RTG, such as *HAM1*, *RAD55*, and *MSH1*, participate in this process. A comprehensive classification of genes according to their time of induction is provided in Additional data file 3. The classification of homologous genes is also given in Additional data file 3.

### Downregulation of sporulation-specific genes in committed cells

**Figure 4:**
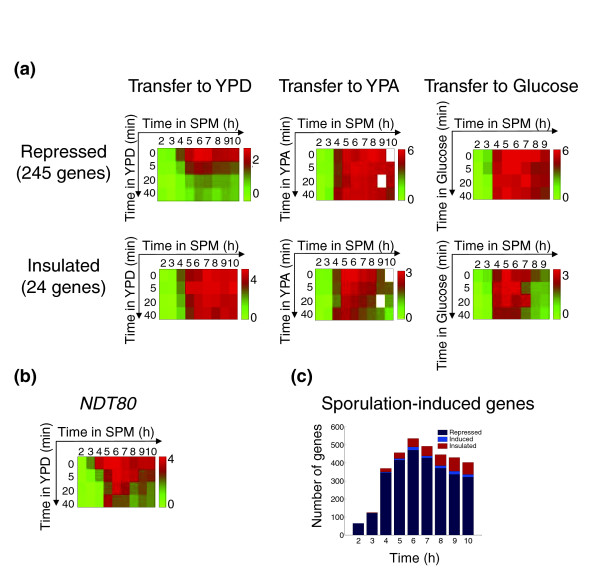
Transcriptional response of committed cells. **(a) **Average expression of middle sporulation genes. The repressed group (245 genes) and the insulated group (24 genes) are shown. Note that downregulation of the repressed group is specific to YPD, and is not observed on transfer to YPA (which contains nitrogen and acetate) or glucose (4% solution). **(b) **Expression pattern of *NDT80*. Expression patterns in (a) and (b) are shown in log_2 _ratios, as in Figure 3, and log_2 _fold change in expressionis color-coded according to the bar. **(c) **A summary of the behavior of all sporulation-induced genes. All genes induced during sporulation were considered. A gene was defined as induced at a certain time point during sporulation if it was upregulated more than twofold in that time as well as in the previous and following hours. The induced genes were classified into three categories, depending on their behavior on transfer to YPD: repressed, induced, and insulated. The 'repressed' category included genes that were downregulated by at least 1.5-fold when transferred compared with their level during sporulation. Similarly, the 'induced' category included genes whose expression was upregulated by at least 1.5-fold on transfer. The 'insulated' group included the genes whose expression remained stable (did not change) upon the transfer (expression after transfer to YPD was less than 1.3-fold relative to sporulation).

In contrast to early meiotic cells, which responded to YPD by returning to mitotic growth, cells at later stages of sporulation continued to sporulate in rich medium (Figure 1e-f) [14,18]. Surprisingly, also in these committed cells, the exposure to YPD led to a rapid downregulation of most sporulation-specific genes. For example, of the 269 genes that were induced more than twofold during mid-sporulation, around 125 were downregulated within the first 10 minutes of transfer to YPD, and around 100 additional genes were downregulated within the next 30 minutes (data not shown). Only 24 genes (9%), most of which associated with spore-wall biogenesis, maintained stable expression for longer times (Figure 4a, and see Additional data file 3).

Large-scale phenotypic studies have shown that only a portion of the genes that are induced during sporulation are also required for the process [9,29]. One possibility is that the sporulation-induced genes that were repressed upon the transfer to YPD are not in fact required for sporulation. To test this possibility, we examined in detail the properties of the repressed genes. This set included numerous cell-cycle genes that are required for the two consecutive meiotic divisions [30-32], such as genes coding for most components of the anaphase-promoting complex (APC), its activator Cdc20, the B-type cyclins Clb1,3-6, the polo-like kinase Cdc5, the securin Pds1, the gene *CDC15 *and the kinesin-like coding gene *KAR3 *(see Additional data file 3). Several genes involved in spore-wall formation were also identified. Thus, many of the genes that were immediately repressed upon the addition of YPD have a well-established role in sporulation.

Moreover, the major meiotic regulator genes such as *IME2 *and *SMK1*, were also repressed upon transfer to rich medium (see Additional data file 2). In particular, *NDT80*, the master regulator of the mid-sporulation genes, was downregulated as well (Figure 4b). This indicates that the capacity of Ndt80 to autoactivate its gene expression is not sufficient for stabilizing its expression on exposure to rich growth medium. Interestingly, neither glucose alone nor acetate- and nitrogen-containing growth medium (yeast extract/peptone/potassium acetate (YPA)) were sufficient to cause this downregulation, indicating a combinatorial requirement for both factors (Figure 4a).

### Downregulation of sporulation-induced genes in committed cells is independent of *NDT80 *or *SUM1*

**Figure 5:**
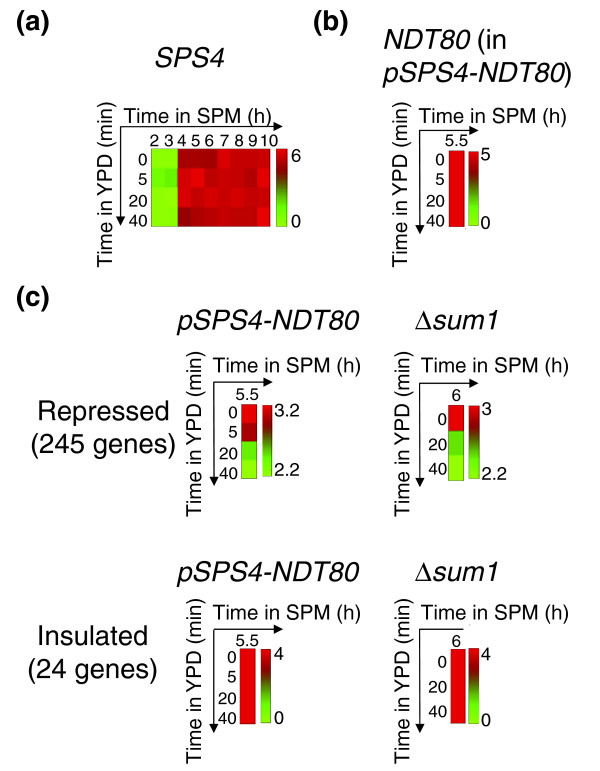
Response of mid-sporulation genes to YPD in *pSPS4-NDT80 *and Δ*sum1 *strains. **(a) **Expression pattern of *SPS4 *in wild-type cells. **(b) **The expression of *NDT80 *in *pSPS4-NDT80 *cells. Cells were incubated for 5.5 hours in SPM, and were then transferred to YPD. Gene expression is shown at different time points following the transfer, as indicated. Note that a high level of *NDT80 *mRNA is maintained (compare with Figure 4b, note the different times). **(c) **The average expression of mid-sporulation genes in *pSPS4-NDT80 *and in Δ*sum1 *strain. The repressed group (245 genes) and the insulated group (24 genes) are shown. Expression patterns in (a-c) are shown in log_2 _ratios, and are coded according to the colored bars.

The sporulation-specific expression of most mid-sporulation genes is induced directly by the meiotic regulator Ndt80 [6], and is inhibited by the meiotic repressor Sum1 (Figure 1b). Ndt80 and Sum1 compete for the same DNA sequence [33]. During sporulation in SPM, Ndt80 is activated, whereas Sum1 is targeted for degradation, leading to the induction of mid-sporulation genes [34]. We asked whether the downregulation of mid-sporulation genes in YPD reflects a repression of Ndt80 expression, or perhaps an accumulation of Sum1. To examine this possibility, we constructed a strain that maintained stable expression of *NDT80 *on transfer to YPD. This was done by replacing the endogenous *NDT80 *promoter with a promoter of the gene *SPS4 *(Figure 5a,b). Maintaining stable *NDT80 *expression did not eliminate the downregulation of most mid-sporulation genes (Figure 5c). Moreover, downregulation was also observed in a strain deleted of the mid-sporulation repressor gene *SUM1 *(Figure 5c). Those results indicate that the observed repression does not depend on the expression of the meiotic regulators, but may result from post-transcriptional modification of Ndt80, or from more direct effects of the growth medium.

### The general response to glucose is dramatically modified in committed cells

Taken together, our results indicated that the transfer of sporulating cells to glucose-containing growth medium altered the expression of most sporulation-specific genes, even in committed cells that proceed to become viable spores in rich medium. We next asked whether the transfer of sporulating cells to YPD altered the expression of additional genes, which are not part of the normal sporulation cascade. Such a response could reflect general effects of the growth medium. Alternatively, it could also indicate specific regulation that is important for ensuring the continuation of meiotic progression in rich medium.

Glucose is also a potent regulator of gene expression in vegetative cells. The transcription response of yeast cells to glucose was characterized in a recent study [35], showing a dramatic modification of the transcription program. Altered expression was observed for numerous genes involved in metabolic processes, as well as in protein synthesis [35]. We asked whether the addition of glucose-containing growth medium to sporulating cells invokes a similar metabolic response. To eliminate differences resulting from the response of sporulation-specific genes, we focused on 936 genes whose expression was altered by the addition of glucose to vegetative cells [35] and which showed a significant response also in our experiment.

**Figure 6:**
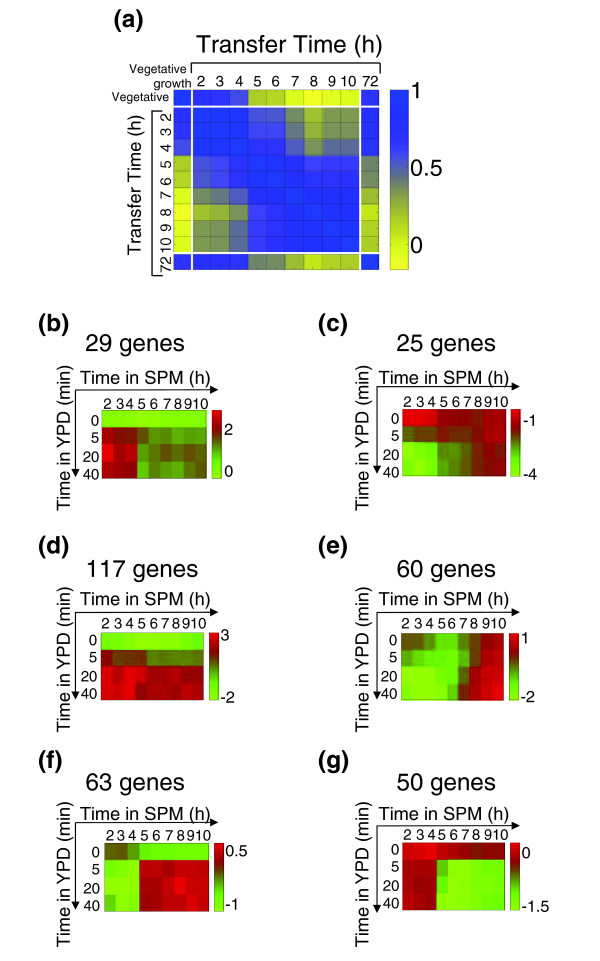
The general metabolic response to glucose. **(a) **The matrix of pairwise correlations describing the similarity in the response of cells transferred to YPD at different stages of the process is shown. We also compared these responses with the response of vegetative cells and mature spores to YPD. Correlations were calculated on the basis of 936 genes whose expression is induced after the addition of glucose to vegetative cells (see Materials and methods and Additional data file 3). A similar correlation pattern was also observed on transfer to glucose solution (see Additional data file 2). **(b-g) **Expression patterns in log_2 _ratios of specific gene groups: (b) rRNA processing genes [64] (see Additional data file 3); (c) gluconeogenesis module [64] (see Additional data file 3); (d) ribosomal proteins module [64] (see Additional data file 3); (e) 'process-specific' group (see Additional data file 3). (f,g) A group of genes that is (f) upregulated (see Additional data file 3) or (g) downregulated on transfer to YPD specifically after commitment (see Additional data file 3). See Materials and methods for the identification of groups shown in (e-g). Each expression profile is accompanied by a colored bar indicating the log_2 _fold change.

Clearly, pre-committed cells responded to rich medium in essentially the same way as vegetative cells respond to glucose (Figure 6a). In sharp contrast, cells that were transferred to rich medium after commitment, but before the completion of the sporulation process, displayed a strikingly different response (Figure 6a). For example, a large number of genes involved in rRNA processing, which are induced by glucose in vegetative cells, were not induced in committed cells (Figure 6b). Similarly, genes that are normally repressed by growth medium, such as those involved in gluconeogenesis, were not repressed in committed cells (Figure 6c). An interesting exception was the genes coding for ribosomal proteins, which were induced by YPD in all cells irrespective of their stage of sporulation (Figure 6d).

To examine whether this distinct transcription response to glucose is a property of cells maturing in the sporulation process, we also examined the response of fully developed spores, which have been in sporulation medium for three days. In those cells, the addition of rich medium initiates the process of germination. Interestingly, spores responded to rich medium in essentially the same manner as vegetative or pre-committed cells (Figure 6a). We conclude that the modified transcription response, observed in committed cells, characterizes the sporulation process itself, and not the mature spore state.

### Gene expression during sporulation in YPD

Next we asked which genes are induced specifically in committed cells on transfer to YPD. Such genes could be classified into two groups. First, a 'process-linked' group was defined. Genes in this group were induced during late meiosis in SPM (at around 8-9 hours), and were also induced in YPD, at variable times that appeared to be linked to the progression of sporulation (Figure 6e). Second, a 'commitment-specific' group was defined, which included genes that were induced by YPD specifically in committed cells (Figure 6f). Genes in this group were not induced during normal meiosis in SPM, and were also insensitive to YPD during early sporulation.

About 60 genes display a 'process-linked' expression pattern (see Figure 6e and Additional data file 3). Among these we identified two sporulation-specific genes, which are involved in spore-wall formation (*DIT1 *and *SPS100*). Surprisingly, however, most genes in this group are not in fact classified as sporulation genes, and do not have a recognized role in the process. Rather, most genes in this group are associated with the general environmental stress response (ESR), which is invoked in response to a variety of different environmental stresses [36,37]. Examples include genes coding for heat-shock proteins (such as *HSP12*, *GRE1*, and *SIP18*), for the stress-induced transcription factor Xbp1, and for the Tor1 kinase. Accordingly, the promoter regions of most of those genes contained the stress-response element CCCCT. The induction of this gene group may reflect some stress signal associated with the progression of sporulation.

The 'commitment-specific' gene group included around 65 genes that were induced by YPD specifically after commitment. In parallel, about 50 genes were repressed by YPD specifically after commitment (see Figure 6f-g and Additional data file 3). Those genes may be part of a modified transcription program that assists the continuation of the sporulation process in rich medium. Interestingly, the repressed group includes two genes that function in the pseudohyphal growth pathway (*MUC1 *and *MSB2*). It is likely that repression of these genes assists in the inhibition of this alternative developmental route.

**Figure 7:**
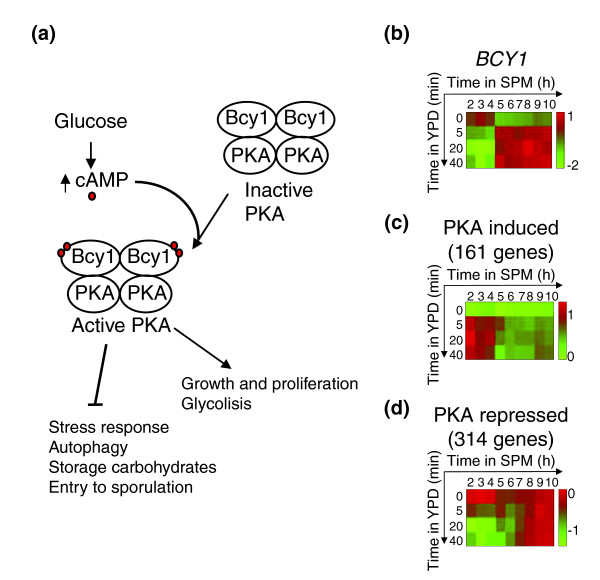
Regulation of PKA in committed cells. **(a) **The PKA pathway. The addition of glucose to a non-fermenting yeast culture results in a rapid increase in the cellular level of cyclic AMP (cAMP, red circle), which binds to the regulatory subunit of PKA, thereby releasing and activating the catalytic subunits. Arrows indicate activation and barred lines indicate inhibition. **(b) ***BCY1 *expression. **(c,d) **The average expression of PKA-responsive genes. The average is over (c) 161 PKA-induced and (d) 314 PKA-repressed genes (identified by [35]). Each expression pattern is accompanied by a colored bar indicating the log_2 _fold change.

In vegetative cells, much of the glucose effect is mediated through cyclic AMP-dependent protein kinases (PKAs), which are activated upon the addition of glucose [38,39] (Figure 7a). In particular, active PKAs block the onset of sporulation [40]. Notably, *BCY1*, which codes for the negative regulatory subunit of the PKAs, was specifically induced in committed cells (Figure 7b). To gain further insight into the regulation of the PKA pathway, we analyzed the expression pattern of genes whose glucose-dependent induction in vegetative cells is mediated by the PKA pathway. Indeed, these genes altered their expression upon the addition of YPD to early meiotic cells, but not when YPD was added to cells that have progressed beyond the commitment point (Figure 7c,d). Taken together, it appears that the addition of glucose-containing growth-medium to committed cells leads to the repression, rather than activation, of the PKA pathway.

## Discussion

Differentiation processes proceed through a sequence of events involving coordinate modulations of morphology and gene expression. In many cases, once the process has passed a certain stage it becomes determined and loses its dependence on environmental signals. This transition is termed commitment. Mechanisms for achieving commitment can be classified into two broad classes. First, a passive mechanism might be imagined, whereby the process becomes effectively insulated from the external signals. Alternatively, commitment might require an active modulation of the signaling apparatus, which senses the external signal, but interprets it in a specific manner that optimizes the continuation of the process in changing conditions.

Differentiating cells could be insulated from the environment through the inhibition of essential sensory receptors, such as the receptors for glucose or nitrogen in the case of yeast sporulation. Effective insulation of the differentiation process could also be achieved through positive feedback loops that would render the process self-sustaining. Such positive feedbacks are ubiquitous in differentiation cascades. In the case of the *Xenopus *oocyte at least this positive feedback loop has been shown to be important for commitment [41]. Also in the case of yeast sporulation, the capacity of Ndt80 to autoactivate its own gene expression could in principle implement such a feedback.

Our gene-expression analysis ruled out the possibility that commitment to sporulation is achieved through a passive mechanism. Cells responded to nutrients at all stages of the sporulation process (see Additional data file 5 for a matlab program that enables the reader to view our data). In fact, within 5 minutes of the addition of rich medium, we observed a change in the expression pattern of over 1,000 genes (around 15% of the yeast genome). Importantly, this large-scale transcription response was dramatically different in committed versus non-committed cells, and was highly specific to the type of medium added. In particular, the response seen on addition of YPD (containing both glucose and nitrogen) was distinct from that observed on the addition of YPA (containing nitrogen and acetate, but not glucose) or on the addition of glucose alone. This indicates that the reduction in mRNA is a result of combinatorial regulation. The machinery of committed cells responds to the combination of glucose and nitrogen signals. This behavior suggests that active modulation of the response to external signals takes place. This modulation may play a central role in enabling the continuation of the sporulation process after the addition of nutrients.

How do the cells achieve commitment to meiosis? The continuation of sporulation in rich media poses two complementary challenges. First, cells need to overcome mitogenic signals, which in vegetative cells facilitate the mitotic program. Second, cells need to ensure the continuation of the sporulation process itself. Our data suggest that these two aspects are goverened by complementary molecular mechanisms.

The mitogenic effects of glucose are mediated to a large extent by the PKA pathway [42,43]. This pathway represents a central junction in the choice between the meiotic and the mitotic programs: Entry into the mitotic cell cycle requires high activity of this pathway, whereas entry into meiosis requires low PKA activity [40,44-48]. Interestingly, our data suggest that committed cells respond to glucose not by activating the PKA pathway, but rather by inhibiting it through the induction of *BCY1*, the gene coding for the negative regulatory unit of the PKAs. This inhibition is probably required in order to overcome mitogenic signals, to ensure the continuation of the meiotic program upon the addition of nutrients.

The addition of either YPD or glucose to committed cells failed to induce the typical set of glucose-responsive genes. In contrast, the addition of YPD (but not of glucose alone) had a dramatic effect on the sporulation cascade itself. In fact, the vast majority of genes that are induced during sporulation in SPM were immediately repressed. This repression did not reflect the lack of functional requirement, as many of the repressed genes had a well defined role in the two meiotic divisions or in the formation of the spore wall. It may be that the proteins encoded by these genes are already synthesized at the time of commitment in sufficient amounts to complete the meiotic division. Alternatively, it is possible that the downregulation of at least some of the mid-sporulation genes is in fact required to prevent a shift back to the mitotic cell cycle upon the addition of nutrients, thus augmenting the cells' commitment. Further work is needed in order to better understand the nature of this downregulation and its potential contribution to the commitment process.

Whereas the transcription program characterizing sporulation in sporulation medium (SPM) was practically eliminated in YPD, a group of around 60 late-sporulation genes was also induced in YPD, in a temporal manner that appears to be linked to the progression of the sporulation process. Surprisingly, the vast majority of these genes do not have a known role in sporulation. Rather, this group included a majority of stress-related genes, which are also induced in response to a variety of environmental stresses [36]. The timely induction of these genes may indicate the generation of some stress-related signal. Such stress could be initiated, for example, by the onset of spore-wall deposition, similarly to the stress signal that ensues after the formation of a mating projection (a shmoo) during the mating process [49]. In the case of mating, this stress signal is propagated through the protein kinase C (PKC) pathway to induce the second wave of gene expression. It is tempting to propose that here also, an analogous stress signal may be associated with the commitment, by marking the initiation of a particular developmental stage (for example, spore-wall formation) and triggering subsequent processes required for the completion of sporulation. It is of interest that the formation of the pro-spore membrane is initiated on the SPBs that were implicated in commitment [15]. Such a proposal may also be consistent with a potential role for *SPO14 *in both the formation of a spore membrane and the commitment to sporulation [50]. This proposition awaits further experimental validation.

In conclusion, previous theoretical and experimental work has shown that bistability, generated through positive feedback loops, can render a process irreversible by effectively isolating it from external signals [41,51-53]. In contrast, sporulating cells modulate their transcription program in response to changing conditions, proceeding through different alternative paths. This alternative strategy is likely to be beneficial when a differentiating cell is challenged not only with the removal of the initiating signal but also with competing signals that could potentially interfere with structural or morphological processes, or could signal alternative fates. The experimental design presented here could be applied to distinguish between these alternatives. Further studies are required to examine which of the two strategies is more prominent during cellular differentiation.

## Materials and methods

### Yeast strains and plasmids

All strains used for this study are of the SK1 genetic background and are listed in Additional data file 3. The *pSPS4-3HA-NDT80 *strain (GF18) was constructed by a one-step PCR-based replacement method [54] using the plasmid pFA6a-pSPS4-3HA-KanMX6. The latter was constructed by replacing the *GAL1 *promoter fragment in pFA6a-pGAL-3HA-KanMX6 with a 1 kb PCR fragment of the *SPS4 *promoter. Transformed haploids NKY1712 were mated with strain NE30. Integration to the correct site was verified by PCR. Diploids were selected on minimally supplemented yeast synthetic dextrose (SD) plates and were checked for sporulation efficiency on sporulation plates.

### Sporulation, return to growth, and germination experiments

The compositions of all media are given in Additional data file 3. Cells (NKY1551, JPY214, or GF18) were grown to saturation in YPD for 24 hours, diluted into YPA and grown overnight. The cells were then washed twice in sterile water and resuspended in SPM to initiate the sporulation process. At different stages of the sporulation process, cells were centrifuged and resuspended in rich medium. At each time point, 5 × 10^8 ^cells were frozen in liquid nitrogen. To monitor the meiotic divisions, 2 ml of cells were kept for staining with 4,6-diamidino-2-phenylindole (DAPI). To monitor recombination frequencies at the *HIS4 *locus, cells were plated on both YPD and -His plates, and colonies were counted after three days incubation at 30°C. All liquid cultures were grown at 30°C with constant shaking. Sporulation efficiency was determined by counting asci in the overnight SPM culture. For germination experiments, cells (DS1) were grown to saturation in YPD for 24 hours and plated on sporulation plates. Three-day-old asci were harvested and suspended in YPD at 30°C to initiate spore germination.

### DAPI staining

Cells (2 ml) were centrifuged for 30 seconds, fixed in 0.25 mM Tris, 70% ethanol and kept at 4°C. Before staining, cells were washed in 1× PBS solution (137 mM NaCl, 2.7 mM KCl, 8 mM Na_2_HPO_4_, 2 mM KH_2_PO_4_), sonicated for 5 seconds and centrifuged. One microliter DAPI (at a concentration of 25 μg/ml) was added to the pellet and incubated in the dark for 10 minutes at room temperature. The number of nuclei per cell was counted using a fluorescence microscope. Around 300 cells were analyzed for each sample.

### Defining the timing of commitment

To define the time of commitment, we transferred sporulating cells from SPM to a solution containing 4% glucose at various times (Figure 1f). Glucose is a potent inhibitor of sporulation initiation, but glucose alone does not support growth or germination. For each time point, we calculated the fraction of cells that became spores in over night glucose cultures. We used glucose rather then YPD in this experiment because glucose does not support growth of vegetative cells or germination of mature spores. This allows for a precise quantification of the fraction of spores after overnight incubation. In contrast, on transfer to YPD, the uncommitted cells initiate growth and cell division, and quickly take over the population. Moreover, as the culture is not fully synchronized, some spores may start to germinate early, while others have not yet become spores. We normalized for this effect when looking at the completion of meiotic division II among DAPI-stained cells (Figure 1e), but such normalization is difficult for the longer times corresponding to spore maturation. Thus, although we clearly saw the formation of mature spores in cells transferred to YPD after five hours in SPM, it was difficult to use this fraction as a quantitative measure for commitment.

### RNA extraction and labeling

Total RNA was extracted using the RNeasy Midi Kit (Qiagen, Valencia, USA) and reverse transcribed using M-MLV reverse transcriptase RNase H Minus (Promega, Madison, USA). cDNA products were labeled with Cy3 and Cy5 by the indirect amino-allyl method [55] with minor modifications. Total RNA (20 μg) was combined with 8 μg of oligo(dT) 12- to 18-mer (Amersham Pharmacia Biotech, Little Chalfont, UK) in a final volume of 26 μl and heated to 70°C for 10 minutes to denature RNA tertiary structures. The mixture was then transferred to 45°C to anneal the primers. A preheated (50°C) 12 μl mixture containing reaction buffer (Promega), 2.5 mM MgCl_2_, 0.5 mM dATP, dCTP and dGTP, 0.1 mM dTTP, 0.4 mM amino-allyl dUTP (Ambion, Austin, USA), RNase H Minus and 40 Units RNasin (Promega) was added to the RNA, and the reaction mixture was incubated for 2 hours at 45°C with 400 units of Superscript II (Gibco, Carlsbad, USA). An additional 400 units of the enzyme was applied to the reaction mixture, which was incubated for another 2 hours. After incubation, RNA was hydrolyzed by adding 10 μl of 1 M NaOH and incubating at 65°C for 30 minutes. The reaction mixture was neutralized by titration with 1 M Tris-HCl pH 7.5, and DNA was precipitated with ethanol, resuspended in 10 μl 0.1 M sodium carbonate buffer pH 9.3. Cy3 and Cy5 (Amersham) were dissolved in 10 μl DMSO. Cy5 or Cy3 (1.25 μl) was added to the sample and incubated at 25°C for 1 hour. Labeled cDNA was purified by Strataprep PCR purification kit (Stratagene, La Jolla, USA) according to the manufacturer's instructions. Dye incorporation was measured using a spectrophotometer. Reference RNA for all microarrays in this work was from YPA-grown cells, just before the transfer to SPM.

### Microarray hybridization

For each hybridization, cDNA samples were labeled with Cy3 and Cy5 and combined with blockers: 5 μg herring sperm (Promega), 5 μg tRNA (Gibco) and 17.5 μg poly(A) (poly(A) oligonucleotides were synthesized at mixed lengths of 40, 50, and 60 adenine residues). The labeled cDNAs were concentrated to 40 μl using Microcon (Millipore, Bedford, USA) and 40 μl of 2× hybridization solution (10× SSC, 50% formamide, 0.2% SDS) was added. Microarrays containing all yeast open reading frames (ORFs) were prehybridized by incubating at 42°C for 45 minutes in a solution containing 1% BSA, 25% formamide, 5× SSC and 0.1% SDS. The slides were washed in sterile water and dried by centrifugation (3 minutes, 2,000 rpm). The labeled samples were boiled for 5 minutes, centrifuged for 1 minute, hybridized on the slide and placed in a hybridization chamber (Corning, Corning, USA) for overnight incubation at 42°C. The slides were then washed for 5 minutes at 42°C with a solution containing 2× SSC and 0.1% SDS. An additional wash was performed at room temperature with a solution containing 0.1× SSC and 0.1% SDS, followed by three additional washes at room temperature in 0.1× SSC solution. For most experiments, we used arrays purchased from the University Health Network Microarray Center, Toronto, Canada, where PCR products are printed. For the YPA experiment (35 arrays) we used slides obtained from F. Holstege (UMC Utrecht, the Netherlands), where genes are represented by 70-base oligonucleotides [56]. Control experiments have been performed to check that differences in the data were not caused by differences in the arrays (these data have been deposited in the Gene Expression Omnibus (GEO) database run by the National Center for Biotechnology Information (NCBI) [57-59] and are accessible through GEO Series accession number GSE3820).

### Scanning and quantification

Images were obtained using ScanArray 4000 (Packard BioScience, Meriden, USA). Image analysis was performed using QuantArray version 3 software (PerkinElmer Life Sciences, Boston, USA), with spots quantified using the adaptive method. Low-quality spots were discarded following detailed visual inspection. The data were then transformed into log_2 _ratios, and normalized by subtracting the median. Values of replicate spots on the slides were averaged (see Additional data file 1 for MIAME data). The data discussed in this publication have been deposited in GEO [59] and are accessible through GEO Series accession numbers: GSE3814, GSE3815, GSE3816, GSE3817, GSE3818, GSE3819 and GSE3820 [57,58] (see also Additional data file 4).

### Calculating the correlation coefficients with results of previous experiments

To compare our sporulation time course with previous experiments, the different time courses were first aligned such that similar time points corresponded to each other (progression into sporulation) [60,61]. In each pairwise comparison, one experiment (A) was held constant and the other (B) was transformed to best match the time points of A. The transformation was done in two steps: first, smoothing splines were used to add intermediate time points to B (by creating a continuous representation of B, and re-sampling it at a higher frequency) [60]; second, we searched for the time points of B that maximizes the correlations between each time point in A and the corresponding time point in B over all genes. We only considered the time points of A, or transformations of the kind T' = T*(1 + k1) + k2, where T are the time points of A, k1 is a 'stretching' parameter, and k2 is a 'shifting' parameter. After transforming B, we calculated the average correlation between corresponding time points in A and B, over all genes (the same measure that was used to determine the optimal transformation). For each pairwise comparison of A and B this procedure was done twice, once when starting with A and once when starting with B. The average correlation is given.

### Comparison of the responses of sporulating cells, vegetative cells and spores to glucose

We included in this analysis genes whose expression was altered by the addition of glucose to vegetative cells. To reduce noise, we filtered out genes whose expression was not altered by YPD in our experiment (transfer of sporulating cells to YPD). We ended with 936 genes (see Additional data file 3). For the vegetative cell data, we calculated the average expression profile over the three given time points (20, 40, and 60 minutes), and considered a given gene to be down- or upregulated if its average expression change exceeded twofold. For the sporulation data, we considered the YPD expression relative to that observed during sporulation, and calculated the average expression change observed at 20 and 40 minutes after the transfer. For spores that completed the sporulation process, we calculated the average expression response observed at 15 and 30 minutes following the addition of YPD. Correlations were calculated between these average profiles. The correlation matrix of the sporulation data was clustered [62] (separately for glucose-induced and glucose-repressed genes). Only genes belonging to homogeneous clusters were kept.

### Identification of gene groups

A few genes with a common expression pattern were chosen as template genes. To find more genes with the same expression pattern we calculated the correlation of each gene in the genome with each of the template genes. The genes were ordered according to their correlation with the template gene. Genes with the highest correlation were defined as genes in the group. For each template gene the threshold was determined by inspection of the expression pattern of the genes obtained. The top genes with the same expression pattern were chosen. The qualitative results of our study do not depend on these thresholds.

### Utilization of Gene Ontology annotations for identification of polarized gene expression during sporulation and RTG

For each of the chosen Gene Ontology (GO) [28] groups we filtered out genes in the group which their expression did not change (we kept only genes whose expression changed more than twofold in one array at least). We then obtained a matrix of the expression of the selected genes in the following conditions: the sporulation time points and transfers to YPD at early times (2, 3, and 4 hours). The correlation between any two genes in this matrix was computed to give the gene-gene correlation matrix. We then clustered [62] this gene-gene correlation matrix and chose homogeneous clusters.

## Additional data files

The following additional data files are included with this article: Additional data file 1 includes minimum information about a microarray experiment (MIAME). Additional data file 2 includes supplementary figures S1 to S3. Figure S1 shows polarized expression during sporulation and RTG. Figure S2 shows the expression pattern of *IME2 *and *SMK1*. Figure S3 shows a matrix of pairwise correlations that exemplifies the general metabolic response to glucose. Additional data file 3 includes supplemental tables S1 to S17. Table S1 includes a list of early sporulation genes. Table S2 includes a complete list of genes used in Figure 3d of the paper. Table S3 lists examples of genes that according to their expression during RTG are regulated by the mitotic cell cycle. Table S4 lists genes that are induced during RTG (20 minutes after the transfer). Table S5 lists genes that are induced during RTG (immediately after the transfer). Table S6 lists homologous genes which are induced during sporulation or RTG. Table S7 lists middle sporulation genes that are repressed upon transfer to YPD. Table S8 lists insulated middle sporulation genes. Table S9 lists the 936 genes used for Figure 6a. Table S10 lists rRNA-processing genes. Table S11 lists gluconeogenesis genes. Table S12 lists genes that encode ribosomal proteins. Table S13 lists genes induced in a time-dependent manner. Table S14 lists genes that are induced in response to YPD in committed cells. Table S15 lists genes that are repressed in response to YPD in committed cells. Table S16 includes a list of the yeast strains used in the present study. Table S17 includes the composition of the media used in the present study. Additional data file 4 includes the normalized data of the present study (in log_2 _ratios). Addtional file 5 is a zip file containing a matlab program that enables to view the expression data discussed in this article. It contains also a help file.

## Supplementary Material

Additional File 1 A checklist containing minimum information about a microarray experiment. (MIAME) Click here for file

Additional File 2 Figure S1 shows polarized expression during sporulation and RTG. Figure S2 shows the expression pattern of *IME2 *and *SMK1*. Figure S3 shows a matrix of pairwise correlations that exemplifies the general metabolic response to glucose. Click here for file

Additional File 3 Table S1 includes a list of early sporulation genes. Table S2 includes a complete list of genes used in Figure 3d of the paper. Table S3 lists examples of genes that according to their expression during RTG are regulated by the mitotic cell cycle. Table S4 lists genes that are induced during RTG (20 min after the transfer). Table S5 lists genes that are induced during RTG (immediately after the transfer). Table S6 lists homologous genes which are induced during sporulation or RTG. Table S7 lists middle sporulation genes that are repressed upon transfer to YPD. Table S8 lists insulated middle sporulation genes. Table S9 lists the 936 genes used for Figure 6a. Table S10 lists rRNA-processing genes. Table S11 lists gluconeogenesis genes. Table S12 lists genes that encode ribosomal proteins. Table S13 lists genes induced in a time-dependent manner. Table S14 lists genes that are induced in response to YPD in committed cells. Table S15 lists genes that are repressed in response to YPD in committed cells. Table S16 includes a list of the yeast strains used in the present study. Table S17 includes the composition of the media used in the present study. Click here for file

Additional File 4 Normalized data of the present study (in log_2 _ratios) Click here for file

Additional File 5 A matlab program that enables the expression data discussed in this article to be viewed. Also contains a help file: 'ViewModules help.pdf' Click here for file
