# Supplementary material for: Modulation of the transcription regulatory program in yeast cells committed to sporulation
Source: Genome Biol. 2006 Mar 8;7(3):R20. doi: 10.1186/gb-2006-7-3-r20 (PMC1557749; doi:10.1186/gb-2006-7-3-r20)
Supplement: Additional File 1 — A checklist containing minimum information about a microarray experiment. (MIAME) [file gb-2006-7-3-r20-S1.doc]

## The MIAME checklist for the paper:

## ' Modulation of the transcription regulatory program in yeast cells committed to sporulation'

**1. Experimental design:**

The paper reports six experiments, studying the response of sporulating cells to rich media. In the following we provide a detailed account for each experiment:

*a.* ***Transfer of sporulating wild type cells to YPD***

• Type of experiment: Time-course.

- Experimental factors: wild type SK1 cells (NKY1551) grown to saturation in YPD (2% yeast extract, 4% bactopeptone, 4% glucose) for 24 hours, diluted into YPA (1% yeast extract, 2% bactopeptone 1% Potassium Acetate) and grown overnight. Part of that culture was used as reference for all hybridizations preformed in this study. The cells were then washed twice with sterile water and resuspended in SPM media (0.3% Potassium Acetate and 0.02% Rafinose) to initiate the sporulation process. At different stages of the sporulation process (2, 3, 4, 5, 6, 7, 8, 9 and 10 hours) RNA was extracted for microarray hybridizations. At each hour, part of the culture was centrifuged and resuspended in YPD. Each YPD culture was monitored immediately after the transfer, after 20 and 40 minutes.
- Arrays used for that experiment: Microarrays printed with PCR products of all yeast genes (in duplicates), purchased from the Microarray Centre, University Health Network, Ontario (2 hours and 3 hours of sporulation using Yeast 6.4K1 v2 and the rest with Yeast 6.4Kv2**)**.
- Number of hybridizations performed: nine hybridizations along the sporulation; three hybridizations for each transfer to YPD. Therefore a total of thirty six hybridizations was preformed for this experiment (one hybridization for each time point).
- Reference: Acetate growing cells, before the transfer to sporulation media.

1. ***Transfer of sporulating wild type cells to YPA***

• Type of experiment: Time-course.

- Experimental factors: wild type SK1 cells (NKY1551) grown to saturation in YPD (2% yeast extract, 4% bactopeptone, 4% glucose) for 24 hours, diluted into YPA (1% yeast extract, 2% bactopeptone 1% Potassium Acetate) and grown overnight. The cells were then washed twice with sterile water and resuspended in SPM media (0.3% Potassium Acetate and 0.02% Rafinose) to initiate the sporulation process. At different stages of the sporulation process (2, 3, 4, 5, 6, 7, 8, 9 and 10 hours) RNA was extracted for microarray hybridizations. At each hour, part of the culture was centrifuged and resuspended in YPA. Each YPA culture was monitored immediately after the transfer, after 20 and 40 minutes.

Arrays used for that experiment: Microarrays printed with oligonucleotides corresponding to all yeast genes (in duplicates), purchased from the Microarray Facility, University Medical Center Utrecht (version 16k-1.2).

- Number of hybridizations performed: eight hybridizations along the sporulation (as a result of technical problems we did not hybridize the 10 hour time point); three hybridizations for each transfer to YPA (as a result of technical problems we did not hybridize the sample of 20 minutes after transfer at 9 hours). Therefore a total of thirty four hybridizations was preformed for this experiment (one hybridization for each time point).
- Reference: Acetate growing cells, before the transfer to sporulation media (obtained in experiment a: Transfer of sporulating wild type cells to YPD).

*c.* ***Transfer of sporulating wild type cells to glucose solution***

• Type of experiment: Time-course.

- Experimental factors: wild type SK1 cells (NKY1551) grown to saturation in YPD (2% yeast extract, 4% bactopeptone, 4% glucose) for 24 hours, diluted into YPA (1% yeast extract, 2% bactopeptone 1% Potassium Acetate) and grown overnight. The cells were then washed twice with sterile water and resuspended in SPM media (0.3% Potassium Acetate and 0.02% Rafinose) to initiate the sporulation process. At different stages of the sporulation process (2, 3, 4, 5, 6, 7, 8, 9 and 10 hours) RNA was extracted for microarray hybridizations. At each hour, part of the culture was centrifuged and resuspended in glucose solution (4%). Each glucose culture was monitored immediately after the transfer, after 20 and 40 minutes.
- Arrays used for that experiment: Microarrays printed with PCR products of all yeast genes (in duplicates), purchased from the Microarray Centre, University Health Network, Ontario (version Yeast 6.4Kv4).
- Number of hybridizations performed: nine hybridizations along the sporulation; three hybridizations for each transfer to YPA. Therefore a total of thirty six hybridizations was preformed for this experiment (one hybridization for each time point).
- Reference: Acetate growing cells, before the transfer to sporulation media (obtained in experiment a: Transfer of sporulating wild type cells to YPD).

*d.* ***Transfer of sporulating SPS4-NDT80 cells to YPD***

• Type of experiment: Time-course.

- Experimental factors SK1 cells, in which NDT80 is expressed under the promoter of SPS4 (GF18), were grown to saturation in YPD (2% yeast extract, 4% bactopeptone, 4% glucose) for 24 hours, diluted into YPA (1% yeast extract, 2% bactopeptone 1% Potassium Acetate) and grown overnight. The cells were then washed twice with sterile water and resuspended in SPM media (0.3% Potassium Acetate and 0.02% Rafinose) to initiate the sporulation process. At 5.5 hours of the sporulation process, cells were kept for RNA extraction. Part of the culture was centrifuged and resuspended in YPD, and was monitored immediately, 20 and 40 minutes after the transfer.
- Arrays used for that experiment: Microarrays printed with PCR products of all yeast genes (in duplicates), purchased from the Microarray Centre, University Health Network, Ontario (version Yeast 6.4Kv4).
- Number of hybridizations performed: four hybridizations (one during the sporulation and three after the transfer to YPD).
- Reference: Acetate wild type growing cells, before the transfer to sporulation media (obtained in experiment a: Transfer of sporulating wild type cells to YPD).

*e.* ***Transfer of sporulating Δsum1 cells to YPD***

• Type of experiment: Time-course.

- Experimental factors: *Δsum1* SK1 cells (JPY214) were grown to saturation in YPD (2% yeast extract, 4% bactopeptone, 4% glucose) for 24 hours, diluted into YPA (1% yeast extract, 2% bactopeptone 1% Potassium Acetate) and grown overnight. The cells were then washed twice with sterile water and resuspended in SPM media (0.3% Potassium Acetate and 0.02% Rafinose) to initiate the sporulation process. At 6 hours of the sporulation process, cells were kept for RNA extraction. Part of the culture was centrifuged and resuspended in YPD, and was monitored 20 and 40 minutes after the transfer.
- Arrays used for that experiment: Microarrays printed with PCR products of all yeast genes (in duplicates), purchased from the Microarray Centre, University Health Network, Ontario (version Yeast 6.4Kv4).
- Number of hybridizations performed: three hybridizations (one during the sporulation and two after the transfer to YPD).
- Reference: Acetate wild type growing cells, before the transfer to sporulation media (obtained in experiment a: Transfer of sporulating wild type cells to YPD).

*f.* ***Transfer of wild type spores to YPD***

• Type of experiment: Time-course.

- Experimental factors: Wild type SK1 cells (DS1) were grown to saturation in YPD (2% yeast extract, 4% bactopeptone, 4% glucose) for 24 hours and plated on sporulation plates (0.25% yeast extract, 1.5% Potassium acetate, 0.05% glucose supplemented with all amino acids). 3 days old asci were harvested and suspended in YPD at 30°C to initiate spore germination. RNA for microarray hybridization was extracted at the indicated times.
- Arrays used for that experiment: Microarrays printed with PCR products of all yeast genes (in duplicates), purchased from the Microarray Centre, University Health Network, Ontario (version Yeast 6.4Kv3.48 for spores and 30 minutes; version Yeast 6.4Kv4 for 15 minutes).
- Number of hybridizations performed: three hybridizations (one of spores and two after – 15 and 30 minutes after the transfer to YPD).
- Reference: The reference for microarray hybridization was extracted from logarithmic growing haploid cells. Mata and Matα cells were grown separately in YPD. RNA was extracted and pooled together.

**2. Samples used, extract preparation and labelling:**

- Origin of biological samples: The *S. cerevisiae* strains used are detailed in strain list in the file named "strain list".
- Manipulation of biological samples: Cells were pelleted by centrifugation and flash frozen in liquid nitrogen.
- Preparation of RNA extract for hybridization: 5x10^8^ cells were flash frozen in liquid Nitrogen. Total RNA was extracted using RNeasy Midi kit (QIAGEN), with slight modification of the provider protocol. RNA concentration was determined using a spectrophotometer.
- Labeling protocol: cDNA was synthesized using M-MLV Reverse Transcriptase RNase H Minus (Promega) and labeled with Cy3 and Cy5 by the indirect amino-allyl method as described below. Total RNA (20 µg) was mixed with 4 µg oligo dT_12-18_ (Amersham) in a volume of 26 μM and incubated at 70°C for 10 minutes. Reaction buffer (Promega), 2.5mM MgCl_2_, 0.5 mM of dATP, dCTP and dGTP, 0.1mM dTTP, 0.4mM amino-allyl dUTP (Ambion) and 400U M-MLV Reverse Transcriptase RNase H Minus, 40 Units RNasin (Promega) were added and the reaction was incubated at 45°C for 2 hours. Additional 400U M-MLV Reverse Transcriptase RNase H Minus was added and incubated for another 2 hours. The reaction was terminated by incubation at 70°C for 15 minutes. The RNA was degraded by adding 10 μM 1N NaOH and incubation at 65°C for 30 minutes. The sample was neutralized by titrating with 1M HCl. cDNA was purified by ethanol precipitation, resuspended in 10 µl 0.1M Sodium Carbonate buffer pH 9.3. Experimental RNA samples were labeled with Cy5 and the reference RNA samples, were labeled with Cy3. Cy3 and Cy5 (Amersham) were dissolved in 10 μM DMSO. 1.25 μM of Cy5 or Cy3 wee added to experimental or reference sample, respectively, and incubated at 25°C for 1 hour. Labeled cDNA was purified by Strataprep PCR purification kit (Stratagene) according to the manufactures instructions. Dye incorporation was measured using a spectrophotometer.

3**. Hybridization procedures and parameters:**

- For each hybridization, labeled Cy3 and Cy5 were combined together with the following blockers: 5μg Herring sperm (Promega), 5 μg tRNA (Gibco) and 17.5μg Poly A (Synthesized oligos with the length of 40, 50 and 60), and concentrated to 25μl using Microcon (Millipore). 25 μ of hybridization x2 solution (10x SSC, 50% formamide and 0.2% SDS) was added.
- Microarrays containing all yeast ORFs (purchased from the Microarray Centre, University Health Network, Ontario) were pre-hybridized by incubating in a solution containing 1% BSA, 25% formamide, 5x SSC and 0.1% SDS at 42°C for 45 minutes. The slides were washed with DDW and dried by centrifugation (3 minutes, 2000 rpm).
- The labeled sample with the blockers, was boiled for 5 minutes, centrifuged for 1 minute and hybridized on the slide. The slides were placed in hybridization chamber (Corning) and incubated over night at 42°C. The slides were washed for 5 minutes at 42°C with a solution containing 2x SSC and 0.1% SDS. Additional wash was performed at room temperature with a solution containing 0.1x SSC and 0.1% SDS, followed by 3 additional washes at room temperature in 0.1x SSC solution.

4**. Measurements and Specifications**

- Type of scanning and software used: The Microarrays were scanned with ScanArray 4000 (Packard BioScience) according to the manufactures instruction.
- Type of image analysis software used: The images were quantified with QuantArray version 3 software (Packard BioScience, Perkin-Elmer Life Sciences, Boston) using the adaptive method.
- Data selection: For each array, bad spots were omitted from the analysis using home-made data analysis program which combined automatic detection of suspicious spots with visual inspection. The program combines the quality information provided by the quantrarray software, with correlation analysis of duplicated spots on the array. The programs are in a GUI interface written in Matlab, and are available upon request.
- Transformation procedure:
  - For each spot, the Quantarray program outputs the level of signal intensity and background fluorescence. As a first step, we re-defined the spot intensity by subtracting the spot-specific background.
  - For each channel (Cy3 or Cy5) we then calculated the value of background signal, defined by the mean (intensity-background) of the controlled spots printed with 3x SSC buffer (116 spots) All intensity values below this background level were reset to this background level.
  - The data for each spot was then transformed into a log2-ratios:
  - The data was normalized by subtracting from each log_2_ratio the median. Values of duplicates were averaged

**5. Array Design**

In the present study we used two kinds of arrays:

I. Microarrays printed with PCR products of all yeast genes (in duplicates), purchased from the Microarray Centre, University Health Network, Ontario (<http://www.microarrays.ca/>).

- General array design: Glass arrays spotted with PCR products. Each gene (6218 genes) in the yeast genome was represented by two adjacent points on the array. In addition the array included 116 spots of 3xSSC buffer and 248 spots of an Arabidopsis gene, so that all together there were 12,800 spots on the array.
- Identity of spots on the array: the spot location on the array can be seen in the description files (for Yeast 6.4K1 v2: QA_Y6.4k1_v2.uni ;for 6.4Kv2: y6.4k2.qa; for 6.4Kv4: Y6.4k4.qa; for each experiment the version of the slides is indicated in the Experimental Design section).

II. Microarrays printed with oligonucleotides corresponding to all yeast genes (in duplicates), purchased from the Microarray Facility, University Medical Center Utrecht.

- General array design: Glass arrays spotted with oligonucleotides (70 nucleotides in length). Each gene (6329 genes) in the yeast genome was represented by two points on the array. In addition the array included 2894 control spots (3xSSC buffer, empty and other external controls), so that all together there were 15,552 spots on the array. The slides are printed on Corning UltraGAPS slides.
- Identity of spots on the array: the spot location on the array can be seen in the description file, UMCY-16k-1.2.xls.
