# Supplementary material for: Modulation of the transcription regulatory program in yeast cells committed to sporulation
Source: Genome Biol. 2006 Mar 8;7(3):R20. doi: 10.1186/gb-2006-7-3-r20 (PMC1557749; doi:10.1186/gb-2006-7-3-r20)
Supplement: Additional File 2 — Figure S1 shows polarized expression during sporulation and RTG. Figure S2 shows the expression pattern of IME2 and SMK1. Figure S3 shows a matrix of pairwise correlations that exemplifies the general metabolic response to glucose. [file gb-2006-7-3-r20-S2.pdf]

Supplementary Figure S1: Polarized expression during sporulation and Return to Growth

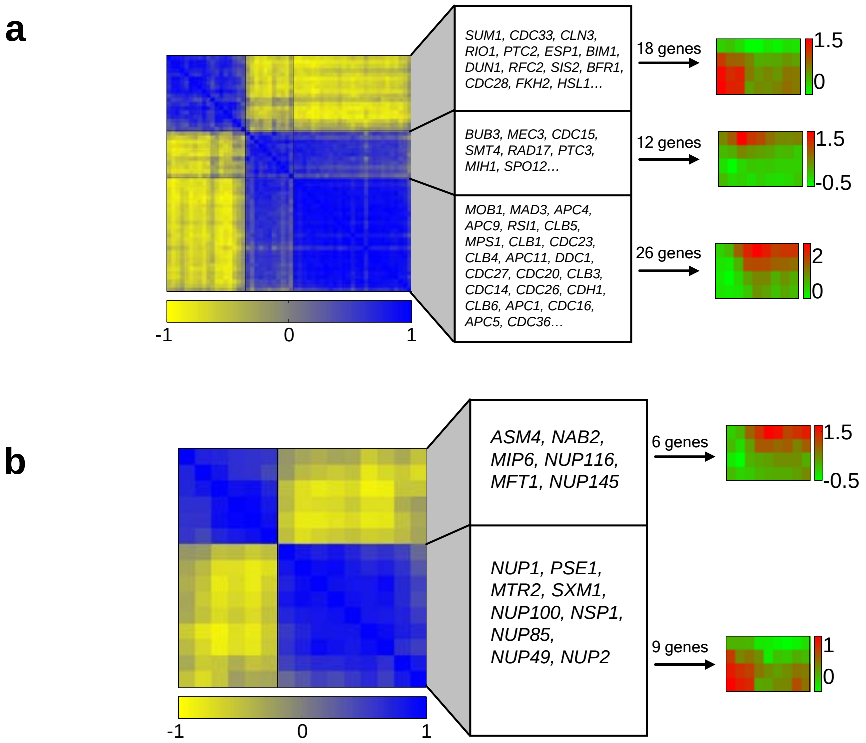

Polarized expression of genes associated with regulation of cell cycle (a) or mRNA export from the nucleus (b). Shown is the matrix of pair-wise correlation coefficients between genes assigned to the corresponding Gene Ontology (GO) groups (see Materials and Methods), clustered according to similarity in correlation patterns. The average expression pattern of genes in each cluster is shown on the right.

The complete list of genes used in Supplementary Figure 1A:

Figure A-cluster 1	Figure A-cluster 2	Figure A-cluster 3
SUM1	*BUB3*	*MOB1*
YGR211W	*MEC3*	*MAD3*
CDC33	*CDC15*	*APC4*
YLR238W	*YGL240W*	*APC9*
YNL078W	*SMT4*	*RSI1*
CLN3	*YIR025W*	*CLB5*
YGR245C	*RAD17*	*MPS1*
RIO1	*PTC3*	*YDR499W*
PTC2	*MIH1*	*CLB1*
ESP1	*YNL127W*	*CDC23*
BIM1	*YBR274W*	*CLB4*
DUN1	*SPO12*	*APC11*
RFC2		*YDR200C*
SIS2		*DDC1*
BFR1		*CDC27*
CDC28		*YDR260C*
FKH2		*CDC20*
HSL1		*CLB3*
		CDC14
		CDC26
		CDH1
		CLB6
		APC1
		CDC16
		APC5
		CDC36

Supplementary Figure S2: Expression pattern of *IME2* and *SMK1*

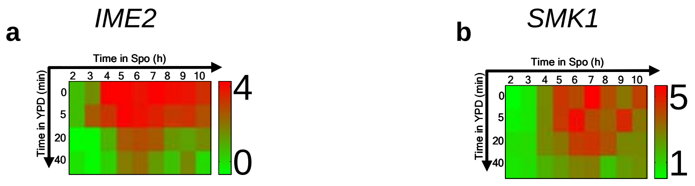

The expression pattern of the *IME2* (a) and *SMK1* (b).

Supplementary Figure S3: The general metabolic response to glucose.

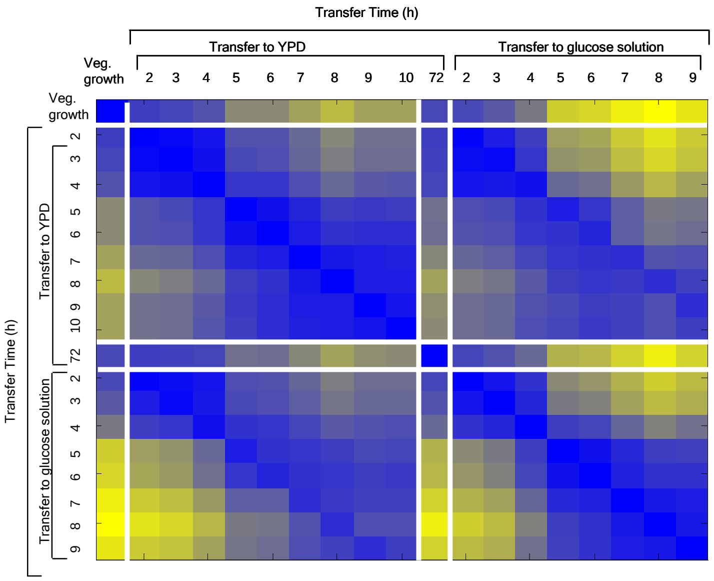

Shown is the matrix of pair-wise correlations, describing the similarity in the response of cells transferred to YPD (marked transfer to YPD) or glucose solution (marked transfer to glucose solution) at different stages of the process. We compared these responses also with the response of vegetative cells to glucose [35], marked veg. growth, and the response to mature spores (sporulated for 72 hours; marked 72) to YPD. Correlations were calculated based on 936 genes whose expression was induced upon the addition of glucose to vegetative cells or sporulating cells (see additional file 3: Supplementary Table S9 for the list of genes).
