# Supplementary material for: Modulation of the transcription regulatory program in yeast cells committed to sporulation
Source: Genome Biol. 2006 Mar 8;7(3):R20. doi: 10.1186/gb-2006-7-3-r20 (PMC1557749; doi:10.1186/gb-2006-7-3-r20)
Supplement: Additional File 3 — Table S1 includes a list of early sporulation genes. Table S2 includes a complete list of genes used in Figure 3d of the paper. Table S3 lists examples of genes that according to their expression during RTG are regulated by the mitotic cell cycle. Table S4 lists genes that are induced during RTG (20 min after the transfer). Table S5 lists genes that are induced during RTG (immediately after the transfer). Table S6 lists homologous genes which are induced during sporulation or RTG. Table S7 lists middle sporulation genes that are repressed upon transfer to YPD. Table S8 lists insulated middle sporulation genes. Table S9 lists the 936 genes used for Figure 6a. Table S10 lists rRNA-processing genes. Table S11 lists gluconeogenesis genes. Table S12 lists genes that encode ribosomal proteins. Table S13 lists genes induced in a time-dependent manner. Table S14 lists genes that are induced in response to YPD in committed cells. Table S15 lists genes that are repressed in response to YPD in committed cells. Table S16 includes a list of the yeast strains used in the present study. Table S17 includes the composition of the media used in the present study. [file gb-2006-7-3-r20-S3.pdf]

Supplementary Table S1: Early sporulation Genes

The early sporulation genes are listed. The average pattern (log_2ratio) is plotted upon transfer to YPD (which was followed for 5, 20 and 40 minutes after the transfer). The genes in each group, along with a one line description, are indicated.
Template genes used to create this group are: *ZIP1*, *HOP1*, *HOP2* and *SPO16*.

Average expression: 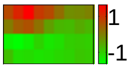

SNC1; YAL030W Synaptobrevin (v-SNARE) homolog present on post-Golgi vesicles
ACS1; FUN44; YAL054C Acetyl-CoA synthetase
RFA1; BUF2; (RPA1); FUN3; SRR1; YAR007C DNA replication factor A, 69K subunit, binds single-stranded DNA
NTH2; YBR0106; YBR001C Putative secondary neutral trehalase (alpha, alpha-trehalase), may catalyze conversion of trehalose to glucose
MUM2; YBR0514; YBR057C; SPOT8 Protein required for premeiotic DNA synthesis and sporulation
YBR090C; YBR0811b Protein of unknown function
YBR113W; YBR0908e Protein of unknown function
NPL4; YBR1231; YBR170C Nuclear pore protein
UMP1; YBR1234; YBR173C Proteasome maturation factor chaperone involved in proteasome assembly
YBR184W; YBR1306 Protein of unknown function
PCH2; YBR1308; YBR186W Protein required for cell cycle arrest at the pachytene stage of meiosis in a zip1 mutant, has similarity to Rpt5p andNSF vesicular fusion protein and other members of the AAA family of ATPases
POP4; YBR1725; YBR257W Protein component of both the RNase MRP and RNase P ribonucleoproteins, which are involved in rRNA and tRNAprocessing respectively
YBR280C; YBR2017 Protein with similarity to Srm1p/Prp20p
PRD1; YCL434; YCL057W Proteinase yscD (saccharolysin), contains the zinc metalloendoprotease motif HEXXH
YCR062W; YCR061W; YCR904 Protein of unknown function has predicted transmembrane segments and similarity to Ytp1p
YCR082W Protein with similarity to Rbk1p
YCR105W NADPH-dependent cinnamyl-alcohol dehydrogenase, has similarity to bovine alcohol dehydrogenase
CDC7; SAS1; (OAF2); D2855; YDL017W Protein kinase that acts in complex with Dbf4p to affect initiation of DNA synthesis, commitment tosporulation, DNA repair, and meiotic recombination
YDL046W; D2699 Protein of unknown function
RPN5; (RPN4); D1572; YDL147W Non-ATPase subunit of the 26S proteasome complex
MSH5; D1542; YDL154W Meiosis-specific protein involved in reciprocal recombination not involved in mismatch repair
YDL187C; D1276 Protein of unknown function questionable ORF
YDL199C; D1209 Member of the hexose transporter family of the major facilitator superfamily (MFS)
COS7; D0215; YDL248W Member of the COS family of subtelomerically-encoded proteins
YDR015C; PZF129; D3234 Protein of unknown function
YDR018C; D3246; YD9335.04 Putative acyltransferase with similarity to Ybr042p
DBF4; DNA52; MTS2; D4205; YD9609.07; YDR052C Regulatory subunit for Cdc7p protein kinase, required for G1/S transition
SDH4; YD9395.11; YDR178W Membrane anchor subunit in the succinate dehydrogenase complex
SPC19; YD9346.11; YDR201W Protein of the spindle pole body
CHL4; CTF17; MCM17; YD9320A.04; YDR254W Chromosome segregation protein required for stable maintenance and transmission of normaland artificial chromosomes
YDR275W; D9954.3 Protein of unknown function
ZIP1; D9819.9; YDR285W Structural protein of the synaptonemal complex central element

has predicted coiled-coil domain
HNT2; APH1; D9740.15; YDR305C Diadenosine polyphosphate hydrolase member of histidine triad (HIT) protein superfamily
NUF1; SPC110; D9476.3; YDR356W Spindle pole body component with coiled-coil structure, determines the spacing between the ends ofmicrotubules and the central plaque
YDR374C; D9481.16 Protein of unknown function
ECM11; D9461.31; YDR446W Protein possibly involved in cell wall structure or biosynthesis
YDR506C; D9719.12 Protein with similarity to Fet5p
CIN8; KSL2; SLN2; SDS15; CST16; YEL061C Kinesin-related protein involved in establishment and maintenance of mitotic spindle
YER010C Protein of unknown function
PRO3; (ORE2); YER023W Delta-1-pyrroline-5-carboxylate reductase (P5C reductase), catalyzes the third and last step in proline biosynthesispathway
YER084W Protein of unknown function
BOI2; BEB1; YER114C Protein involved in bud formation that binds to Bem1p, has an SH3 domain
DMC1; (MED1); ISC2; YER179W Meiosis-specific recombination protein structurally related to S. cerevisiae Rad51p, Rad55p, Rad57p, and to E.coli RecA
MSH4; YFL003C Meiosis-specific protein homologous to E. coli MutS
YFL006W Protein of unknown function
TUB2; SHE8; ARM10; YFL037W Tubulin beta chain, required for mitosis and karyogamy
YFL044C Protein of unknown function, has a single C2H2-type zinc finger
RPN12; NIN1; (RPN11); YFR052W Non-ATPase component of 26S proteasome complex required for activation of Cdc28p protein kinase
HOP2; G3642; YGL033W Protein required for pairing of homologous chromosomes in meiosis
MTC2; G3632; YGL036W Protein of unknown function
MPS2; MMC1; G3219; YGL075C Protein of the nuclear envelope/endoplasmic reticulum required for spindle pole body assembly and normalchromosome segregation
RAD54; G1821; YGL163C DNA-dependent ATPase of the Snf2p family, required for mitotic recombination and DNA repair of X-ray damage
YGL183C; G1604 Protein of unknown function
IME4; SPO8; G1337; YGL192W Positive transcription factor for IME1 and IME2, mediates control of meiosis by carrying signals regarding matingtype (a/alpha) and nutritional status
SHE10; G0954; YGL228W Protein that causes lethality when overexpressed
DOC1; APC10; HRC283; G0910; YGL240W Component of the anaphase-promoting complex (APC), required for Clb2p degradation and for themetaphase-anaphase transition
ZIP2; MRD1; NRC704; G0568; YGL249W Protein involved in meiotic recombination, chromosome synapsis, and synaptonemal complexformation
HFM1; MER3; GOP NRE1041; G0562; YGL251C DNA/RNA helicase involved in meiotic recombination, member of the DExH-box type helicasefamily with Ski2p and Brr2p
PRE9; (PRS5); Y13; G6405; YGR135W Proteasome subunit alpha3_sc
YGR168C; G7059 Protein with similarity to human vasoactive intestinal peptide receptor PIR:JN0604
RIM4; NOS1; YHL024W Protein required for sporulation and formation of meiotic spindle, has two RNA recognition motif (RRM) domains
COS8; YHL048W Member of the COS family of subtelomerically-encoded proteins
YNG2; NBN1; YHR090C Component of NuA4 histone acetyltransferase complex with effect on BEM and RAD phenotypes
ERP5; YHR110W Protein with similarity to COPII-coated vesicle proteins Emp24p and Erv25p
SPO12; YHR152W Sporulation protein required for chromosome division in meiosis I
SPO16; YHR153C Early meiotic protein required for efficient spore formation
REC104; YHR157W Meiosis-specific protein, mutants are defective in synaptonemal complex formation and have reduced recombination
YHR202W Protein of unknown function
YIL024C; YI3299.07 Protein of unknown function
IRR1; (SCC3); YI3299.05; YIL026C Component of cohesin complex required for sister chromatid cohesion during DNA replication
ULP2; SMT4; YI9905.17; YIL031W Smt3p-specific protease, degrades conjugated ubiquitin-

like protein Smt3p
HOP1; YIL072W Meiosis-specific protein associated with lateral elements of the synaptonemal complex, involved in homologous chromosomesynapsis and chiasmata formation
SPO22; YIL073C Protein required for meiosis and sporulation, has cecropin family signature
YIL152W Protein of unknown function
HXT12; YI9402.06B; YI9402.06AW; YIL170W; YIL171W Member of the hexose transporter family of the major facilitator superfamily (MFS)
PRE3; CRL21; J1407; YJL001W Proteasome subunit beta1_sc involved in peptidyl-glutamyl peptide hydrolytic activity of the proteasome
SNX4; J1244; YJL036W Putative nexin sorting protein possibly involved in proteasome function
YJL045W; J1194 Protein with strong similarity to succinate dehydrogenase flavoprotein, has an FAD-binding domain
SIP4; J0922; YJL089W Transcriptional activator of gluconeogenic genes through CSRE elements, activated by Snf1p kinase, contains aZn[2]-Cys[6] fungal-type binuclear cluster domain
GLG2; J0663; YJL137C Self-glucosylating initiator of glycogen synthesis
YJL149W; J0634 Protein of unknown function, contains a cyclin F-box
YJL178C; J0490 Protein of unknown function
HXT9; HRC567; J0222; YJL219W Member of the hexose transporter family of the major facilitator superfamily (MFS)
IME1; J1916; YJR094C Transcription factor required for sporulation, positive regulator of IME2 and many sporulation genes
YJR098C; J1936 Protein of unknown function
COS5; J2410; YJR161C Member of the COS family of subtelomerically-encoded proteins
PUT3; YKL015W Transcription factor that activates the proline utilization pathway genes, contains a Zn[2]-Cys[6] fungal-type binuclear clusterdomain in the N-terminal region
MDH1; ACN50; YKL085W Malate dehydrogenase, mitochondrial
YKL121W; YKL525 Protein of unknown function, member of WD (WD-40) repeat family
SDH3; CYB3; YKL4; YKL141W Membrane anchor subunit for Sdh1p in the succinate dehydrogenase complex
YKR005C; YK104 Protein of unknown function
TOF2; YK109; YKR010C Protein that interacts with DNA topoisomerase I
YKR083C; YKR403 Protein with similarity to paramyosin
YLL030C; L0932 Protein with similarity to Ca2+transporting ATPase
GPI13; MPC1; L0929; YLL031C Protein involved in glycosylphosphatidylinositol biosynthesis, probably responsible for addingphosphoethanolamine to the third mannose
RNP1; L0713; YLL046C Ribonucleoprotein 1, contains two RNA recognition (RRM) domains which include RNP-1 octamer and RNP-2 hexamermotifs
YLL047W; L0709 Protein of unknown function
PCD1; L9634.8; YLR151C; L3325 Peroxisomal nudix hydrolase, has diphosphatase activity specific for coenzyme A and CoA derivatives
YLR346C; L8300.14 Protein of unknown function
CTR3; L9931.6; YLR411W High-affinity copper transporter
YLR446W; L9324.5 Protein with similarity to human hexokinase I
HMG2; L9324.2; YLR450W 3-Hydroxy-3-methylglutaryl-coenzyme A reductase 2, rate limiting enzyme for sterol biosynthesis, converts HMG-CoAto mevalonate
RAD52; YML032C Protein required for recombination and repair of X-ray damage, has a late function in meiotic recombination
CAT2; YCAT CAT YM8054.01; YML042W Carnitine O-acetyltransferase, peroxisomal and mitochondrial
MFT1; MFT52; YML062C Targeting factor for mitochondrial precursor proteins, member of a family of transmembrane transition metal transporters
TUB1; YML085C Tubulin alpha-1 chain, required for mitosis and karyogamy
YML122C; YM7056.04 Protein of unknown function, questionable ORF
COS3; YM4987.03; YML132W Member of the COS family of subtelomerically-encoded proteins
AAC1; (ANC1); YM9796.09; YMR056C ADP/ATP carrier protein of the mitochondrial carrier family (MCF) of membrane transporters

PDS5; SPO27; YM9582.01; YM9916.15; YMR076C Protein required for sister chromatid cohesion and chromosome condensation functions
MUB1; YM6543.07; YMR100W Zinc finger protein, involved in the regulation of bud site selection
YMR101C; SRT1; YM6543.08 Protein that acts as suppressor of rer2 mutation, has similarity to Rer2p
YMR107W; YM9718.06 Protein of unknown function, induced during aerobic growth
SAS2; ESO1; YM9553.03; YMR127C Protein involved in silencing at HMR, has a single C2H2-type zinc finger
REC114; YM9375.02; YMR133W Meiotic recombination protein, mutants are defective in double-strand break formation
YMR134W; YM9375.03 Protein involved in iron metabolism
YMR148W; YM9375.18 Protein of unknown function
SGS1; (TPS1); YM9646.02; YMR190C DNA helicase of DEAD/DEAH family, has similarity to the human Werner's and Bloom's syndromes genesand to E. coli RecQ 3'-5' DNA helicase
YMR299C
FKS3; YM9952.08; YMR306W Protein with similarity to Fks1p and Gsc2p
SPO1; N2858; YNL012W Transcriptional regulator involved in sporulation has strong similarity to phospholipase B enzymes
YNL116W; N1925 Protein of unknown function
YNL150W; N1773 Protein of unknown function
SLZ1; N1386; YNL196C Sporulation-specific protein
COS1; N0275; YNL336W Member of the COS family of subtelomerically-encoded proteins
HTZ1; HTA3; O2345; YOL012C Histone-related protein that can suppress histone H4 point mutation
PKH2; HRC1081; O0784; YOL100W Serine/threonine protein kinase with similarity to mammalian 3-phosphoinositide-dependent protein kinase
YOL131W; AOA108; O0517 Protein of unknown function
ASE1; O2806; YOR29-09; YOR058C Microtubule-associated protein localized to the spindle midzone, required for anaphase spindle elongation
YOR073W; YOR29-24; O2945 Protein of unknown function
RTS2; O2956; YOR29-28; YOR077W Protein with similarity to mouse KIN17 protein, has a single C2H2-type zinc finger
LSC1; YOR3352; O3352; YOR142W Alpha subunit of succinyl-CoA synthetase (succinyl-CoA ligase, succinate thiokinase)
SMP3; LAS2; SAP2; O3527; YOR149C Protein required for plasmid maintenance functions in the protein kinase C pathway
GAC1; O4625; YOR178C Regulatory subunit for protein serine/threonine phosphatase Glc7p
RPT4; SUG2; CRL13; PCS1; O5345; YOR259C Component of 26S proteasome complex and member of the AAA family of ATPases
RPN8; (RPN7); NAS3; O5360; YOR261C Non-ATPase subunit of the 26S proteasome complex
SNF2; SWI2; GAM1; TYE3; HAF1; (RIC1); MNU1; O5509C; YOR290C Component of SWI-SNF global transcription activator complex, acts toassist gene-specific activators through chromatin remodeling
FAA1; O6136; YOR317W Long-chain fatty acid CoA ligase (fatty acid activator 1) can incorporate exogenous myristate into myristoyl-CoA andother fatty acids to the CoA derivatives
CIN1; O6350; YOR349W Protein involved in chromosome segregation, required for microtubule stability
MNE1; O6353; YOR350C Protein of unknown function
MEK1; MRE4; O6357; YOR351C Serine/threonine protein kinase required for meiotic recombination
RAD17; O6670; YOR368W Checkpoint protein required for DNA damage-induced arrest in G2 at the RAD9 checkpoint
NDD1; O6682; YOR372C Protein required for nuclear division positively but indirectly affects transcription of a subset of genes required for thecell cycle
YPL034W; P7102.15 Protein of unknown function
CAR1; (AGA2); LPH15; ARG cargA casn1; otA YPL111W Arginase, catalyzes the first step in arginine degradation and acts as an allostericregulator of ornithine carbamyltransferase

MEI5; LPH6; YPL121C Meiotic protein required for synapsis and meiotic recombination
MLH3; YPL164C; P2550 Insertion and deletion mismatch repair protein
CIT3; LPZ1W; YP9723.01; YPR001W Citrate synthase, mitochondrial, converts acetyl-CoA and oxaloacetate into citrate plus CoA
PDH1; YP9723.02; LPZ2W; YPR002W Protein possibly involved in propionate utilization
SPO69; YP9723.07; LPZ7C; REC8; YPR007C Protein required for meiosis and sporulation
ATP20; SU_G YP9531.14; YPR020W F1F0-ATP synthase complex F0 membrane domain g subunit
YPR061C; YP9499.16 Protein with similarity to E. coli DnaJ and other DnaJ-like proteins
KIM3; P9325.4; YPR164W Protein involved in resistance to mutagens such as diepoxybutane and mitomycin C
YMR052C-A Protein of unknown function
MEI4; YER044C-A Protein required early in meiosis for meiotic recombination, chromosome synapsis, and viable spore formation
YMR306C-A Protein of unknown function
YMR119W-A Protein of unknown function

Supplementary Table S2

complete list of genes used in figure 3d

cluster 1 (early sporulation)

RAD51	RAD51; MUT5; YER095W Protein that stimulates pairing and strand-exchange between homologous single-stranded and double-stranded DNA,functionally similar to E. coli RecA protein
RFA2	RFA2; BUF1; N0368; YNL312W DNA replication factor A, 36K subunit phosphorylated at the G1/S transition and dephosphorylated at mitosis
PIF1	PIF1; TST1; YM9958.01; YML061C Single-stranded DNA-dependent ATPase and 5'-3' DNA helicase required for maintenance and repair ofmitochondrial DNA, also functions in nucleus to regulate telomere length
CAC2	CAC2; YML102W Chromatin assembly complex subunit 1, involved in nucleosome assembly linked with DNA replication, has WD (WD-40)repeats
RFA1	RFA1; BUF2; (RPA1); FUN3; SRR1; YAR007C DNA replication factor A, 69K subunit, binds single-stranded DNA
UBC13	UBC13; YD6652.04; YDR092W Ubiquitin-conjugating (E2) enzyme involved in Rad6-dependent post-replicative repair pathway
RFA3	RFA3; J0506; YJL173C DNA replication factor A, 13K subunit
RAD54	RAD54; G1821; YGL163C DNA-dependent ATPase of the Snf2p family, required for mitotic recombination and DNA repair of X-ray damage
REC104	REC104; YHR157W Meiosis-specific protein, mutants are defective in synaptonemal complex formation and have reduced recombination
RAD17	RAD17; O6670; YOR368W Checkpoint protein required for DNA damage-induced arrest in G2 at the RAD9 checkpoint
RAD53	RAD53; MEC2; SPK1; (SAD1); P2588; YPL153C Serine/threonine/tyrosine protein kinase with a checkpoint function in S and G2
KIM3	KIM3; P9325.4; YPR164W Protein involved in resistance to mutagens such as diepoxybutane and mitomycin C
YDR386W	MUS81; SLX3; D9509.6; YDR386W Protein involved in repair of DNA damage, part of a complex with Rad54p and Mms4p
YER104W	RTT105; YER104W Protein that inhibits transposable element Ty1 transposition
REC107	REC107; MER2; J1462; YJR021C Meiotic recombination protein, component of a complex that processes double-stranded DNA breaks
KIM2	REM50; KIM2; L1377; YLL002W Protein involved in resistance to mutagens such as diepoxybutane and mitomycin C
MSH4	MSH4; YFL003C Meiosis-specific protein homologous to E. coli MutS
YGL183C	YGL183C; G1604 Protein of unknown function
YIR025W	YIR025W Protein of unknown function
RAD52	RAD52; YML032C Protein required for recombination and repair of X-ray damage, has a late function in meiotic recombination
REC114	REC114; YM9375.02; YMR133W Meiotic recombination protein, mutants are defective in double-strand break formation

YPL164C	MLH3; YPL164C; P2550 Insertion and deletion mismatch repair protein
DMC1	DMC1; (MED1); ISC2; YER179W Meiosis-specific recombination protein structurally related to S. cerevisiae Rad51p, Rad55p, Rad57p, and to E.coli RecA
YHL024W	RIM4; NOS1; YHL024W Protein required for sporulation and formation of meiotic spindle, has two RNA recognition motif (RRM) domains
HHO1	HHO1; LPI17; YPL127C Histone H1

cluster 2 (middle sporulation)

RAD57	RAD57; YD8119.10; D2970; YDR004W Component of recombinosome complex involved in meiotic recombination and recombinational repair,with Rad55p promotes DNA strand exchange by Rad51p recombinase
TFB1	TFB1; D9740.3; YDR311W Component of RNA polymerase II transcription initiation factor TFIIH (factor b), 75 kDa subunit
IMP2'	YIL154C; (IMP2); BLM25; HCY39 Protein involved in control of mitochondrial sugar utilization and repair of oxidative DNA lesions
MSH5	MSH5; D1542; YDL154W Meiosis-specific protein involved in reciprocal recombination not involved in mismatch repair
MFT1	MFT1; MFT52; YML062C Targeting factor for mitochondrial precursor proteins, member of a family of transmembrane transition metal transporters
SAE2	SAE2; COM1; G1639; YGL175C Protein involved in meiotic recombination
SPO11	SPO11; YHL022C Catalytic subunit of the meiotic double strand break transesterase
TFB2	TFB2; LPH5; YPL122C Component of RNA polymerase II transcription initiation TFIIH (factor b), 55 kDa subunit
DIN7	DIN7; DIN3; YD9320B.02; YDR263C Mitochondrial inner membrane nuclease with a role in stabilizing the mitochondrial genome expression isinduced by DNA damage
REV7	REV7; YIL139C DNA polymerase zeta, small subunit, required for mutagenesis by physical and chemical agents
DHS1	EXO1; DHS1; O2727; OR26.23; YOR033C Double-stranded DNA 5'->3' exonuclease, involved in mismatch repair and recombination
REV3	REV3; PSO1; P2535; YPL167C DNA polymerase zeta, involved in mutagenic translesion DNA repair synthesis
HRR25	HRR25; (RST2); P1850; YPL204W Casein kinase I (serine/threonine/tyrosine protein kinase) involved in DNA repair and meiosis
RAD7	RAD7; GTA565; UVS7; J1665; YJR052W Nucleotide excision repair protein involved in G2 repair of inactive genes

cluster 3 (Return to growth)

RAD16	RAD16; PSO5; YBR0909; YBR114W Nucleotide excision repair protein involved in G2 repair of inactive genes, has DNA helicase domain ofSnf2p family

YMR137C	PSO2; (SNM1); YM9375.06; YMR137C DNA repair protein for interstrand crosslinks
YDR545W	YRF1-1; (YRF1); D3703.4; YDR545W Protein with near identity to the family of subtelomerically-encoded proteins including Yil177p, Yhl049p, andYjl225p
NUC1	NUC1; HRE329; J0310; YJL208C Nuclease with both DNase and RNase activity, major nuclease of mitochondria
CDC2	CDC2; POL3; TEX1; HPR6; MUT7; D2366; YDL102W DNA polymerase delta large subunit
FOB1	FOB1; HRM1; YD9727.06; YDR110W Protein required for blocking the replication fork, for recombinational hotspot activity at the HOT1 site inrDNA, and for expansion and contraction of rDNA repeats
YGL150C	INO80; G1880; YGL150C Member of the Snf2p-like family of probable DNA helicases
HAM1	HAM1; J1811; YJR069C Protein controlling 6-N-hydroxylaminopurine sensitivity and mutagenesis
RAD10	RAD10; UVS14; YML095C Component of the nucleotide excision repairosome, homolog of mammalian ERCC-1 gene product
YBR223C	YBR223C; TDP1; YBR1520 Tyrosyl-DNA phosphodiesterase, involved in DNA repair
PAN2	PAN2; G3165; YGL094C Component of Pab1p-stimulated poly(A) ribonuclease
THI4	THI4; MOL1; ESP35; G6620; YGR144W Thiamine-repressed protein essential for growth in the absence of thiamine
RFC2	RFC2; CST15; J1808; YJR068W Replication factor C, second subunit, homologous to human 37 kDa subunit
SIR3	SIR3; MAR2; CMT1; STE8; L9753.10; YLR442C Protein involved in maintenance of silencing of HMR, HML, and telomeres, member of theAAA+ family of putative ATPases
RFC5	RFC5; YBR0810; YBR087W Replication factor C, 40 kDa subunit
RAD55	RAD55; XS9; D4426; YD8554.09; YDR076W Component of recombinosome complex involved in meiotic recombination and recombinationalrepair with Rad57p promotes DNA strand exchange by Rad51p recombinase
CCE1	CCE1; (MGT1); YKL164; YKL011C Cruciform cutting endonuclease
TOP3	TOP3; EDR1; L8083.3; YLR234W DNA topoisomerase III, relaxes negatively (but not positively) supercoiled DNA
RFC3	RFC3; N0533; YNL0533; YNL290W Replication factor C, 40 kDa subunit, member of the DEAD box family of putative helicase proteins
YHR081W	YHR081W Protein of unknown function
MSH1	MSH1; YHR120W Protein involved in mitochondrial DNA repair homolog of E. coli MutS
YNL133C	FYV6; N1215; N1850; YNL133C Protein required for viability following exposure to toxin
YER160C	RAD3; REM1; UVS4; YER171W DNA helicase component of RNA polymerase II transcription initiation factor TFIIH (factor b), also a componentof the nucleotide excision repairosome
RAD3	
RFC4	RFC4; O0923; YOL094C Replication factor C, 37 kDa subunit
CTF4	CTF4; POB1; CHL15; P9659.7; YPR135W Protein required for DNA synthesis, binds DNA polymerase alpha
YER116C	SLX8; YER116C Subunit of Hex3p-Slx8p complex, required for cell growth in the absence of SGS1 or TOP3
RAD27	RAD27; RTH1; ERC11; (FEN1); YKL510; YKL113C Single-stranded DNA endonuclease and 5'-3' exonuclease that functions in theMSH2-MLH1-PMS1-dependent mismatch repair system

MSI1	MSI1; CAC3; YBR1406; YBR195C Chromatin assembly complex, subunit 3, involved in both nucleosome assembly linked with DNA replicationand negative regulation of the RAS pathway, has WD (WD-40) repeats
YHR167W	THP2; YHR167W Protein with possible roles in transcription and recombination

cluster 4 (transient)

SOH1	SOH1; G2864; YGL127C Protein that allows hpr1 null mutant to grow at 37 deg
RPH1	RPH1; YMR137C DNA damage-responsive repressor of PHR1, contains two C2H2-type zinc finger domains
NSE1	NSE1, YLR007W;Protein that may function with Smc5p and Rhc18p in a complex required for DNA repair and proliferation

Supplementary Table S3: Expression of mitotic cell cycle regulated genes during RTG

Some examples of cell cycle regulated genes (identified by Spellman et al., 1998) during M/G1, G1, S/G2 and G2/M.
In each of these groups, some of the genes are induced and some are repressed/non induced durng RTG.
Also, some examples are given for genes that are induced during the S-phase of the mitotic cell cycle (Spellman et al., 1998)
and are induced during RTG only when the transfer was done early enough (2 hours in SPM) but not later.

M/G1		G1		S/G2		G2/M		S
M/G1 genes repressed during RTG	M/G1 genes induced during RTG	G1 genes induced during RTG	G1 genes not induced during RTG	S/G2 genes Induced during RTG	S/G2 genes not induced during RTG	G2/M genes not induced during RTG	G2/M genes induced during RTG	Induced during RTG only at early transfer time
YGP1	*YOR066W*	*YHL049C*	*YBR071W*	*ARG1*	*YOR391C*	*IDH1*	*PDR5*	*HHF1*
YFL044C	*HXT4*	*RFC4*	*QRI1*	*ASN2*	*CLB4*	*YBR242W*	*YIL011W*	*HHF2*
YNL134C	*MCM3*	*CDC2*	*SUR1*	*BAT1*	*YLR373C*	*NUF2*	*YOL014W*	*HHT1*
YOR264W	*YHR022C*	*RAD27*	*GPH1*	*YGL101W*	*YER018C*	*NDI1*	*YPR013C*	*HTB2*
YBR296C	*YDR157W*	*YAR003W*	*YPL267W*	*YCK1*	*YKL035W*	*YNL171C*	*PFK1*	*HHT2*
YOR052C	*HXT7*	*POL12*	*YEL064C*	*YML125C*	*YIL135C*	*APC1*	*YOR258W*	*HTB1*
HXT10	*RGA1*	*JEM1*	*PBI2*	*CYC1*	*CWP1*	*ARO9*	*YKL172W*	*HTA1*
GFA1	*YNL078W*	*CTF4*	*PDS5*	*ERG3*	*CHS6*	*YFR039C*	*YPR156C*	*HTA2*
YOR242C	*UTR2*	*YNL231C*	*RFA1*	*YDR346C*	*YER042W*	*WSC4*	*STP4*	*PMI40*
DBI56	*YJL159W*	*YGR151C*	*LAP4*	*SNQ2*	*YLR209C*	*DIP5*	*CLN3*	*EXG2*
SIC1	*YOR342C*	*PMT3*	*YHR110W*	*YGR138C*	*YHR146W*	*CAR1*	*YDR033W*	*FLR1*
RNH70	*YPL014W*	*MKC7*	*YKL066W*	*AUR1*	*YDR130C*	*CDC5*	*YLR297W*	*HTA3*
IAH1	*ROD1*	*RFC5*	*YOR084W*		*VAM7*	*HST3*	*YKR079C*	*YET1*
YGR086C	*YHB1*	*YLR236C*	*YNL208W*		*BUB2*	*CLB1*	*YLR413W*	*ERV25*
YGL037C	*SKT5*	*YBR089W*	*OPY2*		*SCC3*	*CAR2*	*YRO2*	*SLT2*
GSY1		*PMT1*	*YLR049C*		*PCL7*	*MOB1*		*YHR098C*
FAA1		*TRR1*	*DHS1*		*GLG2*	*YDR276C*		*YJL118W*
MDG1		*HSL1*	*CLB6*		*PET8*	*IQG1*		
KAR4		*YBR007C*	*CLB5*		*HOP1*	*YLR084C*		
YIR036C		*YGL060W*	*EPT1*		*PUT1*	*YOL132W*		

WTM1		*EXG1*	*CNM1*		*ORC1*	*NCE102*		
TIP1		*FKS1*	*YGR153W*		*TUB2*	*YLR034C*		
YBL064C			*YLR343W*		*YDR149C*	*ASE1*		
YBR273C			*SPC98*		*YLR437C*	*YLR190W*		
YBR204C			*PDS1*		*TPM2*	*YBR139W*		
GLK1			*KAR3*		*YLR099C*	*YML066C*		
					YOR152C	*CDC20*		
					YPL141C	*YOR383C*		
					YML058W	*SPS4*		
					YKL183W	*SUT1*		
					CDC10	*YOR273C*		
					GAP1	*YBR094W*		
					DBF20	*SPO12*		
					NUM1	*YMR253C*		
						SKN1		
						YHR151C		
						YGR284C		
						YMR031C		
						YHL026C		
						KIN3		
						YDR380W		
						YFL006W		

Spellman, P. T., Sherlock, G., Zhang, M. Q., Iyer, V. R., Anders, K., Eisen, M. B., Brown, P. O., Botstein, D., and Futcher, B. (1998).
Comprehensive identification of cell cycle-regulated genes of the yeast Saccharomyces cerevisiae by microarray hybridization. Mol Biol Cell 9, 3273-3297.

Supplementary Table S4: genes that are induced during RTG

The average pattern ($\log_2$ratio) is plotted upon transfer to YPD (which was followed for 5, 20 and 40 minutes after the transfer). The genes in the group, along with a one line description, are indicated.
Template genes used to create this group are: *RFC2, YJL200C, YLR413W, YGR105W, YPR044C, YJL188C, YBR239C, URA5, VMA21, CTF4, YGL016W, YGL042C, SIR3, YLR344W*

Group 6	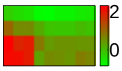
ERP2; (FUN54); YAL007C Component of COPII-coated vesicles, forms an Erp1p-Erp2p-Emp24p-Erv25p heteromeric complex, has similarity toEmp24p and Erv25p	
ECM1; (SIM1); YAL059W Protein possibly involved in cell wall structure or biosynthesis	
YBL070C; YBL0616 Protein of unknown function	
BOI1; (BOB1); YBL0717; YBL085W Bem1p-binding protein, has an SH3 domain and a PH (pleckstrin) domain	
YBR089W; YBR0811a Protein of unknown function, questionable ORF	
YBR197C; YBR1409 Protein of unknown function	
YBR239C; YBR1622 Protein with similarity to transcription factors, has Zn[2]-Cys[6] fungal-type binuclear cluster domain in the N-terminal region	
DUT1; YBR1705; YBR252W dUTP pyrophosphatase, converts dUTP to dUMP thereby preventing uracil incorporation into DNA	
RER1; YCL001W Component of COPII-coated vesicles, involved in retrieval of endoplasmic reticulum membrane proteins from the early Golgicompartment	
HIS4; YCL183; YCL030C Phosphoribosyl-AMP cyclohydrolase / phosphoribosyl-ATP pyrophosphohydrolase / histidinol dehydrogenase,second, third, and tenth steps of histidine biosynthesis pathway	
YCR087W Protein of unknown function	
YDL060W; D2544 Protein of unknown function	
YHM1; (SHM1); D1214; YDL198C Protein of the mitochondrial carrier (MCF) family that when overexpressed suppresses loss of Abf2p	
NHP2; D1045; YDL208W Nucleolar protein required in association with H/ACA snoRNAs for ribosomal RNA pseudouridinylation	
RLI1; YD6652.03; YDR091C Putative ortholog of human Rnase L inhibitor (RLI) of the interferon-regulated 2-5A pathway, putative ortholog of C.elegans Y39E4B.1, member of the non-transporter group in the ATP-binding cassette (ABC) superfamily	
MKC7; YPS2; YD8358.01; YD2943.03; YDR144C Aspartyl protease found in the periplasmic space, has similarity to Yps1p and Bar1p	
ASP1; D9798.6; YDR321W Asparaginase, (L-asparagine aminohydrolase I), intracellular isozyme that converts asparagine to aspartate andammonia	
TIF35; D9461.16; YDR429C Translation initiation factor eIF3, p33 subunit, contains an RRM (RNA recognition motif) domain	
YEL018W Protein of unknown function	
UTR2; CRH2; SYGP-ORF18; YEL040W Cell wall protein	
PTC2; YER089C Protein serine/threonine phosphatase of the PP2C family, negative regulator of unfolded protein response (UPR) pathway	
AST2; YER101C Protein with similarity to Ast1p	
KAP122; PDR6; G3713; YGL016W Nuclear transport factor, member of karyopherin-beta family	
YGL042C; G3555 Protein with similarity to rat Na+/Ca2+ exchanger NCX2 PIR:A54139	
YGL144C; ROG1; G2525! Protein of unknown function, has strong similarity to Ydl109p and Ydr444p	
VMA21; G5943; YGR105W Protein required for vacuolar H(+)-ATPase (V-ATPase) assembly	

THI4; MOL1; ESP35; G6620; YGR144W Thiamine-repressed protein essential for growth in the absence of thiamine
YGR169C; G7062 Protein of unknown function
HGH1; G7538; YGR187C Protein with similarity to human HMG1 and HMG2 proteins
PHO81; (SPL1); ORF1178; G8567; YGR233C Cyclin-dependent kinase (CDK) inhibitor for Pho80p-Pho85p cyclin-dependent protein kinasecomplex, positive regulator of phosphate pathway
PDR13; H8025.17; YHR064C Pleiotropic drug resistance protein, member of the Hsp70 family of heat shock proteins
RPL40A; UBI1; CEP52A; CST2; YIL148W Fusion protein whose N-terminal half is ubiquitin and whose C-terminal half is ribosomal protein L40(rat L40), identical to Rpl40Bp
YIR035C Protein with similarity to proteins of the human corticosteroid 11-beta-dehydrogenase and short-chain alcohol dehydrogenase family
BUD19; J0403; YJL188C Protein of unknown function questionable ORF
YJL200C; J0327 Protein with similarity to aconitase, has potential mitochondrial transit peptide
ILV3; (ISO1); VAL J1450; YJR016C Dihydroxyacid dehydratase (DAD), third step in valine and isoleucine biosynthesis pathway
YJR041C; GTF1174; J1622 Protein with similarity to Podospora anserina NADH dehydrogenase chain 4
RFC2; CST15; J1808; YJR068W Replication factor C, second subunit, homologous to human 37 kDa subunit
NMD5; J2112; KAP119; YJR132W Member of the karyopherin-beta family involved in nuclear transport
YKL051W; YKL303 Protein of unknown function
DPH2; YKL191W Diptheria toxin resistance protein, required for diphthamide biosynthesis
RPL40B; UBI2; CEP52B; CST1; YKR414; YKR094C Fusion protein comprised of ribosomal protein L40 (C-terminal half) and ubiquitin(N-terminal half), (rat L40), identical to Rpl40Ap
YLL044W; L0725 Protein of unknown function
YLL059C; L0563 Protein of unknown function
YLR042C; L1929 Putative GPI-anchored protein of unknown function
BUD28; L2171; YLR062C Protein that may be involved in bud site selection in diploid cells
YLR076C; L2345 Protein of unknown function
SLS1; L3159; YLR139C Protein involved in mitochondrial metabolism
YLR179C; L9470.20 Protein with similarity to Tfs1p Cdc25p-dependent nutrient- and ammonia-response protein
SIK1; NOP56; L8167.9; YLR197W Nucleolar protein component of box C/D snoRNPs, which are necessary for 2'-O-methylation of ribosomalRNAs
YLR198C Protein of unknown function
RPS25B; RPS31B; L8300.10; YLR333C Ribosomal protein S25B (yeast S31; YS23; rp45; rat S25), identical to Rps25Ap
RPL26A; L8300.4; YLR344W Ribosomal protein L26 (yeast L33; YL33; rat L26), nearly identical to Rpl26Bp
RPS29A; L8084.11; YLR388W Ribosomal protein S29 (yeast S36; YS29; rat S29), similar to Rps29Bp
AFG2; DRG1; L8084.16; YLR397C Member of the AAA family of ATPases, has similarity to mammalian valosin-containing protein (VCP)
YLR401C; L8084.19 Protein with weak similarity to R. capsulatus protein nifR3
YLR413W; L9931.4 Protein of unknown function, induced during anaerobic growth
SIR3; MAR2; CMT1; STE8; L9753.10; YLR442C Protein involved in maintenance of silencing of HMR, HML, and telomeres, member of theAAA+ family of putative ATPases
FPR4; L9324.3; YLR449W Nucleolar peptidylprolyl cis-trans isomerase (PPIase)
IMD4; YM9958.06; YML056C Protein with similarity to inosine-5'-monophosphate dehydrogenase
RPL6A; YML073C Ribosomal protein L6 (yeast L17A; YL16A; human L6), nearly identical to Rpl6Bp
URA5; PYR5; YM8339.13; YML106W Orotate phosphoribosyltransferase 1, catalyzes the fifth step in the pyrimidine biosynthesis pathway

NIP1; YMR309C Subunit of translation initiation complex eIF3, also required for nuclear import has weak similarity to Nsr1p
YOL014W; O2333 Protein of unknown function
YOL037C; O2101 Protein of unknown function
SPE2; O1275; YOL052C S-adenosylmethionine decarboxylase, required for biosynthesis of pantothenic acid, post-translationally cleaved intoalpha and beta chains
ROD1; OR26.08; YOL303.7; O2627; YOR018W Protein that mediates resistance to o-dinitrobenzene (O-DNB)
CKA2; O2810; YOR29-12; YOR061W Casein kinase II (Protein kinase CK2), catalytic (alpha-prime) subunit
CAF20; CAP20; CAF2; O5453W; YOR276W mRNA cap-binding protein (eIF4F) 20K subunit
YOR376W; O6736 Protein with weak similarity to Na+/K+-exchanging ATPases
EGD1; CST25; P7102.13; YPL037C Beta subunit of the nascent polypeptide-associated complex
YPR044C Protein of unknown function
CTF4; POB1; CHL15; P9659.7; YPR135W Protein required for DNA synthesis, binds DNA polymerase alpha

Supplementary Table S5: genes that are induced immediately during RTG

The average pattern (log_2ratio) is plotted upon transfer to YPD (which was followed for 5, 20 and 40 minutes after the transfer). The genes in the group, along with a one line description, are indicated.
Template genes used to create this group are: *CLN3, YJL109C, STD1, YLR432W, YOR051C, DRS1*

Group 5	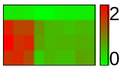
YAL019W; CST24; YAL001; FUN30 Protein of the Snf2p family with a DNA-dependent ATPase domain, involved in resistance to UV radiation	
POP5; FUN53; YAL033W Subunit of both RNase P and RNase MRP, which are involved in tRNA processing and 5.8S rRNA processing,respectively	
YAL036C; FUN11 Protein with similarity to Xenopus laevis DRG GTP-binding protein	
CLN3; DAF1; WHI1; CST7; FUN10; YAL040C G1/S-specific cyclin that interacts with Cdc28p protein kinase to control events at START	
URA7; YBL0410; YBL039C CTP synthase final step in pyrimidine biosynthesis pathway	
YBL054W; (YBL0509) Protein of unknown function	
RER2; YBR0107; YBR002C Cis-prenyltransferase, involved in dolichol synthesis	
PHO3; YBR0813; YBR092C Acid phosphatase, constitutive, thiamine-binding protein of the periplasmic space	
PHO88; YBR0835; YBR106W Membrane protein involved in inorganic phosphate transport	
YBR144C; YBR1121 Protein of unknown function	
RIB7; YBR1203; YBR153W HTP reductase, second step in the riboflavin biosynthesis pathway	
SMY2; YBR1233; YBR172C Protein of unknown function, suppresses myo2-66, sec22, bet1, sec16-3, spt15, and yrb1-51 mutants whenoverexpressed	
YPC1; YBR1305; YBR183W Alkaline ceramidase	
YBR238C; YBR1608 Protein of unknown function	
ENP1; MEG1; YBR1635; YBR247C Essential nuclear protein with effects on N-glycosylation of proteins in the secretory pathway	
DUT1; YBR1705; YBR252W dUTP pyrophosphatase, converts dUTP to dUMP thereby preventing uracil incorporation into DNA	
YBR271W; YBR1739 Putative SAM-dependent methyltransferase	
SRO9; SYS2; YCL037C Protein associated with ribosomes, has a potential role in translational accuracy, member of a subfamily of La motifproteins	
KRR1; YCL059C Protein essential for cell division and spore germination	
YCR016W Protein of unknown function	
YCR060W Protein with similarity to stress inducible protein Sti1p, has tetratricopeptide (TPR) repeats	
YCR072C Protein of unknown function, member of WD (WD-40) repeat family	
NOP1; D2870; YDL014W Fibrillarin, protein associated with small nucleolar RNA (snoRNA) and required for 35S rRNA processing andmethylation	
PRM7; D2723; YDL039C Protein of unknown function	
KRE26; D2717; YDL041W Protein of unknown function	
STP4; D2690; YDL048C Protein with strong similarity to Stp1p, which is involved in tRNA splicing and branched-chain amino acid uptake	
YDL060W; D2544 Protein of unknown function	
CDC2; POL3; TEX1; HPR6; MUT7; D2366; YDL102W DNA polymerase delta large subunit	
RRP42; D2315; YDL111C Component of exosome 3'-5' exoribonuclease complex that is required for 3' processing of ribosomal 5.8S rRNA, ofsnoRNAS, and of U4 snRNA	
YDL166C; FAP7; D1487 Essential protein important for Skn7p-dependent transcription of target genes during oxidative stress	
HEM3; D1057; YDL205C Porphobilinogen deaminase (pre-uroporphyrinogen synthase), catalyzes the third step in porphyrin biosynthesispathway	
NHP2; D1045; YDL208W Nucleolar protein required in association with H/ACA snoRNAs for	

ribosomal RNA pseudouridinylation
KRS1; GCD5; YD9673.09; D3457; YDR037W Lysyl-tRNA synthetase, cytoplasmic
RRP1; YD8554.20; D4478; YDR087C Protein involved in maturation of 25S rRNA
YDR093W; YD8557.01 Member of the Drs2p family of the P-type ATPase superfamily
FOB1; HRM1; YD9727.06; YDR110W Protein required for blocking the replication fork, for recombinational hotspot activity at the HOT1 site inrDNA, and for expansion and contraction of rDNA repeats
PSL10; YD9302.01; YD9727.21; YDR126W Protein of unknown function
SAN1; YD2943.02; YDR143C Protein that may antagonize the function of Spt16p and Sir4p
YDR152W; YD8358.08 Protein of unknown function
RPA14; YD8358.11; YDR156W RNA polymerase I subunit A14
YDR157W Protein of unknown function
YDR210W; YD8142A.07; YD8142.07 Protein of unknown function
YDR248C; YD8419.15 Protein with similarity to E. coli gluconate kinase gntV
YDR266C; D9954.10 Protein of unknown function, contains a RING finger domain
YDR365C; D9481.12 Protein of unknown function
YDR398W; D9509.17 Protein of unknown function
HPT1; BRA6; D9509.18; YDR399W Hypoxanthine-guanine phosphoribosyl transferase
YDR459C; D8035.3 Protein of unknown function, has similarity to Erf2p
YDR514C; D9719.19 Protein of unknown function
YEL018W Protein of unknown function
ANP1; GEM3; CAX1; MNN8; MCD2; SYGP-ORF28; YEL036C Protein of the cis Golgi with a role in retention of glycosyltransferases in the Golgi,subunit of the Anp1p-Hoc1p-Mnn11p-Mnn9p mannosyltransferase complex
YEL045C; SYGP-ORF33 Protein of unknown function, has motifs typical of ATP/GTP binding sites
YEL048C; SYGP-ORF36 Protein of unknown function
YER028C Protein with similarity to Mig2p, Tup1p-dependent and glucose-dependent transcriptional repressor with unknown function
KRE31; YER082C Protein of unknown function, has two tandem C2H2-type zinc fingers, has one WD (WD-40) domain
ILV1; (ISO1); YER086W Serine and threonine dehydratase (anabolic), first step in isoleucine biosynthesis pathway
PTC2; YER089C Protein serine/threonine phosphatase of the PP2C family, negative regulator of unfolded protein response (UPR) pathway
LCP5; SYGP-ORF43; YER127W Protein required for ribosomal RNA processing
YGL042C; G3555 Protein with similarity to rat Na+/Ca2+ exchanger NCX2 PIR:A54139
YGL108C; G3070 Protein of unknown function
RAI1; NRE387; G0580; YGL246C Protein that binds and enhances function of nuclear exonuclease Rat1p
PAC10; GIM2; G4579; YGR078C Prefoldin subunit 3, component of the Gim protein complex that promotes formation of functional alpha- andgamma-tubulin, and actin
YGR103W; G5933 Nuclear protein of unknown function with similarity to zebrafish pescadillo
FYV13; G7004; YGR160W Protein of unknown function
YGR179C; G7144; OKP1 Component of centromere/kinetochore-associated protein network
HGH1; G7538; YGR187C Protein with similarity to human HMG1 and HMG2 proteins
SER2; LST3; PSP G7744; YGR208W Phosphoserine phosphatase involved in synthesis of serine from 3-phosphoglycerate
PRS3; SIW17; YHL011C; PRPS3 Phosphoribosylpyrophosphate synthetase (ribose-phosphate pyrophosphokinase II) component of yeast 20Sproteasome, with a role in cell cycle regulation
RSC30; YHR056C Protein of unknown function, encoded from the CUP1A and CUP1B repeat region
YHR085W Protein of unknown function
HXT4; LGT1; RAG1; YHR092C Moderate- to low-affinity hexose transporter, member of the hexose transporter family of the major facilitatorsuperfamily (MFS)
HXT1; YHR094C Low-affinity hexose transporter, member of the hexose transporter family of the major facilitator superfamily (MFS), induced byglucose only at high concentration
MSH1; YHR120W Protein involved in mitochondrial DNA repair homolog of E. coli MutS
DBP8; YHR169W Protein with similarity to DEAD box family of RNA helicases

YHR196W Protein of unknown function
HIS6; YI3299.11; YIL020C Phosphoribosyl imidazolecarboxamide isomerase
KTR7; YI9910.11; YIL085C Putative mannosyltransferase of the KRE2 family
PAN6; YIL145C Protein required for synthesis of pantothenic acid, has similarity to E. coli pantoate beta-alanine ligase
YIL175W Protein of unknown function, putative pseudogene
YJL109C; J0808 Protein with weak similarity to Drs2p
GEF1; STP24; CLCY1; (CLC1); GTC779; J1616; YJR040W Voltage-gated chloride channel, has effects on intracellular iron metabolism
YJR041C; GTF1174; J1622 Protein with similarity to Podospora anserina NADH dehydrogenase chain 4
NUP85; RAT9; GTC744; J1624; YJR042W Nuclear pore protein
HAM1; J1811; YJR069C Protein controlling 6-N-hydroxylaminopurine sensitivity and mutagenesis
NMD5; J2112; KAP119; YJR132W Member of the karyopherin-beta family involved in nuclear transport
MRT4; YKL160; YKL009W Protein involved in mRNA turnover
YKL027W Protein with similarity to E. coli molybdopterin-converting factor
MAE1; YKL029C Mitochondrial malate dehydrogenase (NADP+, oxaloacetate-decarboxylating)
YKL030W; (YKL243) Protein of unknown function questionable ORF
JA2; DHR2; YKL408; YKL078W RNA helicase of DEAH box family, possible pre-mRNA splicing factor
YKL099C; YKL449 Protein of unknown function
YKL102C; YKL454 Protein of unknown function
AAT1; YKL461; YKL106W Putative aspartate aminotransferase, mitochondrial
OAC1; PMT (PMT1); YKL522; YKL120W Mitochondrial oxaloacetate transporter, member of the mitochondrial carrier (MCF) family
RRN3; YKL125W RNA polymerase I transcription factor
EBP2; YKL636; YKL172W Protein involved in pre-rRNA processing and ribosomal subunit assembly
DPH2; YKL191W Diptheria toxin resistance protein, required for diphthamide biosynthesis
RPC37; YKR025W RNA polymerase III, C37 subunit
GCN3; AAS2; YKR026C Translation initiation factor eIF2B (guanine nucleotide exchange factor), 34 kDa (alpha) subunit
YKR043C Protein with similarity to phosphoglycerate mutase
YKR060W Protein of unknown function
YKR071C Protein of unknown function
YKR079C Protein of unknown function
SRP40; YKR412; YKR092C Nucleolar protein, suppressor of rpc40 and rpb10 mutations
DRS1; L1345; YLL008W RNA helicase of the DEAD box family involved in rRNA biogenesis
YLL044W; L0725 Protein of unknown function
YLR002C; L1392 Protein of unknown function
YLR022C; L1722 Protein of unknown function
YLR065C; L2180 Protein of unknown function
BUD20; L2337; YLR074C Protein of unknown function, has a single C2H2-type zinc finger
DIP2; L9233.1; L3116; YLR129W Dom34p-interacting protein, has WD (WD-40) repeats
SPE4; L9634.5; L3305; YLR146C Spermine synthase (spermidine aminopropyltransferase), catalyzes the final step in polyamine biosynthesis,required for biosynthesis of pantothenic acid
ASP3B; (ASP3); L9632.7; YLR157C L-asparaginase II, extracellular
YLR186W; L9470.5 Protein of unknown function
SIK1; NOP56; L8167.9; YLR197W Nucleolar protein component of box C/D snoRNPs, which are necessary for 2'-O-methylation of ribosomalRNAs
YLR198C Protein of unknown function
YLR222C; CST29; L8083.8 Protein of unknown function, overproduction causes chromosome instability and increased mitotic recombination,contains WD (WD-40) repeats
THI7; THI10; UPL3; L8083.2; YLR237W Thiamine transport protein
DBP9; L9328.3; YLR276C Protein with similarity to DEAD box RNA helicases
GSP1; CNR1; RAN CST17; L8003.19; YLR293C Ran, a GTP-binding protein of the ras superfamily involved in trafficking through nuclear pores

EXG1; SCW6; BGL1; L8003.3; YLR300W Exo-beta-1,3-glucanase (I/II) major isoform involved in cell wall beta-glucan assembly
FKS1; GLS1; CWH53; ETG1; CND1; GSC1; PBR1; PBS1; L8300.6; YLR342W Component of beta-1,3-glucan synthase, probably functions asan alternate subunit with Gsc2p with which it has strong similarity
YLR401C; L8084.19 Protein with weak similarity to R. capsulatus protein nifR3
YLR405W; L8084.2 Protein of unknown function
YLR408C; L8084.21 Protein of unknown function
YLR409C; L8084.22 Protein of unknown function, has WD (WD-40) repeats
IMD3; L9753.4; YLR432W Protein highly similar to Imd2p and inosine-5'-monophosphate dehydrogenase of human and E. coli
YLR435W; L9753.3 Protein of unknown function, may be involved with protein synthesis
YML014W; YM9571.04 Protein with similarity to Diptheria toxin resistance protein
YML018C Protein of unknown function
IMD4; YM9958.06; YML056C Protein with similarity to inosine-5'-monophosphate dehydrogenase
ERB1; YM9796.02; YMR049C Protein with possible role in ribosome biogenesis, has similarity to mouse Bop1 growth supressor, has one WD(WD-40) domain
YMR102C Protein of unknown function, contains WD (WD-40) repeats
ILV2; SMR1; YM9718.07; YMR108W Acetolactate synthase (acetohydroxyacid synthase), first step in valine and isoleucine biosynthesispathway
PKR1; YM8564.05; YMR123W Protein of unknown function
GUA1; YM8261.11; YMR217W GMP synthetase, catalyzes the amination of xanthine monophosphate to guanine monophosphate in the guaninebranch of the purine biosynthesis pathway
RRN9; YM8156.12; YMR270C Component of the Upstream Activation Factor (UAF) complex, involved in activation of RNA polymerase I
NIP1; YMR309C Subunit of translation initiation complex eIF3, also required for nuclear import has weak similarity to Nsr1p
BOP3; N2670; YNL042W Protein of unknown function
YNL110C; N1954 Protein of unknown function, contains one RNA recognition (RRM) domain
RPC19; N1937; YNL113W Shared subunit of RNA polymerases I and III
YNL114C; N1934 Protein of unknown function, questionable ORF
YNL174W; N1669 Protein of unknown function
POP1; N1285; YNL221C Protein component of both RNase P and RNase MRP, involved in both tRNA maturation (RNase P) and in 5.8S rRNAprocessing (RNase MRP)
POP3; N0586; YNL282W Component of both RNase MRP and RNase P, involved in processing of both rRNA and tRNA precursors
TRF5; YNL0440; N0440; YNL299W DNA polymerase kappa, required for sister chromatid cohesion
KRI1; N0388; YNL308C Protein required for synthesis of 40S ribosomal subunits, binds Krr1p and has weak similarity to Mgm1p
URK1; N2050; YNR012W Uridine kinase, converts ATP and uridine to ADP and UMP
YNR013C; N2052 Protein with similarity to Pho87p and Yjl198p, member of the phosphate permease family of membrane transporters
YNR020C; N3212 Protein of unknown function
BIO4; N3506; YNR057C Dethiobiotin synthase, component of the biotin biosynthesis pathway
RCL1; O2357; YOL010W Protein required for pre-rRNA processing at cleavage sites A0, A1 and A2, has similarity to RNA 3'-terminal phosphatecyclase
SCM2; TAT2; TAP2; LTG3; SAB2; O2301; YOL020W High affinity tryptophan permease, also transports other aromatic amino acids, alanineand glycine
YOL037C; O2101 Protein of unknown function
PRS5; PRPS5; O1213; YOL061W Phosphoribosylpyrophosphate synthetase (ribose-phosphate pyrophosphokinase)
YOR004W; UNB254; O2521 Protein of unknown function
STD1; MSN3; SFS3; O2777; YOR047C Dosage-dependent modulator of glucose repression increased dosage restores invertase expression insnf4 mutants
YOR051C; YOR29-02; O2793 Nuclear protein of unknown function
RGS2; YOR3224; O3224; YOR107W Negative regulator of glucose-induced cAMP signaling pathway
ORT1; ARG11; O3299; YOR3299; YOR130C Ornithine transport protein of mitochondria involved in arginine metabolism, member of themitochondrial carrier (MCF) family

YOR169C; O3605 Protein of unknown function
YOR203W; O4833 Protein of unknown function
YOR206W; O4843; YOX001 Protein of unknown function
RPB8; YOR50-14; O5014; O5070; YOR224C Shared subunit of RNA polymerases I, II, and III (ABC14.5)
YOR359W; O6638 Protein of unknown function
YPL170W; P2515 Protein with similarity to steroid membrane receptors
PUS1; P1805; YPL212C Pseudouridine synthase responsible for specific pseudouridine modifications in tRNA and in U2 snRNA
NEW1; P1445; YPL226W Member of the non-transporter group of the ATP-binding cassette (ABC) superfamily
YPL238C; P1051 Protein of unknown function
ARO7; OSM2; HGS1; YP9499.15; YPR060C Chorismate mutase, required for the synthesis of both phenylalanine and tyrosine
YPR143W; P9659.4 Protein of unknown function
RPC82; RPC3; P9677.11; YPR190C RNA polymerase III, third-largest subunit
RPC10; RPB12; YHR143W-A Shared subunit of RNA polymerases I, II, and III (ABC10alpha), has zinc-binding domain

Supplementary Table S6:
Homologous genes which are induced in Sporulation or Return to Growth

Homologous genes (that were found using systematic blast that was done in our laboratory) were checked for their expression during sporulation and Return to Growth (RTG). The genes were classified according to their induction (during sporulation or RTG). The function of the genes is shown on the left. Genes marked with asterisk are induced both during sporulation and RTG.

**Function**	**Sporulation**	**RTG**
Amino acid transport	*ALP1*	*TAT2*
Cell wall	*CRR1*	*UTR2*
Cell wall organization and biogenesis (Exo-beta-1,3-glucanase)	*EXG2*	*EXG1*
Cell wall/spore wall	*SPS100*	*ASP3-2*
Chromatin silencing	*ORC1*	*SIR3*
Chromosome condensation	*SMC3*	*SMC4*
DNA repair	*MSH4,5,6*	*MSH1*
recombination	*MEK1*	*DUN1*[*]
Exo-beta-1,3-glucanase	*FKS3*	*FKS1*
Intracellular transport	*VPS21*	*GSP1*
Kinase	*MEK1,SMK1, RIM11, MRK1*	*CKA2*
Meiosis	*ZIP1*	*BFR1*[*]
Nucleocytoplasmic transport	*CSE1*	*SXM1, NMD5*
Phosphatase	*DIA3*	*PHO3*
Phospholipid transport	*PDR17*	*PDR16*
protein amino acid glycosylation	*SRT1*	*RER2*
septin	*SPR3, CDC3,CDC10*	*CDC12*[*]
snRNA-nucleus export	*NUP116*	*NUP100, NSP1*
Transcription factor	*USV1*	*MIG3*
Transport, mitochondrial	*ODC1, PET8*	*GGC1, OAC1, ARG11*

Supplementary Table S7: Middle Sporulation genes that are repressed upon transfer to YPD

The average pattern (log_2ratio) is plotted upon transfer to YPD (which was followed for 5, 20 and 40 minutes after the transfer). The genes in the group, along with a one line description, are indicated.
Template genes used to create this group are: *CDC20, YDL114W and YNL319W*.

Group	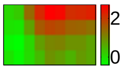
YAL018C; YAL003 Protein of unknown function, has 5 potential transmembrane segments	
PAU7; YARS03C; YAR020C Member of the seripauperin (PAU) family, has strong similarity to Pau3p/Ycr104p	
CDC27; SNB1; YBL0718; YBL084C Component of anaphase-promoting complex (APC), required for the metaphase-anaphase transition and forClb2p degradation	
GAL7; YBR0226; YBR018C UDP-glucose--hexose-1-phosphate uridylyltransferase	
TCM62; YBR0414; YBR044C Chaperone-like protein required for the assembly of the mitochondrial succinate dehydrogenase complex	
GIP1; YBR0420; YBR045C Glc7p-interacting protein, possible regulatory subunit for the PP1 family protein phosphatases	
YBR063C; YBR0610 Protein with similarity to phosphopanthethein-binding proteins	
YBR064W; YBR0612 Protein of unknown function	
YBR071W; YBR0712 Protein with weak similarity to Herpesvirus saimiri EERF2	
IML3; MCM19; YBR0836; YBR107C Protein required for minichromosome maintenance	
UBS1; YBR1217; YBR165W Positive regulator of Cdc34p	
YBR168W; YBR1220 Essential protein of unknown function	
DTR1; YBR1242; YBR180W Dityrosine transporter, member of the multidrug-resistance 12-spanner (DHA12) family of the major facilitatorsuperfamily (MFS-MDR)	
FTH1; YBR1448; YBR207W Vacuolar iron transporter with similarity to Ftr1p	
YBR250W; YBR1702 Protein of unknown function	
YPT10; YBR1733; YBR264C Protein member of the rab family in the ras superfamily of small GTP-binding proteins	
MRPL37; YML37; YBR1736A; YBR268W Mitochondrial ribosomal protein of the large subunit	
YCL010C Protein of unknown function	
YCL048W Protein with similarity to Ecm33p and Sps2p	
CDC10; YCR022; YCR002C Septin, component of 10 nm filaments of mother-bud neck, involved in cytokinesis	
YCR025C; YCR244 Protein of unknown function	
YCR033W; YCR592 Protein of unknown function	
YCR045C Protein with similarity to protease B (Prb1p) and subtilisin family proteases	
YCR086W; SPO86 Protein possibly involved in sporulation	
APC11; D2900; YDL008W Component of the anaphase-promoting complex (APC), has ubiquitin ligase activity and is required for themetaphase-anaphase transition	
MPS1; RPK1; PAC8; D2785; YDL028C Serine/threonine/tyrosine protein kinase involved in spindle pole body duplication and in mitoticcheckpoint	
QRI1; UAP1; D2362; YDL103C UDP-N-acetylglucosamine pyrophosphorylase	
YDL114W; D2297 Protein with similarity to proteins of the short-chain alcohol dehydrogenase family	
CLB3; D1539; YDL155W G2/M-phase-specific cyclin	
YDL186W; D1281 Protein of unknown function	
YDL239C; D0771 Putative coiled-coil protein, has similarity to S. cerevisiae Cnm67p, a spindle pole body and nuclear migration protein	
YDL247W; MPH2; D0220 Protein with similarity to maltose permeases including Yjr160p, Mal31p, Mal61p, member of the hexose transporter familyof the major facilitator superfamily (MFS)	
YDR010C; PZA109; PZE110; D3209; YD8119.15 Protein of unknown function	
GIS1; YD8557.03; YDR096W DNA damage-responsive repressor of PHR1, has two tandem	

C2H2-type zinc finger domains
STE5; NUL3; YD8557.12; YDR103W Pheromone pathway scaffolding protein involved in a complex with Fus3p, Ste7p, and Ste11p
SPO71; YD8557.13; YDR104C Protein involved in spore wall formation
APC4; YD9727.13; YDR118W Component of the anaphase-promoting complex (APC), required for Clb2p degradation and for themetaphase-anaphase transition
EKI1; YD8358.04; YDR147W Ethanolamine kinase I has similarity to Cki1p choline kinase
UBC1; YD9395.10; YDR177W Ubiquitin conjugating enzyme (E2) that plays a role early in spore germination
YDR179C; YD9395.12 Protein of unknown function
PLP1; YD9395.17; YDR183W Phosducin homologue likely to be involved in regulation of pheromone response
YDR196C; YD9346.07 Protein of unknown function
SPR28; YD9934.03; YDR218C Septin-related protein expressed during sporulation
EXG2; YD9320A.12; YDR261C Exo-beta-1,3-glucanase (beta-1,3-D-glucanglucanohydrolase), minor isoform
DIN7; DIN3; YD9320B.02; YDR263C Mitochondrial inner membrane nuclease with a role in stabilizing the mitochondrial genome expression isinduced by DNA damage
CCC2; D9954.6; YDR270W Copper-transporting P-type ATPase, member of the cation transport (E1-E2) ATPase family
YDR273W; DON1; D9954.4 Component of the prospore membrane
TFB1; D9740.3; YDR311W Component of RNA polymerase II transcription initiation factor TFIIH (factor b), 75 kDa subunit
YDR317W; D9798.8 Protein of unknown function
YDR371W; D9481.7 Protein with similarity to chitinases
DIT2; CYP56; D9509.20; YDR402C Cytochrome P450 56, second enzyme in the pathway for biosynthesis of dityrosine in the outer layer of thespore wall
PDR15; D9509.24; YDR406W Member of ATP-binding cassette (ABC) superfamily
TRS120; D9509.25; YDR407C Protein of the TRAPP (TRAnsport Protein Particle) complex involved in vesicular transport from endoplasmicreticulum to Golgi
YDR438W; (D9461.24) Protein of unknown function
DIG2; (RST2); D8035.23; YDR480W MAP kinase-associated protein involved in negative regulation of invasive growth and mating response
PAC11; D8035.31; YDR488C Dynein intermediate chain, required in the absence of Cin8p, member of the WD (WD-40) repeat family
LCD1; DDC2; CIP1; PIE1; D9719.5; YDR499W Essential protein required for DNA damage checkpoint pathway in G1 and G2/M phases of cellcycle
YDR504C; D9719.10 Protein of unknown function
PAD1; POF1; D9719.39B; YDR538W Phenylacrylic acid decarboxylase
YDR539W; D3703.2 Protein of unknown function
YEL016C Protein with similarity to Ycr026p
YEL017W Protein of unknown function
YEL023C Protein of unknown function
PCM1; AGM1; YEL058W Hexosephosphate mutase (phosphoacetylglucosamine mutase, N-acetylglucosamine-phosphate mutase) convertsN-acetyl-D-glucosamine 1-phosphate to N-acetyl-D-glucosamine 6-phosphate
CIN8; KSL2; SLN2; SDS15; CST16; YEL061C Kinesin-related protein involved in establishment and maintenance of mitotic spindle
SPC25; YER018C Component of the Tid3p-Nuf2p-Spc24p-Spc25p centromere complex, involved in chromosome segregation
YER046W Protein of unknown function
YER084W Protein of unknown function
YER085C Protein of unknown function
UBC6; DOA2; YER100W Ubiquitin-conjugating enzyme anchored in the ER membrane with the catalytically active domain in cytoplasm
MAM1; YER106W Monopolin, protein involved in chromosome attachment to the meiotic spindle, induced in sporulation
ISC10; SYGP-ORF62; YER180C Meiosis-specific protein required for spore formation

YER182W Protein of unknown function
HXT10; YFL011W Hexose transporter, member of the hexose transporter family of the major facilitator superfamily (MFS)
GNA1; (PAT1); YFL017C Phosphoglucosamine acetyltransferase
YFL040W Putative hexose transporter, member of the hexose transport family of the major facilitator superfamily (MFS)
FET5; YFL041W Multicopper oxidase involved in ferrous iron transport
YFR012W Protein of unknown function
PES4; YFR023W Suppressor of DNA polymerase epsilon mutation, contains four RNA recognition motif (RRM) domains
CDC14; OAF3; YFR028C Dual specificity protein phosphatase (PTPase) that performs a function late in the cell cycle
CDC26; HIT3; SCD26; YFR036W Component of the anaphase-promoting complex (APC), required for Clb2p degradation and for themetaphase-anaphase transition
CDH1; HCT1; G3765; YGL003C Protein involved in Clb2p cyclin proteolysis, has WD (WD-40) repeats
YGL015C; YGL026; G3716 Protein of unknown function
CDC20; PAC5; G2939; YGL116W Activator of anaphase promoting complex (APC), required for microtubule function at mitosis and for exit fromanaphase, contains WD (WD-40) repeats
NAB2; G2910; YGL122C Nuclear poly(A)-binding protein, required for proper polyadenylation of pre-mRNA and for mRNA export
SUT1; G1828; YGL162W Protein involved in sterol uptake
YGL170C; G1654 Protein with similarity to phosphoribulokinase precursor
YGL176C; G1636 Protein with weak similarity to Discopyge ommata Ca2+ channel alpha1 subunit protein B47447
PDE1; NRB369; G0574; YGL248W 3',5'-Cyclic-nucleotide phosphodiesterase, low affinity
SPR3; G4353; YGR059W Sporulation-specific septin
CLB1; SCB1; G5967; YGR108W G2/M-phase-specific cyclin
CLB6; G5970; YGR109C B-type cyclin appearing late in G1, involved in initiation of DNA synthesis
TOS10; G6661; YGR153W Protein of unknown function
FYV8; G7589; YGR196C Protein of unknown function
YGR201C; G7727 Protein with similarity to translation elongation factors
AMA1; ORF409; SPO70; G8541; YGR225W Protein required for proper meiosis and sporulation, associates with and regulates the activity ofanaphase promoting complex/cyclosome (APC/C), member of Cdc20 family of proteins
YGR273C; G9371 Protein of unknown function
RNH70; G9381; YGR276C Ribonuclease H (70 kDa), endonuclease that degrades RNA in RNA-DNA hybrids
YHL012W Protein with similarity to UTP glucose-1-phosphate uridylyltransferase
WSC4; YHC8; YFW1; YHL028W Protein required for secretory protein translocation, for maintenance of cell wall integrity, and for the stressresponse
MIP6; YHR015W Protein with similarity to Pes4p and Pab1p in the N-terminal region, has 4 RNA recognition (RRM) domains
EPT1; YHR123W sn-1,2-Diacylglycerol ethanolaminephosphotransferase, catalyzes the synthesis of phosphatidylethanolamine fromCDP-ethanolamine and diacylglycerol
NDT80; YHR124W Meiosis-specific protein required for exit from pachytene
WSS1; YHR134W Protein of unknown function
YHR150W Protein of unknown function
YHR151C Protein of unknown function
CDC23; H9986.19; YHR166C Component of anaphase-promoting complex (APC) required for Clb2p degradation and for the metaphase-anaphasetransition
SSP1; YHR184W Meiosis-specific protein, required for proper completion of meiotic division and spore formation
ADY1; YHR185C Protein required for sporulation
PIG2; YI9905.03; YIL045W Protein that interacts with Gsy2p, possible regulatory subunit for the PP1-family protein phosphatase Glc7p
FIS1; MDV2; YIL065C Protein involved in mitochondrial division

YIL112W Protein with similarity to ankyrin and coiled-coil proteins
ECM37; YIL146C Protein possibly involved in cell wall structure or biosynthesis
DNA43; MCM10; YIL150C Protein required for S-phase initiation or completion
YIL154C; (IMP2); BLM25; HCY39 Protein involved in control of mitochondrial sugar utilization and repair of oxidative DNA lesions
BNR1; YIL159W Bni1p-related protein, potential target of Rho4p
SDL1; (SED1); (SDH1); YI9402.08A; YI9402.08B; YIL167W; YIL168W Serine dehydratase, converts serine to pyruvate and ammonia forgluconeogenesis
MAD3; J1341; YJL013C Spindle-assembly checkpoint protein required for cell cycle delay in response to impaired kinetochore function
YJL038C; J1232 Protein of unknown function
YJL043W; J1204 Protein of unknown function
CHS6; CSD3; J0838; YJL099W Protein involved in chitin synthase Chs3p activity has tetratricopeptide (TPR) repeats , mutants are resistant tocalcofluor white
YJL131C; J0682 Protein with similarity to carbamylphosphate synthetase
IDS2; J0642; YJL146W Protein that affects the function of Ime2p
YJL160C; J0555 Protein with similarity to members of the Pir1p/Hsp150p/Pir3p family
YJL162C; J0549 Protein with similarity to E. coli DnaJ and other DnaJ-like proteins
PET191; GTA108; J1604; YJR034W Protein involved in assembly of cytochrome oxidase
HUL4; HCT4; GTD892; J1608; YJR036C Putative ubiquitin-protein ligase
YJR037W; GTC127; J1610 Protein of unknown function
STE18; J1866; YJR086W Gamma subunit of the guanine nucleotide-binding protein that mediates signal transduction by pheromones duringmating
YUH1; J1941; YJR099W Ubiquitin-specific protease
YJR107W; J1983 Protein of unknown function, has similarity to acylglycerol lipase
YJR141W; J2166 Essential protein of unknown function
YJR160C; MPH3; J2400 Protein with strong similarity to maltose permeases (maltose/H+ symporters) Mal31p and Mal61p, member of hexosetransporter family of the major facilitator superfamily (MFS)
CDC16; YKL022C Component of anaphase-promoting complex (APC), required for Clb2p degradation and for the metaphase-anaphase transition
YKL100C; YKL450 Protein of unknown function
YKL133C Protein of unknown function
APE2; LAP1; YKL611; YKL157W Aminopeptidase II (yscII), plays a nutritional role in releasing leucine from peptides externally cleaved at leucine
HYM1; YKL189W Protein required for sporulation with similarity to Aspergillus nidulans hymA which is involved in conidiophore development
YKL221W; B473 Protein with similarity to mammalian monocarboxylate transporters MCT1 and MCT2, member of the monocarboxylate porter(MCP) family of the major facilitator superfamily (MFS)
ECM9; YK103; YKR004C Protein possibly involved in cell wall structure or biosynthesis
YKR015C Protein of unknown function
YKR100C Protein of unknown function
ORC3; L1365; (OAF1); YLL004W Origin recognition complex (ORC), 62 kDa subunit
SPA2; PEA1; PPF1; L1209; YLL021W Protein involved in cell polarity and cell fusion during mating
YLL047W; L0709 Protein of unknown function
YLR030W; L1759 Protein of unknown function
STU2; L2108; YLR045C Component of the spindle pole body
YLR054C; L2141 Protein of unknown function
YLR072W; L2321 Protein of unknown function
RAX2; L9449.12; YLR084C; L2389 Protein involved in bipolar budding
NYV1; L8004.4; L2531; YLR093C Synaptobrevin (v-SNARE) homolog involved in vacuolar vesicle fusion
IOC2; L8004.6; YLR095C; L2542 Protein of unknown function
APC9; L8004.10; L2710; YLR102C Component of the anaphase-promoting complex (APC) required for Clb2p degradation and for themetaphase-anaphase transition

YLR126C; L9233.12; L3105 Protein with weak similarity to Pseudomonas aeroginosa anthranilate synthase component II
APC2; RSI1; TID2; L9233.13; L3108; YLR127C Component of the anaphase-promoting complex, required for Clb2p degradation and for themetaphase-anaphase transition
PNP1; L8167.19; YLR209C Protein with similarity to human purine nucleoside phosphorylase
CLB4; L8167.3; YLR210W G2/M-phase-specific cyclin
CRR1; L8167.22; YLR213C Sporulation specific protein with similarity to Crh1p cell wall protein
CCC1; L8083.6; YLR220W Protein with potential role in calcium regulation, overproduction suppresses the calcium-sensitive phenotype of csg1strains
CDA1; L2142.2; YLR307W Chitin deacetylase, responsible along with Cda2p for the synthesis of chitosan for the ascospore wall
EST2; L8543.12; YLR318W Catalytic component of telomerase, predicted reverse transcriptase
YLR341W; L8300.7 Protein of unknown function
YLR343W; L8300.5; GAS2 Protein with strong similarity to Gas1p, member of the GPI-anchored beta(1-3)glucanosyltransferases family
ROM2; L8039.3; YLR371W GDP-GTP exchange factor for Rho1p that is activated by cell wall defects
CSR1; SFH2; L3502.4; YLR380W Phosphatidylinositol transfer protein, involved in regulation of phospholipase D (Spo14p) activity
SRC1; YML033W; YML034W Protein of unknown function, expression is cell cycle-regulated
MFT1; MFT52; YML062C Targeting factor for mitochondrial precursor proteins, member of a family of transmembrane transition metal transporters
YML066C Protein of unknown function
YML119W; YM7056.07 Protein of unknown function
CDC5; PKX2; MSD2; YM8270.03; YMR001C Serine/threonine protein kinase required for exit from mitosis may be involved in operation of themitotic spindle, member of the polo family of protein kinases
SPO20; YM9711.05; YMR017W; DBI9 Protein required for sporulation and growth of prospore membrane, interacts with Dbf2p
NUP116; NSP116; YM9532.12; YMR047C Nuclear pore protein (nucleoporin) of the GLFG family may be involved in binding and translocation ofnuclear proteins
STV1; YM9796.07; YMR054W Subunit of V0 sector of V-ATPase proton pump of non-vacuolar organelles
ABF2; CDRP1; HIM1; HM p19-HM mtTFA YM9916.11; YMR072W Abundant mitochondrial DNA-binding protein required for maintenance,transmission and recombination of mitochondrial genome, contains two HMG-box DNA-binding domains
STO1; GCR3; CBC1; CBP80; YM9553.01; YM9564.07; YMR125W Large subunit of the nuclear cap-binding protein complex
HOT1; YM8010.02; YMR172W Protein required with Msn1p for hyperosmotic stress-induced gene expression
YMR184W; YM8010.14 Protein of unknown function
YMR188C; YM8010.18 Probable mitochondrial ribosomal protein, has weak similarity to prokaryotic 30S ribosomal protein S17
YMR253C; YM9920.07 Protein of unknown function, likely membrane protein
YNL013C; N2854 Protein of unknown function, questionable ORF
YNL018C; N2831 Protein of unknown function, nearly identical to Ynl034p
YNL019C; N2827 Protein of unknown function
YNL026W; N2802 Protein of unknown function
YNL033W; N2743 Protein of unknown function
YNL034W; N2740 Protein of unknown function, nearly identical to Ynl018p
YNL080C; N2327 Protein of unknown function
YNL095C; N2215 Protein with similarity to Ecm3p
MET4; N2177; YNL103W Transcriptional activator of the sulfur assimilation pathway member of basic leucine zipper (bZIP) family
SPC98; N122; N1879; YNL126W Spindle pole body component that interacts with gamma-tubulin

YNL170W; N1688 Protein of unknown function, questionable ORF
YNL171C; N1684 Protein of unknown function, questionable ORF
YNL205C; N1350 Protein of unknown function
ALG9; N1295; YNL219C Mannosyltransferase involved in N-glycosylation, catalyzes transfer of mannose from Dol-P-Man to lipid-linkedoligosaccharides
AUT2; APG4; N1274; YNL223W Cysteine endopeptidase involved in autophagy, required for attachment of autophagosomes to microtubules,also required for sporulation in combination with Aut7p
CNM67; CNM1; N1264; YNL225C Protein involved in nuclear migration and component of the spindle pole body
ALP1; (APL1); N0660; YNL270C Arginine permease with strong similarity to basic amino acid permeases Can1p and Lyp1p
HXT14; N0345; N0344; YNL318C Member of the hexose transporter family of the major facilitator superfamily [MFS]
YNL319W; N0343 Protein of unknown function, questionable ORF
AUT1; N2040; YNR007C Protein involved in autophagocytosis during starvation
YNR068C; N3551 Protein with similarity to Bul1p ubiquitin ligase binding protein, may be a pseudogene or separated from YNR069C bysequencing errors
YOL015W; O2329 Protein of unknown function, induced during anaerobic growth
YOL024W; O2181 Protein of unknown function
SPO21; MPC70; O0932; YOL091W Component of the meiotic plaque, required for formation of the prospore membrane
MED7; AOE222; O0505; YOL135C Component of RNA polymerase II holoenzyme and mediator subcomplex
YOL146W; AOA236; O0456 Protein of unknown function
EXO1; DHS1; O2727; OR26.23; YOR033C Double-stranded DNA 5'->3' exonuclease, involved in mismatch repair and recombination
YOR073W; YOR29-24; O2945 Protein of unknown function
PTC5; O3157; YOR3157; YOR090C Putative serine/threonine protein phosphatase of the PP2C family
ARF3; ARL2; YOR3172; O3172; YOR094W Protein with similarity to members of the arf family (ras superfamily) of GTP-binding proteins
IAH1; (EST2); O3287; YOR126C Isoamyl acetate-hydrolyzing esterase enzyme
YOR152C; O3536 Protein of unknown function
DCI1; ECI2; EHD2; EDH2; O4718; YOR180C Peroxisomal dienoyl-CoA isomerase
MSB1; O4744; YOR188W Protein that may play a role in polarity establishment and bud formation
STE4; HMD2; YOR50-2; O5002; YOR212W Beta subunit of the trimeric G protein that mediates signal transduction by pheromones, member ofthe WD (WD-40) repeat family
YOR218C; YOR50-8; O5008; O5042 Protein of unknown function
HES1; OSH5; O5234; YOR237W Protein implicated in ergosterol biosynthesis, member of the KES1/HES1/OSH1/YKR003W family ofoxysterol-binding (OSBP) proteins
APC5; O5280; YOR249C Component of the anaphase-promoting complex (APC), required for Clb2p degradation and for the metaphase-anaphasetransition
YOR251C; O5305 Rhodanese (thiosulfate sulfurtransferase), functions to detoxify cyanide
YOR255W; O5325 Protein of unknown function
HEM4; SLU2; CYT TS2326; O5463W; YOR278W Uroporphyrinogen III synthase, fourth step in heme biosynthesis pathway
YOR282W; O5477 Protein of unknown function
TIM18; O5627; YOR297C Component of the inner mitochondrial membrane translocation complex
RAX1; O5642; YOR301W Protein involved in determination of budding pattern
YOR305W; O5653 Protein of unknown function
UBC11; O6268; YOR339C Protein with similarity to ubiquitin-conjugating enzymes
YOR345C; O6336 Protein of unknown function
CIN1; O6350; YOR349W Protein involved in chromosome segregation, required for microtubule stability
YOR352W; O6608 Protein of unknown function

YOR365C; O6661 Protein of unknown function
ATF1; O6741; YOR377W Alcohol acetyltransferase, catalyzes condensation of acetyl-CoA to various alcohols to produce acetate esters
FRE3; O6754; YOR381W Protein involved in the uptake of iron from siderophores rhodotorulic acid, triacetylfusarinine C, ferrichrome andferrioximine B
SRD2; ECM23; LPB10; YPL021W Protein with similarity to Srd1p
YPL027W; LPB4 Protein of unknown function
YPL047W; P7102.04 Protein of unknown function
ARL3; (ARP1); LPE21; YPL051W ADP-ribosylation factor-like protein, member of the arf-sar family in the ras superfamily
BEM3; LPH12; YPL115C GTPase-activating protein (GAP) for Cdc42p and Rho1p, contains a PH (pleckstrin) domain
CBC2; CBP20; MUD13; SAE1; P2259; YPL178W Small subunit of nuclear cap-binding protein complex
DDC1; P1920; YPL194W DNA damage checkpoint protein, delays the cell cycle at the G1/S boundary in response to UV irradiation, member of theRAD24 epistasis group
YPL267W; P0360 Protein of unknown function
YPR027C; YP9367.07 Protein with similarity to Ynl019p and Ynl033p
SMK1; YP9499.10; YPR054W Sporulation-specific protein kinase of the MAP family required for completion of sporulation
YPR078C; P9513.10 Protein of unknown function
YPR079W; P9513.8 Protein of unknown function
CLB5; P9642.8; YPR120C B-type cyclin appearing late in G1, involved in initiation of DNA synthesis
KAR3; OSR11; P9659.16; YPR141C Kinesin-like protein involved in mitosis and essential for the congression (nuclear migration) step ofkaryogamy, probable coiled-coil protein

Supplementary Table S8: Middle sporulation genes that are insulated

The genes in the insulated group. The average pattern (log_2ratio) is plotted upon transfer to YPD (which was followed for 5, 20 and 40 minutes after the transfer). The genes in the group, along with a one line description, are indicated.
Template genes used to create this group are: *SGA1, SPR6, YDR042C* and *YPL130W*.

Insulated genes

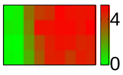

YCL048W Protein with similarity to Ecm33p and Sps2p
YDR042C; YD6888.04; D4155 Protein of unknown function
YDR371W; D9481.7 Protein with similarity to chitinases
YDR516C; D9719.21 Protein with similarity to Glk1p
SPS2; D9719.26; YDR522C Protein involved in middle/late stage of sporulation
SPS1; D9719.27; YDR523C Serine/threonine protein kinase involved in middle/late stage of meiosis
SPR6; YER115C Sporulation-specific protein
HXT10; YFL011W Hexose transporter, member of the hexose transporter family of the major facilitator superfamily (MFS)
YFR032C Protein with weak similarity to S. pombe poly(A)-binding protein (SP:P31209), has one RNA recognition (RRM) domain
YGL230C; G0946 Protein of unknown function
SGA1; SGA SAG YIL099W Glucoamylase (glucan-alpha-1,4-glucosidase), sporulation-specific
GAT4; YIR013C Putative GATA zinc finger transcription factor
YJL037W; J1234 Protein of unknown function
YLR049C; L2121 Protein of unknown function
YNL155W; N1751 Protein of unknown function
SOL1; N3291; YNR034W Possible 6-phosphogluconolactonase involved in tRNA synthesis and function
YOL132W; AOA471; O0514; GAS4 Protein with similarity to glycophospholipid-anchored surface glycoprotein GAS1
VPS21; VPT21; YPT51; YOR3154; O3154; YOR089C GTP-binding protein required for sorting of vacuolar proteins and involved in late stage ofendocytosis, member of the rab family in the ras superfamily
SPR1; SSG1; SSG O4750; YOR190W Sporulation-specific exo-beta-1,3-glucanase, involved in late sporulation
YOR214C; YOR50-4; O5004 Protein of unknown function
SSP2; O5251; YOR242C Sporulation-specific protein
SPS4; O6120; YOR313C Protein expressed in mid-late (8-14 hr) sporulation, possible cell wall component
SPO19; LPI15; YPL130W Sporulation specific protein, putative glycophosphatidylinositol (GPI)-anchored spore wall protein
YPR077C; P9513.9B Protein of unknown function

Supplementary Table S9: List of 936 genes used for Figure 6a

YJL162C	PDS5	YER146W	YKL172W	YMR123W
YCL002C	YPR194C	YOR318C	YOR004W	YBL054W
CLB4	CLB2	AST1	YLR022C	DIS3
RTG1	PGD1	YPL038W	YNL110C	TRP3
CLB6	DBF4	YDR472W	YLR409C	YNL174W
KAR3	RRP43	ALG5	RPB8	YML093W
ORC3	YHR154W	SEN34	YOL010W	DIP2
SPO16	YGR198W	RAD27	YKL120W	URK1
YDR147W	RRN6	YAP7	HGH1	POP3
BBP1	YOL031C	YHR052W	YDL060W	YLR073C
CIN8	YBR266C	YHR020W	SRO9	YER081W
YBL009W	RED1	RPC40	YGR103W	YLR222C
CDC14	YMR117C	DBP3	HIS4	YOL037C
YPL267W	PRO1	YOR294W	TYS1	YIL145C
PDS1	SCJ1	KTI12	FRS2	YOR222W
BNR1	YDL074C	HO	YLR186W	YMR259C
YDL187C	HTB2	YLR449W	YCR072C	YLR146C
YLR084C	YOR315W	YOR309C	YLA1	SKT5
HST1	YGL218W	YLR413W	YDR398W	YNL227C
SPA2	YMR215W	YAR075W	URA7	YEL048C
YGL183C	YLR183C	YER156C	MAK16	ATF2
HST3	YDL062W	GLY1	EMP70	YDR324C
YOR032C	YHR217C	YER049W	YDR152W	YLR285W
CLB3	MEP2	YOR145C	ZRC1	SSF2
YOR305W	TEL2	YDR465C	RPA190	YGL211W
YLL012W	YOL155C	YPL146C	YER073W	YHR048W
YJL218W	YPL034W	YAL036C	YMR049C	IFH1
YGR242W	YHR185C	SIK1	NIP1	YBR002C
YDL186W	SSU72	YGR145W	DPH2	DRS1
YNL033W	RCS1	YML056C	YHR085W	YKL099C
YPR078C	CDC21	YPL012W	ILS1	GNP1
YBR063C	GPI8	YOR206W	YDR083W	YDR222W
STU2	SEC14	YER082C	YPR157W	YLR407W
YNL095C	YLR018C	YHR196W	YDR449C	YPL252C
DHS1	YPL044C	ROK1	ECM1	FEN1
YDR317W	YJR141W	NOP4	DBP9	TRP4
MED7	SKS1	CPA2	YLL034C	SMI1
YNL319W	YLR226W	RPA43	MPP10	THI80
YOR339C	YIL079C	SUR4	MTR4	YMR285C
YOR301W	YCR043C	ASP1	ADE5,7	YGR035C
YOR073W	YOR183W	FMI1	YIR035C	UBP1
SUT1	YJL098W	PRM7	YGR272C	DUT1
YOR365C	SGD1	PAB1	BAP3	SEC12
CDC5	YML125C	YTM1	YOL014W	YBL042C
CLB1	HIS6	PUS1	YJL200C	YIL064W
YBR152W	KEX2	LCP5	YHR081W	YJL118W
YOL047C	YOL160W	YHR148W	YKR025W	ARG11
YFR012W	YOL007C	SQT1	CLN3	MMT1
YOR029W	KAR1	YMR223W	YDL037C	YOR013W
YNL034W	YOR247W	STD1	YKR079C	STE20
YPR027C	SOK2	YKL029C	NRP1	RPC82
YLR437C	YAR064W	YOR051C	YDR045C	ENP1
YPL141C	SEC72	YNL065W	TRF5	MSH1

YLR074C	YIL007C	YIR036C	WTM1	YMR181C
YBR267W	YPL134C	YDR032C	YGR111W	YHR033W
YKL078W	YBR269C	YBR139W	YPR127W	YGR194C
YJR124C	YDL199C	YJL045W	MRF1'	YSA1
RPC19	SDH4	TTR1	PRD1	YCR076C
YDR299W	ARO9	YAL060W	YPL186C	YOR084W
YDR365C	SIP4	YOL153C	YNL195C	PHB2
YAR1	YDR313C	GIP2	YKL035W	YPK2
YMR014W	YOR273C	YOR289W	GDH2	YER079W
YKR043C	SNC1	HSP78	LAP3	HEM15
HAM1	YBL048W	YOR215C	SSE2	YDR287W
YLR412W	RIM11	YBR149W	CDC48	YPR098C
YGR160W	ABF2	YGR130C	OPI3	YDR534C
YDR280W	YKL100C	YPL004C	PRE3	SDS22
SER2	YDR202C	YLR327C	PRB1	YDL223C
YPR137W	YTA1	YER024W	YHR138C	GLC3
YJR041C	YFL030W	IKS1	JEN1	MDG1
YKL063C	YMR191W	YPR151C	CIT2	PUT4
YLR401C	YER004W	MDH3	YTA5	YJR008W
YNL114C	YDR380W	YOR019W	ATH1	YGL082W
YDR346C	GLG2	UBC8	YTA2	SIP2
AUR1	YKL026C	YBR280C	YAT1	YBR214W
FUN53	APE2	GPH1	UBC5	MHP1
AFG2	COQ4	CIT1	DDI1	YCR079W
YMR269W	YCL038C	YJR085C	HSP42	YPR150W
AAH1	YDL110C	YCL035C	AFR1	YOL129W
YNB4	APG1	YBL078C	RPN5	UFD1
GCN3	RPN4	YPL230W	COS7	BMH2
LYS4	PRE1	SSA3	NIN1	YPL276W
YNR054C	MEK1	HXT5	GCV1	VAC8
YPL030W	FBP26	YJL066C	YAL061W	YDR246W
YER002W	SEO1	PRC1	IDP3	YOL084W
ADE4	SDH3	YJL021C	PUP1	YPR172W
GCD6	YMR31	YFL006W	PBN1	TPS2
YIR020C	YPL201C	YNL194C	YMR135C	PHB1
ERO1	MRP8	YKR018C	PRE2	PFK26
RRN3	ECM37	YBR230C	YGR243W	GDI1
YMC2	YSW1	SBA1	ACH1	YIL087C
VPS9	FUM1	MDH1	YLR345W	YHL021C
YJL212C	YPL222W	GUT1	YHR171W	YOR042W
YCR016W	YPL113C	SUN2	YDR003W	YNL274C
FAL1	YJL068C	HXK1	YCL033C	YCL039W
YLR179C	POR1	YLR205C	YIL124W	ARP2
YOR246C	GAC1	FUN19	YFR044C	PEX3
YNR009W	YLR356W	HRD2	SHP1	YJL103C
YNL182C	HSP26	NCE102	FUN34	PEX14
RNC1	COS8	TES1	YDR100W	GPA2
LOS1	PDA1	YOL083W	PUT2	HSP30
LTV1	YNL305C	YFL044C	YGR284C	PYC1
PDR6	YIL073C	YFR017C	YDL238C	CAK1
YOR078W	YPR007C	ICL2	YCP4	YOR163W
YDL121C	PGM2	YBL064C	SCO2	IDH1
YAR062W	LAP4	YDR018C	TPK2	PRE8

YML089C	VPS17	YHR075C	ECM13	YKR065C
YAP1	YBR056W	TPK1	YGR052W	YDR084C
UBP2	YCR010C	YGR080W	YFR003C	YGR205W
YOR035C	PRE10	PUP3	YBR241C	YBR273C
YPT52	MDH2	YAL049C	YJL185C	RVS167
YFR010W	ACR1	YJR149W	YLR219W	YNL208W
FAA2	HSP104	YJL163C	YJR044C	YPR193C
PRE4	YDL204W	YNL200C	YLR070C	SEN3
YPL196W	YJL142C	YNL115C	GPD1	SCL1
YET1	YER067W	YML117W	GPM2	APE3
ADP1	CPR6	YML090W	YOL082W	YAL028W
AMS1	PHO85	YDR512C	YOL087C	SCC3
PEP4	YPR192W	YHR198C	YSC84	YDR031W
ECM4	DOA4	PGS1	YDL091C	YBR125C
YOR100C	ECM29	YIL088C	YNL134C	SRV2
YDL222C	YKL151C	YNL127W	YIL042C	YMR323W
STF2	YKL034W	YDR453C	YLR001C	TIP1
UGA1	YOR223W	YOR161C	TIN1	SHY1
YGR043C	NCA2	UBP13	YDR214W	YLL015W
YDL124W	YDR061W	NAM8	ALD3	YKL187C
SOL4	YGL250W	YFL042C	YPL109C	TFS1
MLS1	GLG1	YGR101W	YLR177W	YGR127W
YGR067C	YBR216C	YIL010W	KNS1	FAT2
YAK1	SNF3	YLL020C	YGL146C	DLD1
YDR070C	YDR231C	COX15	YBR151W	YDL206W
SDH2	DPL1	YOL073C	YGL219C	YDL019C
SIP18	YMR099C	YER096W	YTP1	GSY2
HSP12	INP54	YLR097C	ICL1	YLR270W
YML128C	YIR016W	YML131W	GLO4	YDL072C
YFL043C	YOL053W	PTK2	YMR315W	YPR030W
YIR038C	YHR112C	YBR052C	YLL023C	YDR425W
YGR086C	YCR083W	REG2	CYP2	SDH1
GLK1	YER063W	YGR235C	SEC1	DBI56
OM45	YHR159W	YIL105C	YJL020C	SOD2
YGL037C	MAL33	SPL2	TRX2	UBA1
YOR173W	YKL091C	YDR391C	CMK1	YMR031C
YMR250W	TRR2	YLR201C	PRP12	YHR087W
YHR097C	ERS1	YER121W	PHR1	YKL195W
GSY1	PEX10	YJL003W	CYB2	YLR118C
HYR1	YGR011W	YDR255C	YIL041W	YMR103C
ISF1	MRPL28	YGR161C	YML118W	PAI3
YGL081W	MDR1	YPR083W	FBP1	YOR228C
YKR011C	YDR107C	YDR386W	YGR232W	COX17
YMR110C	YOL060C	YBR016W	YCR068W	GLO2
GLO1	YDR063W	YDR055W	CAP2	YBL047C
DIP5	PRM4	YJL144W	YPT53	PMC1
YBR285W	YKR003W	SCD5	YDL237W	ATP3
PEP12	YJR126C	CTF19	YGL161C	GCY1
GDH3	ERG9	PTP1	YGL121C	DNM1
PCK1	MDS3	GLC8	CBP4	YCR030C
GYP7	CIS1	MRS4	YKR017C	YLR251W
PBI2	YKL105C	YLR004C	HRD3	NDI1
YAR027W	YNL212W	NHX1	YHR140W	SSD1
YGP1	YOR227W	MBR1	ARP3	YDR475C
CPH1	PEP8	YNL045W	YJR080C	YDR358W

YMR027W	YFR024C	HXT6
YBL098W	EST1	NCA3
GND2	YDL025C	YLR423C
YCL042W	YBL095W	YGR212W
TPS1	YLR241W	YBR157C
YIL036W	YDR230W	STB5
TSL1	YMR006C	YGL084C
SRA1	YEL066W	YPL079W
YGR023W	YPR115W	RPL17
STB2	YDR203W	RPS25
YPL100W	SIS1	RPL35A
YJR096W	YMR251W	YBL086C
YDL016C	SSA2	PDC6
TKL2	MDJ1	PCL8
JSN1	YLR193C	RPS24A
COQ6	YDR511W	IRA2
ZWF1	YOR135C	RPLA3
MAP2	YMR030W	THI4
IDP2	PIP2	PMA2
ECM15	YEL033W	RPL47A
CTT1	MSP1	ROD1
YGR026W	YMR007W	YLR297W
XBP1	YMR025W	UBR1
YBL013W	YPR140W	
YDR154C	YMR304W	
ADE16	HXT7	
YBR116C	YLR149C	
GCV2	GSP2	
YPL277C	YEL020C	
UBI4	YBR223C	
PEL1	YKL123W	
YOR121C	YML087C	
YLR164W	GRE2	
ABP1	ADE17	
YLR252W	YBR006W	
YLR109W	VPS15	
YHR004C	YAL055W	
FUN31	YBR033W	
SSH4	YMR009W	
YMR322C	YOL150C	
YBR137W	PYK2	
PIR3	CHA1	
YHR001W	AST2	
YFR011C	YER135C	
RAS2	YBR047W	
YDL086W	YOR087W	
YML029W	ALD6	
PET54	YBR150C	
YDL218W	YPL170W	
OCH1	OYE3	
DAK1	PEX8	
PIM1	FUN14	
VPS13	ENO1	
YIL029C	SPS100	

Supplementary Table S10: rRNA processing genes
The rRNA processing genes are listed. The average pattern (log_2ratio) is plotted upon transfer to YPD (which was followed for 5, 20 and 40 minutes after the transfer). The genes in the group, along with a one line description, are indicated.

Average expression:	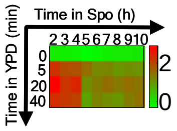
URA7; YBL0410; YBL039C CTP synthase final step in pyrimidine biosynthesis pathway	
KRR1; YCL059C Protein essential for cell division and spore germination	
YDR101C; YD8557.10 Protein of unknown function	
YDR496C; D9719.2 Protein of unknown function	
YER006W Nuclear protein of unknown function, has similarity to mouse Mmr1 protein	
DBP3; G3210; YGL078C ATP-dependent RNA helicase CA3; member of the DEAD/DEAH box family	
ROK1; G1651; YGL171W ATP-dependent RNA helicase required for rRNA processing, member of DEAD box family	
YGR103W; G5933 Nuclear protein of unknown function with similarity to zebrafish pescadillo	
YGR145W; G6623 Component of NuA3 histone acetyltransferase complex	
SDA1; G9101; YGR245C Essential protein required for normal organization of the actin cytoskeleton	
NMD3; SRC5; YHR170W Protein involved in the nuclear export of 60S ribosomal subunits	
YHR197W Component of NuA3 histone acetyltransferase complex	
YKR081C; YKR401 Protein of unknown function	
DRS1; L1345; YLL008W RNA helicase of the DEAD box family involved in rRNA biogenesis	
YLR009W; L1551 Possible ribosomal protein with similarity to ribosomal protein L24	
PWP1; L8167.10; YLR196W Periodic tryptophan protein, has WD (WD-40) repeats	
YLR222C; CST29; L8083.8 Protein of unknown function, overproduction causes chromosome instability and increased mitotic recombination,contains WD (WD-40) repeats	
DBP9; L9328.3; YLR276C Protein with similarity to DEAD box RNA helicases	
YLR409C; L8084.22 Protein of unknown function, has WD (WD-40) repeats	
YML093W Protein of unknown function	
RSA2; YM9553.07; YMR131C Protein involved in ribosome assembly, member of WD (WD-40) repeat family	
HAS1; YMR290C RNA helicase of the DEAD/DEAH box family	
YNL110C; N1954 Protein of unknown function, contains one RNA recognition (RRM) domain	
KRE33; N1216; N1858; YNL132W Protein of unknown function	
YNL174W; N1669 Protein of unknown function	
YNL182C; N1636 Protein of unknown function	
RPA49; N0880; YNL248C RNA polymerase I third largest subunit	
BRX1; O1115; YOL077C Protein required for biogenesis of the 60S ribosomal subunit, localized to the nucleolus	
YOR206W; O4843; YOX001 Protein of unknown function	

Supplementary Table S11: Gluconeogenesis genes

The gluconeogenesis genes are listed. The average pattern (log_2ratio) is plotted upon transfer to YPD (which was followed for 5, 20 and 40 minutes after the transfer). The genes in the group, along with a one line description, are indicated.

Average expression:

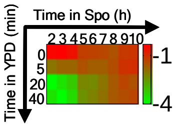

SPG2; YCR010C Protein of unknown function, has strong similarity to Ydr384p and Ynr002p
ICL1; YER065C Isocitrate lyase, carries out part of the glyoxylate cycle, required for gluconeogenesis
YFL030W Putative alanine glyoxylate aminotransferase (serine pyruvate aminotransferase)
YGR067C; G4548 Protein of unknown function, has two tandem zinc finger domains
HXT5; YHR096C Member of the hexose transporter family of the major facilitator superfamily (MFS)
YIL057C Protein of unknown function
MBR1; YKL425; YKL440; YKL093W Protein involved in mitochondrial biogenesis
YKL187C Protein with similarity to 4-mycarosyl isovaleryl-CoA transferase
JEN1; YKL217W Pyruvate and lactate-proton symporter, member of the major facilitator superfamily (MFS)
PCK1; PPC1; JPM2; PEPC YKR097W Phosphoenolpyruvate carboxykinase (ATP), rate limiting gluconeogenic enzyme
IDP2; L9470.12; YLR174W Isocitrate dehydrogenase (NADP+), cytosolic
FBP1; L8039.18; YLR377C Fructose-1,6-bisphosphatase, gluconeogenic enzyme, activity is inhibited by protein kinase A-mediatedphosphorylation
CYB2; YM9958.08; YML054C Cytochrome b2 [L-(+)-lactate cytochrome c oxidoreductase] converts L-lactate to pyruvate
YMR107W; YM9718.06 Protein of unknown function, induced during aerobic growth
YMR206W; YM8325.07 Protein of unknown function
MLS1; N1921; YNL117; YNL117W Malate synthase 1, functions in glyoxylate cycle, has near identity to Dal7p
YNL194C; N1394 Protein with similarity to Sur7p
YNL195C; N1390 Protein of unknown function, induced during aerobic growth
YNR002C; N2029; (FUN34) Protein of unknown function, has strong similarity to Ycr010p and Ydr384p
CRC1; CAC YOR3193; O3193; YOR100C Mitochondrial carnitine carrier, member of the mitochondrial carrier family (MCF) of membranetransporters
GAC1; O4625; YOR178C Regulatory subunit for protein serine/threonine phosphatase Glc7p
LEE1; LPE18; YPL054W Protein of unknown function
PXA1; PAL1; SSH2; LPI1; PAT2; P2607; YPL147W Protein required for long-chain fatty acid transport across the peroxisomal membrane,member of the ATP-binding cassette (ABC) superfamily, has similarity to a human gene associated with adrenoleukodystrophy
YPL201C; P1870 Protein of unknown function, induced during aerobic growth
CSR2; MRG19; YP9367.10; YPR030W Protein of unknown function

Supplementary Table S12: Ribosomal Proteins
The ribosomal protein genes are listed. The average pattern ($\log_2$ ratio) is plotted upon transfer to YPD (which was followed for 5, 20 and 40 minutes after the transfer). The genes in the group, along with a one line description, are indicated.

Average expression: 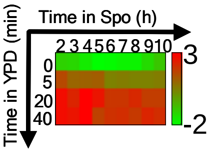
RPL19B; YBL0424; YBL027W Ribosomal protein L19 (yeast L23; YL14; rp15L; rp33; rat L19), nearly identical to Rpl19Ap
RPS8A; (RPS14A); (RP19); YBL06.05; YBL0613; YBL072C Ribosomal protein S8 (yeast S14; YS9; rp19; mammalian S8), identical to Rps8Bp
RPL23A; YBL0713; YBL087C Ribosomal protein L23 (yeast L17a; YL32; E. coli L14; rat L23), nearly identical to Rpl23Bp
RPS11B; YBR0501; YBR048W Ribosomal protein S11 (yeast S18, YS12, rp41, E. coli S17, human and rat S11), identical to Rps11Bp
RPS6B; (RPS10A); RPS101; YBR1244; YBR181C Ribosomal protein S6 (yeast S10; YS4; rp9; human, mouse, and rat S6), identical to Rps6Ap
RPL21A; URP1A; URP1; YBR1401; YBR191W Ribosomal protein L21 (rat L21), nearly identical to Rpl21Bp
RPS14A; CRY1; (RP59); YCR59; YCR031C Ribosomal protein S14A (rp59, E. coli S11, rat and human S14) involved in cryptopleurine resistance,nearly identical to Rps14Bp
RPS29B; D2542; YDL061C Ribosomal protein S29 (yeast S36; YS29; rat S29), similar to Rps29Ap
RPL31A; D2478; YDL075W Ribosomal protein L31 (yeast L34; YL36; YL28; rat L31), nearly identical to Rpl31Bp
RPP1A; RPLA1; (RPA1); YP1ALPHA; L12eIIA; D2450; YDL081C Acidic ribosomal protein P1A (A1 YP1alpha; E. coli L12eIIA; human and ratP1)
RPL13A; D2445; YDL082W Ribosomal protein L13 (rat L13), nearly identical to Rpl13Bp
RPS16B; D2442; YDL083C Ribosomal protein S16 (rp61R rat S16), identical to Rps16Ap
RPL35B; SOS2; D2170; YDL136W Ribosomal protein L35 (rat L35), identical to Rpl35Ap
RPL35A; SOS1; D1249; YDL191W Ribosomal protein L35 (rat L35), identical to Rpl35Bp
RPS11A; PZA156; D9813.03; D3278; YDR025W Ribosomal protein S11 (yeast S18; YS12; rp41; E. coli S17; human and rat S11), identical toRps11Bp
RPS13; YS15; YD9609.18; D4252; YDR064W Ribosomal protein S13 (yeast S27; YS15; rat S13)
RPP2B; RPLA4; RPL45; L12eIA; L36; YPA1; D9481.1; D9509.2; YDR382W Acidic ribosomal protein P2B (L45 YPA1; YL44C; YP2 beta E. coliL12eIA; human and rat P2)
RPL12B; D9461.7; YDR418W Ribosomal protein L12 (yeast L15; YL23; E. coli L11; rat L12a), identical to Rpl12Ap
RPS17B; RP51B; D9461.32; YDR447C Ribosomal protein S17 (rp51 rat S17), nearly identical to Rpl17Ap
RPS18A; YDR450W; RPS13C; D9461.35 Ribosomal protein S18 (E. coli S13; rat S18), identical to Rps18Bp
RPL27B; D8035.14; YDR471W Ribosomal protein L27 (yeast L27; mammalian L27), identical to Rpl27Ap
RPL37B; D9719.6; YDR500C Ribosomal protein L37 (yeast L46) (rat L37), nearly identical to Rpl37Bp
RPL12A; YEL054C Ribosomal protein L12 (yeast L15; YL23; E. coli L11; rat L12b), identical to Rpl12Bp
RPS24A; YER074W Ribosomal protein S24 (rat S24), identical to Rps24Bp
RPS8B; (RPS14B); (RP19); YER102W Ribosomal protein S8 (yeast S14; YS9; rp19; mammalian S8), identical to Rps8Ap
RPL23B; YER117W Ribosomal protein L23 (yeast L17a; YL32; E. coli L14; rat L23), nearly identical to Rpl23Ap
RPS26B; YER131W Ribosomal protein S26 (rat S26), nearly identical to Rps26Ap
RPL30; (RPL32); G3652; YGL030W Large-subunit ribosomal protein L30 (yeast L32; YL38; rp73; mouse and rat L30)
RPL24A; G3648; YGL031C Ribosomal protein L24A (yeast L30; rp29; YL21; rat L24), nearly identical to Rpl24Bp

RPL7A; YL8A; G3216; YGL076C Ribosomal protein L7 (yeast L6; YL8A; rp11; E. coli L30; rat L7), nearly identical to Rpl7Bp
RPL28; CYH2; G3101; YGL103W Ribosomal protein L28 (yeast L29; YL24; rp44; mouse and rat L27a)
RPS2; (RPS4); SUP44; G2893; YGL123W Ribosomal protein S2 (yeast S4; YS5; YP9; rp12; E. coli S5; rat S2)
RPL1B; SSM1B; SSM2; G2834; YGL135W Large subunit ribosomal protein L1 (rat L10A; eubacterial L1; archeal L1), identical to Rpl1Ap
RPL9A; RP25; YL11; G2505; YGL147C Ribosomal protein L9 (YL11 rp25; rp24; E. coli L6; rat L9), nearly identical to Rpl9Bp
RPS25A; RPS31A; G4046; YGR027C Ribosomal protein S25A (yeast S31; YS23; rp45; rat S25), identical to Rps25Bp
RPL26B; G4079; YGR034W Ribosomal protein L26 (yeast L33; YL33; rat L26), nearly identical to Rpl26Ap
RPL11B; G4620; YGR085C Ribosomal protein L11 (yeast L16; YL22; rp39B; E. coli L5; rat L11), nearly identical to Rpl11Ap
RPS23A; (RPS28A); G6178; YGR118W Ribosomal protein S23 (yeast S28; rp37; YS14; E. coli S12; rat S23), identical to Rps23Bp
RPL24B; G6635; YGR148C Ribosomal protein L24B (yeast L30; rp29; YL21; rat L24), nearly identical to Rpl24Ap
RPS0A; YST1; (NAB1); NAB1A; G7816; YGR214W Ribosomal protein S0 (E. coli S2; rat Sa), nearly identical to Rps0Bp
RPL14B; YHL001W Ribosomal protein L14 (mammalian L14), nearly identical to Rpl14Ap
RPS20; URP2; YHL015W Ribosomal protein S20 (E. coli S10; Xenopus S22; rat and human S20)
RPL8A; MAK7; YHL033C Ribosomal protein L8 (yeast L4; rp6; YL5; human, mouse, and rat L7a), involved in maintenance of M1 dsRNA virus;nearly identical to Rpl8Bp
RPL27A; YHR010W Ribosomal protein L27 (yeast L27; mammalian L27), identical to Rpl27Bp
RPS27B; SLO81; YHR021C Ribosomal protein S27 (yeast S27; rp61; YS20; mammalian S27), nearly identical to Rps27Ap
RPL42B; MAK18; SCL41B; YHR141C Ribosomal protein L42 (yeast L41; YL27; YP44; human and rat L36A), involved in replication of M1 and M2double-stranded RNA (dsRNA) viruses, identical to Rpl42Ap
RPS4B; (RPS4); (RPS7A); YHR203C Ribosomal protein S4 (yeast S7; YS6; rp5; rat and human S4), identical to Rps4Ap
RPL2B; RPL5A; YL6b; YI3299.13; YIL018W Ribosomal protein L2 (yeast L5; YL6; rp8; E. coli L2; tobacco L2; rat L8)
RPL34B; YIL052C Ribosomal protein L34 (rat L34), nearly identical to Rpl34Ap
RPL16A; YIL133C Ribosomal protein L16 (rp22), nearly identical to Rpl16Bp
RPL40A; UBI1; CEP52A; CST2; YIL148W Fusion protein whose N-terminal half is ubiquitin and whose C-terminal half is ribosomal protein L40(rat L40), identical to Rpl40Bp
RPS21B; (RPS26B); J0664; YJL136C Ribosomal protein S21 (yeast S26; YS25; rat S21), identical to Rps21Ap
TIF2; J0660; YJL138C Translation initiation factor 4A (eIF4A) of the DEAD box family, identical to Tif1p
RPL17B; J0493; YJL177W Ribosomal protein L17 (yeast L20; YL17; E. coli L22; rat and human L17), nearly identical to Rpl17Ap
RPS22A; (RPS24); J0355; YJL190C Ribosomal protein S22 (yeast S24; rp50; YS22; rat S15A), nearly identical to Rps22Bp
RPS5; J2045; YJR123W Ribosomal protein Rps5p (rp14 YS8; S2; mammalian S5) of the small subunit
RPS4A; (RPS4); (RPS7B); J2186; YJR145C Ribosomal protein S4 (yeast S7; YS6; rp5; rat and human S4), identical to Rps4Bp
RPL14A; YKL153; YKL006W Ribosomal protein L14 (mammalian L14), nearly identical to Rpl14Bp
YKL056C; YKL312 Protein possibly involved in cytoplasmic ribosome function, has similarity to translationally controlled tumor protein (TCTP) ofanimal cells and higher plants
RPS27A; YKL156W Ribosomal protein S27 (yeast S27; rp61; YS20; mammalian S27), nearly identical to Rps27Bp
RPL17A; YKL180W Ribosomal protein L17 (yeast L20; YL17; E. coli L22; rat and human L17), nearly identical to Rpl17Bp
RPS21A; (RPS26A); YKR057W Ribosomal protein S21 (yeast S26; YS25; rat S21), identical to Rps21Bp
TIF1; YKR059W Translation initiation factor 4A (eIF4A) of the DEAD box family, identical to Tif2p
RPL40B; UBI2; CEP52B; CST1; YKR414; YKR094C Fusion protein comprised of ribosomal protein L40 (C-terminal half) and ubiquitin(N-terminal half), (rat L40), identical to Rpl40Ap
RPL8B; KRB1; L0717; YLL045C Ribosomal protein L8 (yeast L4; rp6; YL5; human, rat, and mouse L7a),

involved in maintenance of M1 dsRNAvirus nearly identical to Rpl8Ap
RPL15A; L1755; YLR029C Ribosomal protein L15 (yeast L13; YL10; rp15R; rat L15), nearly identical to Rpl15Bp
RPS0B; YST2; NAB1B; L2118; YLR048W Ribosomal protein S0 (E. coli S2; rat Sb), nearly identical to Rps0Ap
RPL22A; YL31; L2168; YLR061W Ribosomal protein L22, similar to Rpl22Bp
RPL10; GRC5; QSR1; L2341; YLR075W Ribosomal protein L10 (yeast L9)
RPS31; UBI3; L9470.14; YLR167W Fusion protein comprised of ribosomal protein S31 at the C-terminal half fused to ubiquitin at the N-terminalhalf (yeast S37; YS24; rat S27a)
RPL37A; L9470.6; YLR185W Ribosomal protein L37 (yeast L46; rat L37), nearly identical to Rpl37Bp
RPS28B; L8479.5; YLR264W Ribosomal protein S28 (yeast S33; YS27; mammalian S28), nearly identical to Rps28Ap
RPL38; L8543.2; YLR325C Ribosomal protein L38
RPS25B; RPS31B; L8300.10; YLR333C Ribosomal protein S25B (yeast S31; YS23; rp45; rat S25), identical to Rps25Ap
SUR4; (APA1); VBM1; ELO3; SRE1; L8039.2; YLR372W Protein required for the conversion of 24-carbon fatty acids to 26-carbon fatty acids
RPS29A; L8084.11; YLR388W Ribosomal protein S29 (yeast S36; YS29; rat S29), similar to Rps29Bp
RPS1A; RP10A; (PLC1); L9753.9; YLR441C Ribosomal protein S1 (rp10 rat S3A), nearly identical to Rps1Bp
RPL6B; L9324.4; YLR448W Ribosomal protein L6 (yeast L17B; YL16B; human L6), nearly identical to Rpl6Ap
RPS17A; RP51A; YML024W Ribosomal protein S17 (rp51 rat S17), nearly identical to Rps17Bp
RPS18B; YML026C Ribosomal protein S18 (E. coli S13; rat S18), identical to Rps18Ap
RPS1B; RP10B; RP3B; PLC2; KRP-Y1; YML063W Ribosomal protein S1 (rp10 rat S3A), nearly identical to Rps1Ap
RPL6A; YML073C Ribosomal protein L6 (yeast L17A; YL16A; human L6), nearly identical to Rpl6Bp
ASC1; BEL1; CPC2; YM9718.15; YMR116C Ribosomal protein of the 40S ribosomal subunit that influences translational efficiency and cell size,contains two WD (WD-40) repeats
RPL13B; YM9375.11; YMR142C Ribosomal protein L13 (rat L13), nearly identical to Rpl13Ap
RPL36A; YM9646.06; YMR194W Ribosomal protein L36 (yeast L39; YL39; rat L36), nearly identical to Rpl36Bp
GUA1; YM8261.11; YMR217W GMP synthetase, catalyzes the amination of xanthine monophosphate to guanine monophosphate in the guaninebranch of the purine biosynthesis pathway
RPL20A; YM9408.04; YMR242C Ribosomal protein L20, nearly identical to Rpl20Bp
RPL9B; N2406; YNL1611; YNL2406; YNL067W Ribosomal protein L9 (YL11 rp25; rp24; E. coli L6; rat L9), nearly identical to Rpl9Ap
RPL16B; N2377; YNL069C Ribosomal protein L16 (rp23), nearly identical to Rpl16Ap
RPS7B; N2212; YNL096C Ribosomal protein S7 (rp30 human S7; Xenopus S8), nearly identical to Rps7Ap
RPL42A; SCL41A; N1722; YNL162W Ribosomal protein L42 (yeast L41; YL27; YP44; human and rat L36A), identical to Rpl42Bp
RPS3; RP13; SUF14; N1653; YNL178W Ribosomal protein S3 (rp13 YS3; mammalian S3), has a possible KH domain
SSB2; N1333; YNL209W Heat shock protein of HSP70 family, cytoplasmic
RPL18B; RP28B; N0425; YNL0425; YNL301C Ribosomal protein L18 (rp28 rat L18), identical to Rpl18Ap
RPS19B; RP55B; (RPS16B); N0422; YNL0422; YNL302C Ribosomal protein S19 (rp55 YS16B; rat S19), nearly identical to Rps19Ap
RPP2A; RPLA2; RPL44; (RPA2); L12eIB; L35; O2060; YOL039W Acidic ribosomal protein P2A (L44 A2; YP2alpha; E. coli L12eIB; humanP2alpha), plays a role in the elongation step
RPS15; RPS21; O2057; YOL040C Ribosomal protein S15 (yeast S21; rp52; E. coli S19; rat S15; RIG protein)
RPL18A; RP28A; O0565; YOL120C Ribosomal protein L18 (rp28 rat L18), identical to Rpl18Bp
RPS19A; RP55A; (RPS16A); O0559; YOL121C Ribosomal protein S19 (rp55 YS16A; rat S19), nearly identical to Rps19Bp
RPL25; O0534; YOL127W Ribosomal protein L25 (YL25 rp61L; E. coli L23; rat L23a)
RPL3; TCM1; MAK8; O2812; YOR29-14; YOR063W Ribosomal protein L3 (YL1 rp1; rat L3) , responsible for trichodermin resistance andinvolved in maintenance of dsRNA viruses
RPS7A; RP30; YOR3177; O3177; YOR096W Ribosomal protein S7 (rp30 human S7; Xenopus S8), nearly

identical to Rps7Bp
RPS28A; O3598; YOR167C Ribosomal protein S28 (yeast S33; YS27; mammalian S28), nearly identical to Rps28Bp
RPL33B; O5224; YOR234C Ribosomal protein L33 (yeast L37; YL37; rp47; rat L35A), nearly identical to Rpl33Ap
RPS10A; O5611; YOR293W Ribosomal protein S10 (rat S10), nearly identical to Rps10Bp
RPL20B; O6116; YOR312C Ribosomal protein L20, nearly identical to Rpl20Ap
RPS12; RS12; O6673; YOR369C Ribosomal protein S12 (rat S12)
EGD1; CST25; P7102.13; YPL037C Beta subunit of the nascent polypeptide-associated complex
RPL21B; URP1B; LPF6; YPL079W Ribosomal protein L21 (rat L21), nearly identical to Rpl21Ap
RPS6A; (RPS10B); RPS102; LPG18; YPL090C Ribosomal protein S6 (yeast S10; YS4; rp9; human, mouse, and rat S6), identical to Rps6Bp
RPL33A; LPI4; P2625; YPL143W Ribosomal protein L33 (yeast L37; YL37; rp47; rat L35A), nearly identical to Rpl33Bp
RPL1A; SSM1A; SSM1; P1740; YPL220W Large subunit ribosomal protein L1 (rat L10A; eubacterial L1; archeal L1), identical to Rpl1Bp
RPL11A; P8283.14; YPR102C Ribosomal protein L11 (yeast L16; YL22; rp39A; E. coli L5; rat L11), nearly identical to Rpl11Bp
RPS23B; (RPS28B); P9659.9; YPR132W Ribosomal protein S23 (yeast S28; rp37; YS14; E. coli S12; rat S23), identical to Rps23Ap
RPL19A; YBR084C-A Ribosomal protein L19 (yeast L23; YL14; rp15L; rp33; rat L19), nearly identical to Rpl19Bp
RPL34A; YER056C-A Ribosomal protein L34 (rat L34), nearly identical to Rpl34Bp
RPL2A; YL6; YFR031C-A Ribosomal protein L2 (Yeast L5; YL6; rp8; E. coli L2; tobacco L2; rat L8)

Supplementary Table S13: Genes induced in a timely dependent manner

The average pattern (log_2ratio) is plotted upon transfer to YPD (which was followed for 5, 20 and 40 minutes after the transfer). The genes in the group, along with a one line description, are indicated.

Template genes used to create this group are: *YIR038C, YHR087W, YDL222C, YMR250W, SIP18, SPS100 and YMR322C*

Average expression of genes induced in a timely dependent manner

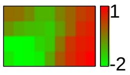

YBR116C; YBR0911 Protein of unknown function
TKL2; YBR0912; YBR117C Transketolase 2
YBR241C; YBR1625 Member of the hexose transporter family of the major facilitator superfamily (MFS)
DIA3; D2815; YDL024C Protein with similarity to acid phosphatases
YDL072C; D2492 Protein of unknown function
YDL124W; D2240 Protein of unknown function
SFA1; SFA D1473; YDL168W Long-chain alcohol dehydrogenase (ADH) with similarity to mammalian ADH class III
YDL204W; D1062 Protein of unknown function
YDL218W; D0879 Protein of unknown function induced during aerobic growth, has weak similarity to Ynr061p
YDL221W; D0855 Protein of unknown function
YDL222C; D0850 Protein with similarity to Sur7p
YDL223C; D0843 Protein with repeated region characteristic of neuraxin and Map1b proteins, also has ATP/GTP-binding site motif A
PST2; D3422; YD9673.02; YDR032C Protein with similarity to Ycp4p and S. pombe OBR1 brefeldin A resistance protein
YDR070C; YD8554.03; D4275 Protein of unknown function
TPS2; HOG2; PFK3; D4416; YD8554.07; YDR074W Trehalose-6-phosphate phosphatase, component of the trehalose-6-phosphatasesynthase/phosphatase complex
YDR119W; YD9727.14 Member of the 14-spanner drug:[H+] antiporter (DHA14) family of multidrug-resistance (MFS-MDR) proteins in the majorfacilitator superfamily (MFS)
YDR154C Protein of unknown function
DIT1; D9509.21; YDR403W First enzyme in the pathway for biosynthesis of dityrosine in the outer layer of the spore wall expressed late (10-16hr) in sporulation
HSP12; GLP1; YFL014W Heat shock protein of 12 kDa, induced by heat, osmotic stress, oxidative stress and in stationary phase
OCH1; NGD29; G3626; YGL038C Alpha-1,6-mannosyltransferase, involved in initiation of mannose outer chain elongation of N-linkedoligosaccharides of type Man[9]GlcNac[2]
YGL059W; G3441 Protein with similarity to human branched-chain alpha-ketoacid dehydrogenase kinase transcription induced by the drug FK506in a GCN4-dependent manner
STF2; G3858; YGR008C ATPase stabilizing factor, binds to F0-ATPase facilitates binding of inhibitor and 9 kDa protein to F1-ATPase
YGR043C; G4301 Protein of unknown function, may be involved in signal transduction has strong similarity to Tal1p
YGR086C; G4622 Protein of unknown function, expression is induced by high salt and low pH
CTT1; G4628; YGR088W Catalase T (cytosolic)
YGR130C; G6382 Protein with similarity to Entamoeba histolytica myosin heavy chain PIR:L03534
GND2; G9170; YGR256W 6-Phosphogluconate dehydrogenase, decarboxylating, converts 6-phosphogluconate + NADP to ribulose-5-phosphate+ NADPH + CO2
YHR087W Protein of unknown function
SPS100; YHR139C Sporulation specific protein involved in spore wall formation, first appears at 12h of sporulation
NVJ1; VAB36; YHR195W Vac8p-binding protein located at sites of contact between the

nucleus and the vacuole
XBP1; YIL101C Stress-induced transcriptional repressor
OM45; YIL136W Protein of the outer mitochondrial membrane
HYR1; GPX3; YIR037W Glutathione peroxidase involved in oxidative stress response
GTT1; YIR038C Glutathione transferase
TOR1; DRR1; J1803; YJR066W Phosphatidylinositol kinase (PI kinase) homolog, involved in cell growth and sensitivity to theimmunosuppressant rapamycin
YJR096W; J1926 Protein with similarity to aldolase reductase
YKL050C; YKL301 Protein of unknown function, has significant similarity to Ymr031p
YKL098W; YKL448 Protein of unknown function
YKL187C Protein with similarity to 4-mycarosyl isovaleryl-CoA transferase
YLR164W; L9632.1 Protein with strong similarity to Sdh4p
MSC3; L8167.1; YLR219W Protein of unknown function that affects meiotic homologous chromatid recombination
FBP1; L8039.18; YLR377C Fructose-1,6-bisphosphatase, gluconeogenic enzyme, activity is inhibited by protein kinase A-mediatedphosphorylation
GLO1; YM9571.15; YML004C Glyoxalase I, catalyzes condensation of hemimercaptal adduct of methylglyoxal with glutathione to formS-D-lactoylglutathione
MSC1; YM4987.07; YML128C Protein of unknown function that affects meiotic homologous chromatid recombination
PLB2; YM8270.08; YMR006C Phospholipase B2 (lysophospholipase), releases fatty acids from lysophospholipids
YMR084W; YM9582.09A Protein of unknown function, YMR085W and YMR084W are possible parts of a pseudogene with similarity to Gfa1p
YMR118C; YM9718.17 Putative succinate dehydrogenase, has similarity to Sdh3p
SIP18; YM8010.05; YMR175W Protein induced by osmotic stress
GAD1; YM9920.04; YMR250W Glutamate decarboxylase
YMR251W; YM9920.05 Putative paralog of Ecm4p
YMR322C; YM9924.14 Protein of unknown function, may be involved in cell stress
YNL134C; N1214; N1847 Protein with similarity to C. carbonum toxD gene
MDG1; N1673; YNR3; YNL173C Protein involved in signal transduction in the mating response
YOR173W; O3625 Protein of unknown function
PUT4; O6345; YOR348C High affinity proline permease, also transports alanine and glycine
YOR391C; O6784 Protein of unknown function
YPL004C; YP8132.09; LPA13 Protein with weak similarity to tropomyosin
GRE1; P1715; YPL223C Protein induced by osmotic stress
USV1; P1421; YPL230W Putative finger transcripton factor, has a two tandem C2H2-type zinc fingers
YPL280W; P0307 Protein of unknown function

Supplementary Table S14: Genes induced in response to YPD in committed cells

The average pattern (log_2ratio) is plotted upon transfer to YPD (which was followed for 5, 20 and 40 minutes after the transfer). The genes in the group, along with a one line description, are indicated.

Template genes used to create this group are: SRA1, SWI6, YLR241W, YMR075W, YBR042C, YJL120W, YLR433C and YDR166C

Average expression 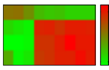
EDE1; YBL0520; YBL0501; YBL047C Protein with role in endocytosis, has similarity to cytoskeletal proteins Uso1p and Pan1p and to mousetyrosine kinase substrate eps15
YBR042C; CST26; YBR0412 Putative acyltransferase with similarity to Ydr018p
MRPL27; YML27; YBR2019; YBR282W Mitochondrial ribosomal protein of the large subunit
YDL062W; D2540 Protein of unknown function, questionable ORF
YDL189W; D1260 Protein of unknown function
YRB1; HTN1; SFO1; (RBP1); CST20; YD8119.08; D2960; YDR002W Protein that stimulates GTPase activity of Gsp1p and Gsp2p in thepresence of Rna1p
SEC5; MCD7; YD9489.01; YDR166C Component of exocyst complex, 107 kDa, required for exocytosis
YDR332W; D9798.1 Member of the DEAD-box family of predicted RNA helicases
ARO80; D9461.10; YDR421W Positive transcription regulator of ARO9 and ARO10, member of the Zn2Cys6 transcription factor family
YDR425W; D9461.13 Protein of unknown function
CAJ1; YER048C Homolog of E. coli DnaJ, has a leucine zipper
ADK2; ADK3; AKY3; PAK3; YER170W Adenylate kinase (GTP:AMP phosphotransferase), mitochondrial
MSB2; G4017; YGR014W Protein for which overproduction suppresses bud emergence defect of cdc24 mutant
YGR127W; G6370 Protein with similarity to mouse T10 protein PIR:S37448
DIE2; ALG10; ORF525; G8547; YGR227W Glucosyltransferase involved in the terminal glucosylation step of the lipid-linked oligosaccharide
COQ6; G9165; YGR255C Monooxygenase required for coenzyme Q (ubiquinone) biosynthesis
SMF2; YHR050W Probable manganese transporter
BCY1; SRA1; (REG1); YIL033C Regulatory subunit of cAMP-dependent protein kinases
YJL120W; J0734 Protein of unknown function
YJR084W; J1860 Protein of unknown function
YKL111C Protein of unknown function questionable ORF
YKL131W Protein of unknown function questionable ORF
SDH1; SDHA HAR2; YKL602; YKL148C Succinate dehydrogenase (ubiquinone) flavoprotein (Fp) subunit, converts succinate plus ubiquinoneto fumarate plus ubiquinol in the TCA cycle
SAC1; RSD1; YKL212W Polyphosphoinositide phosphatase, required for transport of ATP into ER which plays a role in Golgi function and actincytoskeleton organization
YKR035C Protein of unknown function
COX17; L1343; YLL009C Cytoplasmic protein involved in delivery of copper ions to mitochondrial cytochrome oxidase
EFR4; L9354.8; L2941; YLR114C Protein with weak similarity in the C-terminus to Drosophila melanogaster bicaudal-D protein
CLF1; SYF3; L9233.7; L2952; YLR117C Essential pre-mRNA splicing factor with similarity to Drosophila crooked neck (crn) protein, hastetratricopeptide (TPR) repeats
SAM1; ETH10; L9470.9; YLR180W S-adenosylmethionine synthetase 1
SWI6; SDS11; PSL8; L9470.8; YLR182W Transcription factor that participates in the SBF complex (Swi4p-Swi6p) for regulation at the cell cyclebox (CCB) and in the MBF complex (Mbp1p-Swi6p) for regulation at the Mlu1 cell cycle box (MCB)

Supplementary Table S15: Genes repressed in response to YPD in committed cells

The average pattern (log_2ratio) is plotted upon transfer to YPD (which was followed for 5, 20 and 40 minutes after the transfer). The genes in the group, along with a one line description, are indicated.

Template genes used to create this group are: KAP104, YBR175W, YMR306CA, YCL066W, YOL032W and YKR010C

Average expression	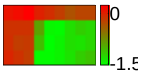

LTE1; MSI2; YAL024C GDP/GTP exchange factor
KAP104; YBR0224; YBR017C Transportin, beta-karyopherin involved in nuclear transport of mRNA-binding proteins
YBR051W; YBR0504a Protein of unknown function questionable ORF
SPT7; GIT2; EFR2; YBR0739; YBR081C Component of the nucleosomal histone acetyltransferase (Spt-Ada-Gcn5-Acetyltransferase or SAGA)complex
YBR113W; YBR0908e Protein of unknown function
YBR175W; YBR1237 Protein of unknown function, has several WD (WD-40) repeats
PCH2; YBR1308; YBR186W Protein required for cell cycle arrest at the pachytene stage of meiosis in a zip1 mutant, has similarity to Rpt5p andNSF vesicular fusion protein and other members of the AAA family of ATPases
CDC47; MCM7; (MIS1); YBR1441; YBR202W Member of MCM/P1 family of proteins involved in DNA synthesis initiation
THI2; PHO6; YBR1624; YBR240C Zinc-finger regulatory protein for thiamine pyrophosphokinase (THI80) expression
YBR300C; YBR2120 Protein of unknown function
YCL034W; YCL186; LSB5 Protein with possible role in the regulation of actin cytoskeletal organization
YCL063W; YCL062W Protein involved in sensitivity to certain drugs has similarity to plant aminocyclopropane-1-carboxylate synthase
ALPHA1; MATALPHA1; HMLALPHA1; YCR040W; YCL066W Regulatory protein Alpha1p, with Mcm1p, activates alpha-specific genes
YCR097wa
YDL038C; D2726 Protein of unknown function
SSB1; YG101; D0819; YDL229W Heat shock protein of HSP70 family, involved with the translational machinery
BUD26; YD8419.08; YDR241W Protein of unknown function
MPS2; MMC1; G3219; YGL075C Protein of the nuclear envelope/endoplasmic reticulum required for spindle pole body assembly and normalchromosome segregation
MGA1; G9130; YGR249W Protein with similarity to heat shock transcription factors
MUC1; FLO11; YIR019C Cell surface flocculin, required for invasive and pseudohyphal growth
DAL3; YIR032C Ureidoglycolate hydrolase involved in utilization of purines as nitrogen sources by converting (-)-ureidoglycolate + H20 toglyoxylate + 2 NH3 + CO2
ASF1; J0755; YJL115W Component of replication-coupling chromatin assembly factor (RCAF), target of the Rad53p-dependent DNA damageresponse
YNK1; YNK NDK1; YKL333; YKL067W Nucleoside diphosphate kinase, responsible for synthesis of all nucleoside triphosphates except ATP
TOF2; YK109; YKR010C Protein that interacts with DNA topoisomerase I
YLL037W; L0910 Protein with weak similarity to human platelet-activating factor receptor
KRE34; L_C144 YLR317W Protein of unknown function questionable ORF
TOM37; PET3027; MAS37; YM9796.13; YMR060C Component of the mitochondrial outer membrane receptor (TOM) complex, required only athigh temperature, contains tetratricopeptide (TPR) repeats
SGS1; (TPS1); YM9646.02; YMR190C DNA helicase of DEAD/DEAH family, has similarity to

the human Werner's and Bloom's syndromes genesand to E. coli RecQ 3'-5' DNA helicase
YMR206W; YM8325.07 Protein of unknown function
YNL089C; N2242 Protein of unknown function, questionable ORF
YNL105W; N2169 Protein of unknown function, questionable ORF
YNL203C; N1358 Protein with weak similarity to Bacillus subtilis CDPdiacylglycerol--serine O-phosphatidyltransferase
THI12; N0295; YNL332W Biosynthetic enzyme involved in thiamine utilization pathway, involved in conversion of hydroxymethyl-pyrimidineprecursor to thymidine Thi5p, Thi11p, Thi12p and Thi13p are nearly identical
YOL032W; O2133 Protein of unknown function
YOL163W; O0230 Protein with weak similarity to Pseudomonas putida phthalate transporter
SGT2; UNF346; O2537; YOR007C Protein of unknown function, contains tetratricopeptide (TPR) repeats
SEN54; LPF3; YPL083C tRNA splicing endonuclease, alpha subunit
YPL099C; LPG9 Protein of unknown function
RNY1; LPH4; YPL123C Ribonuclease of the ribonuclease T2 family
ATP20; SU_G YP9531.14; YPR020W F1F0-ATP synthase complex F0 membrane domain g subunit
YPR130C; P9659.10A Protein of unknown function questionable ORF
DPB2; P9705.7; YPR175W DNA polymerase epsilon 80 kDa subunit
YMR306C-A Protein of unknown function

VPS34; VPT29; VPL7; END12; STT8; L9672.10; YLR240W Phosphatidylinositol 3-kinase (PI 3-kinase) required for vacuolar protein sorting,activated by protein kinase Vps15p
YLR241W; L9672.9 Protein of unknown function
IMH1; SYS3; L2142.5; YLR309C Probable coiled-coil protein, single copy and multicopy suppressor of ypt6, contains a Golgi-localization (GRIP)domain
TUS1; SOP10; L9576.5; YLR425W Protein with similarity to Rom1p and Rom2p GDP-GTP exchange factors
CRN1; L9576.2; YLR429W Coronin, actin-binding protein, contains WD (WD-40) repeats
CNA1; CMP1; L9753.6; YLR433C Calcineurin catalytic (A) subunit, protein serine/threonine phosphatase 2B (PP2B), member of the PPP family ofprotein phosphatases
YML029W Protein of unknown function
CMP2; CNA2; (TSM4572); YM9958.05; YML057W Calcineurin catalytic (A) subunit, protein serine/threonine phosphatase 2B (PP2B), member ofthe PPP family of protein phosphatases
ALO1; ALO YML086C D-arabinino-1,4-lactone oxidase (L-galactono-gamma-lactone oxidase)
YMR045C
YMR075W; YM9916.14 Protein of unknown function, contains a PHD domain
YMR140W; SIP5; YM9375.0 Protein interacting with Snf1p and Reg1p
CUE1; YM8156.06; YMR264W Protein that recruits the soluble ubiquitin-conjugating enzyme Ubc7p to the membrane
YNL134C; N1214; N1847 Protein with similarity to C. carbonum toxD gene
MRPL19; YML19; N1623; YNL185C Mitochondrial ribosomal protein of the large subunit
YOR024W; YOL303.9; OR26.14; O2648 Protein of unknown function
YOR121C; O3272 Protein of unknown function
MYO2; CDC66; O6167; YOR326W Myosin heavy chain, class V, involved in polarized growth and secretion
YPL025C; LPB6 Protein of unknown function
SEC16; LPF1; LST10; YPL085W Hydrophilic protein required for vesicle formation in ER to Golgi transport
CSR2; MRG19; YP9367.10; YPR030W Protein of unknown function
DIB1; SNU15; (CDH1); P9513.12; YPR082C Component of the U4/U6.U5 snRNP, also required for mitotic spindle formation or function
YPR090W; P9513.3 Protein of unknown function
PRP4; RNA4; P9705.6; YPR178W U4/U6 snRNA-associated splicing factor, member of WD (WD-40) repeat family
SMX3; SMF P9705.4; YPR182W Spliceosomal snRNA-associated Sm core protein, also likely associated with telomerase TLC1 RNA

Supplementary Table S16

Strain List. Strains used in this study

Name	Full Genotype[a]	Provider
NKY1551	*MATa/MATα ho::LYS2/ho::LYS2, ura3/ura3, lys2/lys2, leu2::hisG/leu2::hisG, arg4-Nsp/arg4-Bgl, his4x::LEU2-URA3 /his4B::LEU2*	N. Kleckner
JPY214	*MATa/MATα ho::LYS2/ho::LYS2 lys2/lys2 ura3/ura3 leu2/leu2 his4X-ADE2-his4B/his4X-ADE2-his4B ade2/ade2 Δsum1::kanMX6/Δsum1::kanMX6*	J. Segall
NKY1712	*MAT a ho::LYS2 leu2, trp1, ura3, lys2, ho::LYS2, arg4,*	N. Kleckner
NE30	*MATα ho::hisG leu2, trp1, ura3, lys2.ade2*	G. Simchen
GF18	*MATa/MATα pSPS4-3HA-NDT80/NDT80, ho::LYS2/ho::LYS2, lys2/lys2 leu2/leu2, trp1/trp1, ura3/ura3, ARG4/arg4, URA3/ura3, ADE2/ade2*	Constructed in this study
NKY1059	*MAT a lys2,ura3,leu2,ade2,his4,ho::hisG*	N. Kleckner
NKY561	*MATα* lys2,ura3(PsI-SmaI)::hisG, leu2::hisG, trp1::hisG, ho::hisG	N. Kleckner
DS1	*MATa/MATα lys2,ura3,leu2,ho::hisG*	Constructed in this study by mating of NKY1059 and NKY561

[a] All strains are in the SK1 genetic background

Supplementary Table S17: Media used in the present study

Minimal Supplemented SD plates (for diploid selection)
2% glucose
0.17% yeast nitrogen base
0.5% ammonium sulfate
1.5% agar
supplemented with Uracil, Leucine and Tryptophan

His- SD plates (for recombination assay)
2% glucose
0.17% yeast nitrogen base
0.5% ammonium sulfate
1.5% agar
supplemented with all amino acids except His

Sporulation plates
0.25% yeast extract
1.5% Potassium Acetate
0.25% Glucose
1.5% agar
supplemented with all amino acids

YPD
2% yeast extract
4% Bactopeptone
4% glucose

YPA
1% yeast extract
1% potassium acetate
2% Bactopeptone

SPM
0.3%-1% potassium acetate
0.02% rafinose

Glucose
4% Glucose
