# Supplementary material for: Modulation of the transcription regulatory program in yeast cells committed to sporulation
Source: Genome Biol. 2006 Mar 8;7(3):R20. doi: 10.1186/gb-2006-7-3-r20 (PMC1557749; doi:10.1186/gb-2006-7-3-r20)

ViewModules

ViewModules is a matlab (6.5) program to view sporulation microarray experiments discussed in the paper "Modulation of the transcription regulatory program in yeast cells committed to sporulation".

<u>To install (on a PC)</u>:
Create a folder and extract all files in the ViewModules.zip file to that folder.
Optional: add the folder to matlab path (launch matlab software, choose File from the menu, and set path. Click the "Add Folder" button and choose the desired folder. Click the "Save" button).

<u>To start the program</u>:

1. If you added the folder to matlab's path you can be in any folder. Otherwise browse for the required folder.
2. Type: ViewModules

<u>Instructions</u>:
I. To view the expression profile of a certain gene:
Type the gene name or open reading frame name in the "type a gene" field and press enter. In case the gene was not found or there are more then one genes that begin with the typed name, a message will appear.

General:
All profiles are in $\log_2$(ratios).

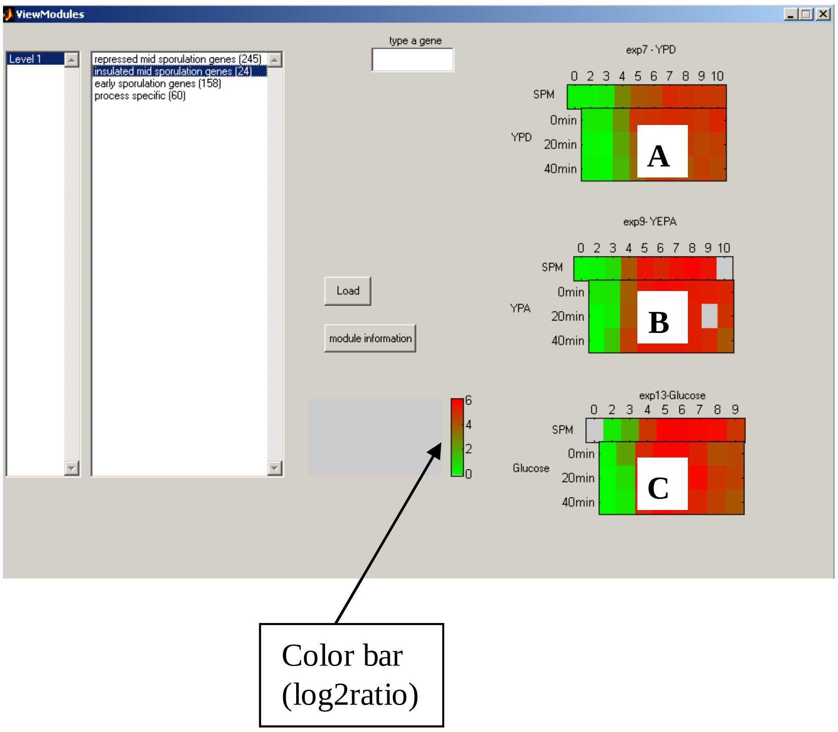

In A-C the data of our experiments is presented in the following way:

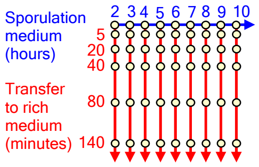

A-C differ in the medium to which the cells were transferred to The medium and the times are indicated left to each image.

II. To view the expression of our modules, GO groups or our sporulation groups:

1. Press the "Load" button and choose one of the following files:

**File name**	**To view the average expression, or the expression of genes of:**
mod_ave_modules	modules, obtained from the Iterative Signature Algorithm developed in our group
mod_ave_sporulation_groups	Group of genes that were defined according to the expression pattern in our sporulation and transfer to YPD experiments
mod_ave_GO_bp	Groups of GO - biological process
mod_ave_GO_cc	Groups of GO – cellular component
mod_ave_GO_mf	Groups of GO – molecular function

2. Always after choosing a file click on one of the levels (also if there is only one possibility):

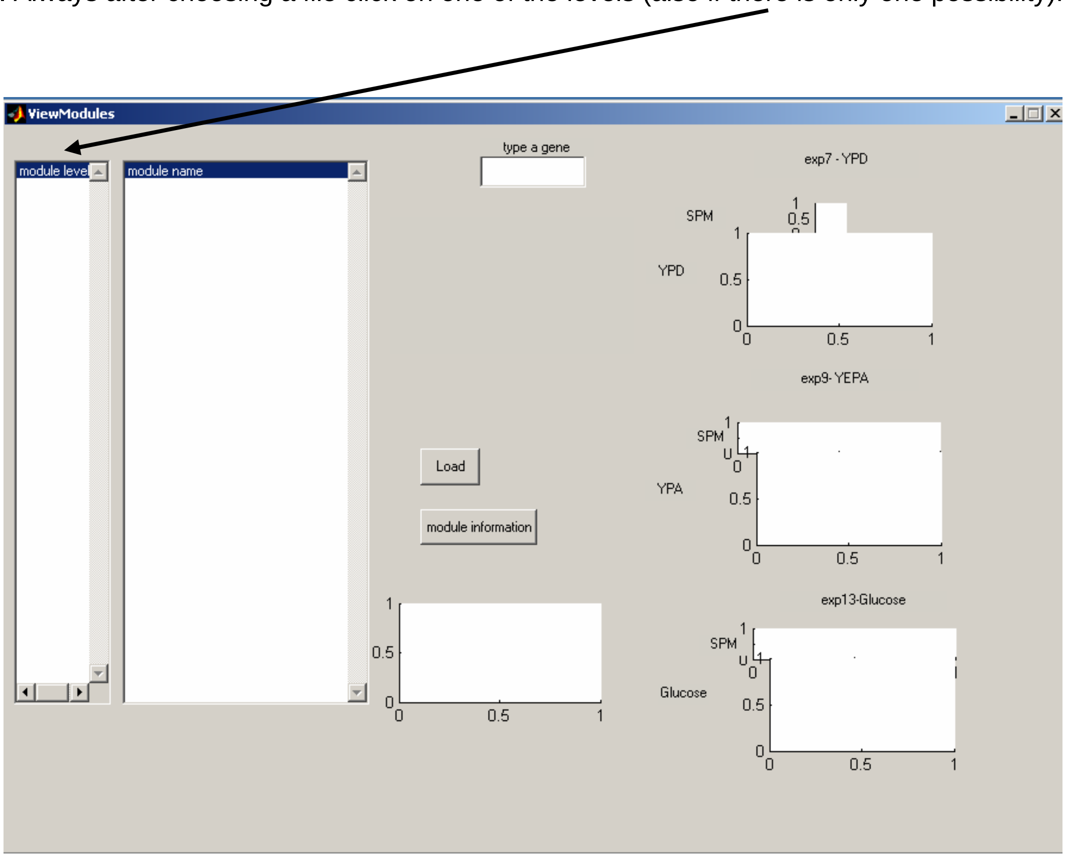

3. Now you can choose any desired category (number of genes is indicated in parentheses for each category).

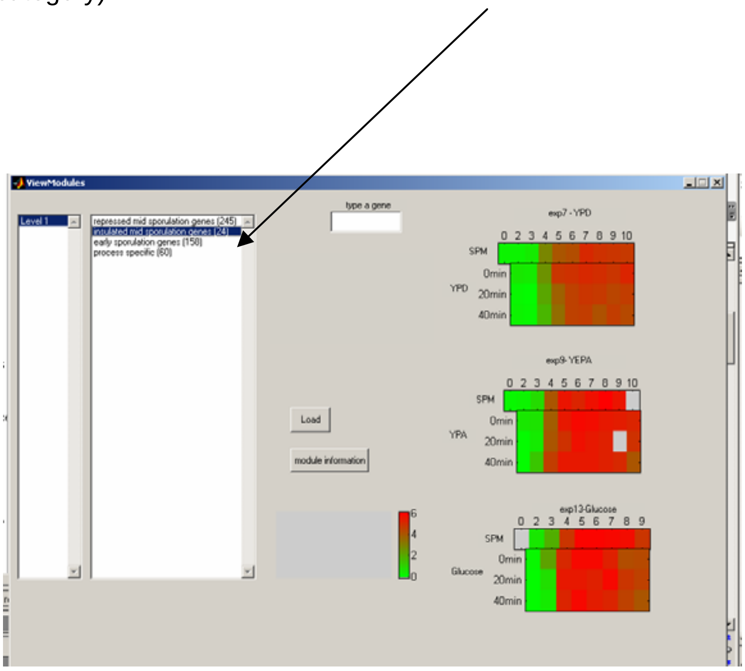

The categories are ordered according to the extent of change in our microarray experiments.

You will then see on the screen the average expression of the genes in the chosen category. Please note that looking solely on the average might be misleading (as the groups are not always homogenous).

4. After choosing the desired category, you can view the expression of each of the genes in the chosen category by clicking on the "module information" button. Another window with a list of the genes in the category will appear. Clicking on each gene in the list, will display the expression profile of the gene in our experiments (same format, again in log_2(ratio)).

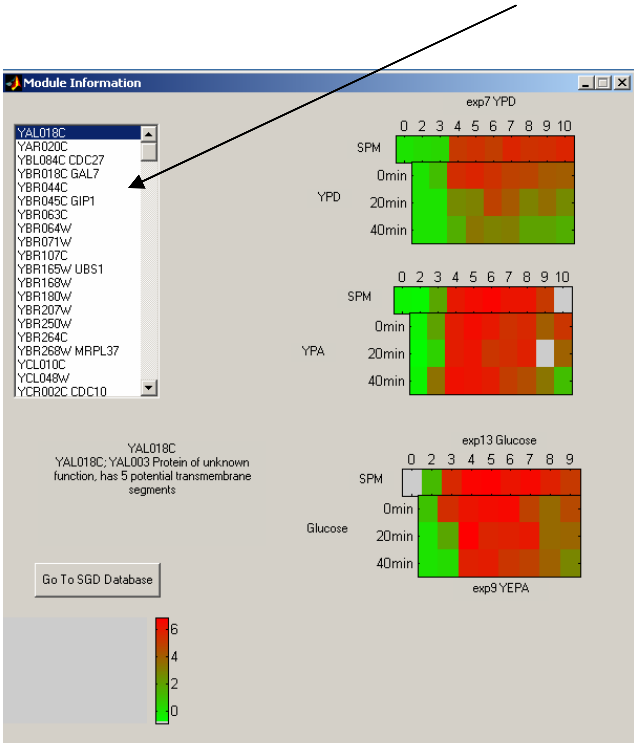

Supplement: Additional File 5 — A matlab program that enables the expression data discussed in this article to be viewed. Also contains a help file: 'ViewModules help.pdf' [file gb-2006-7-3-r20-S5.zip › ViewModules help.pdf]
