# Supplementary material for: Modulation of the transcription regulatory program in yeast cells committed to sporulation
Source: Genome Biol. 2006 Mar 8;7(3):R20. doi: 10.1186/gb-2006-7-3-r20 (PMC1557749; doi:10.1186/gb-2006-7-3-r20)
Supplement: Additional File 4 — Normalized data of the present study (in log2 ratios) [file gb-2006-7-3-r20-S4.zip › NormalizedData.doc]

The Normalized folder contains 6 files, one for each experiment. Each file contains normalized log_2_(ratios). For details of the normalization see the MIAME checklist file.

| **File name** | **Brief description of the time course in each file** |
| --- | --- |
| transfer of w.t. to YPD.xls | Wild type SK1 yeast cells were embarked upon sporulation. The cells were followed at different stages of the sporulation process (2, 3, 4, 5, 6, 7, 8, 9 and 10 hours- these samples are indicated in the file as sporulation followed by the hour). At each hour, part of the culture was centrifuged and resuspended in YPD. Each YPD culture was monitored immediately after the transfer, after 20 and 40 minutes (see MIAME checklist for more details). These samples are indicated in the file by: X R Y- transfer of cells after X hours in sporulation medium to YPD, Y minutes after the transfer. For example: 5R20 means that cells were sporulated for 5 hours, transferred to YPD and monitored 20 minutes after the transfer. |
| transfer of w.t. to YPA.xls | Wild type SK1 yeast cells were embarked upon sporulation. The cells were followed at different stages of the sporulation process (2, 3, 4, 5, 6, 7, 8 and 9 hours- these samples are indicated in the file as sporulation followed by the hour). At each hour, part of the culture was centrifuged and resuspended in YPA. Each YPA culture was monitored immediately after the transfer, after 20 and 40 minutes (see MIAME checklist for more details). These samples are indicated in the file by: X R Y- transfer of cells after X hours in sporulation medium to YPA, Y minutes after the transfer. For example: 5R20 means that cells were sporulated for 5 hours, transferred to YPA and monitored 20 minutes after the transfer. |
| transfer of w.t. to glucose.xls | Wild type SK1 yeast cells were embarked upon sporulation. The cells were followed at different stages of the sporulation process (2, 3, 4, 5, 6, 7, 8 and 9 hours- these samples are indicated in the file as sporulation followed by the hour). At each hour, part of the culture was centrifuged and resuspended in glucose (4%). Each glucose culture was monitored immediately after the transfer, after 20 and 40 minutes (see MIAME checklist for more details). These samples are indicated in the file by: X R Y- transfer of cells after X hours in sporulation medium to glucose, Y minutes after the transfer. For example: 5R20 means that cells were sporulated for 5 hours, transferred to glucose solution and monitored 20 minutes after the transfer |
| transfer of pSPS4-NDT80 to YPD.xls | *pSPS4-NDT80* SK1 yeast cells were embarked upon sporulation. The cells were followed after being 5.5 hours in sporulation media. At this stage, part of the culture was centrifuged and resuspended in YPD and was monitored immediately after the transfer, after 20 and 40 minutes (see MIAME checklist for more details). |
| transfer of sum1 deletion to YPD.xls | *sum1* deleletion SK1 yeast cells were embarked upon sporulation. The cells were followed after being 6 hours in sporulation media. At this stage, part of the culture was centrifuged and resuspended in YPD and was monitored. 20 and 40 minutes after the transfer (see MIAME checklist for more details). |
| Transfer of spores to YPD | Wild type SK1 cells (DS1) were grown to saturation in YPD (2% yeast extract, 4% bactopeptone, 4% glucose) for 24 hours and plated on sporulation plates (0.25% yeast extract, 1.5% Potassium acetate, 0.05% glucose supplemented with all amino acids). 3 days old asci were harvested and suspended in YPD at 30^°^C to initiate spore germination. RNA for microarray hybridization was extracted at the indicated times. |
